# 4D nanoimaging-guided smart therapeutics: spatiotemporal control of disease microenvironments

**DOI:** 10.1039/d6ra05783g

**Published:** 2026-09-09

**Authors:** Arnabjyoti Deva Sarma, Moitrayee Devi, Thomas J. Webster, Dinesh Kumar, Neeraj Choudhary, Suresh Babu Kondaveeti

**Affiliations:** a Faculty of Allied and Healthcare Sciences, Assam Down Town University Sankar Madhab Path, Gandhi Nagar, Panikhaiti Guwahati Assam 781026 India sarma.arnab1990@gmail.com moitrayeedevi@gmail.com; b School of Health Sciences and Biomedical Engineering, Hebei University of Technology Tianjin China thomas_webster@brown.edu; c School of Engineering, Saveetha University Chennai India; d Department of Pharmacy, University of the Basque Country Vitoria Spain; e Division of Pre-college and Undergraduate Studies, Brown University Providence RI USA; f GNA School of Pharmacy, GNA University Phagwara Punjab India dineshpotlia123@gmail.com dr.neerajchoudhary@gmail.com; g Symbiosis Medical College for Women & Symbiosis, University Hospital and Research Centre, Symbiosis International (Deemed University) Pune 412115 India ksuresh.babu@smcw.siu.edu.in

## Abstract

A revolutionary shift in the field of nanotheranostics is the development of smart therapeutics guided by 4D nanoimaging, which addresses the shortcomings of traditional platforms that rely on static imaging and passive drug delivery. The spatial and temporal control of these systems can be accurately tuned to modulate dynamics in disease microenvironment assays, especially for complex diseases, such as cancer and inflammatory diseases. Recent developments in stimulus-responsive nanomaterials and real-time visualization modalities have enabled more specific and less damaging adaptive, feedback-based therapeutic interventions. This review presents an overview of the recent literature on the design, operating principles, and biomedical applications of 4D nanoimaging-guided smart therapeutics. Particular attention is paid to building nanoplatforms that are responsive to endogenous and exogenous signals and advanced imaging tools (*e.g.*, near-infrared fluorescence and photoacoustic imaging), as well as integrating them with artificial intelligence to establish predictive and closed-loop therapeutic platforms. Applicable literature has been critically reviewed with regards to the performance parameters of spatial–temporal resolution, therapy release performance, and therapeutic effects. The reviewed literature shows how the 4D nanoimaging platforms reach high spatiotemporal precision to allow real-time monitoring of the biodistribution of nanoparticles and disease progression. The controlled drug release efficiencies of the stimuli-responsive systems typically range from 70% to 90%, and the integration of multimodal imaging provides better diagnostic performance and tissue penetration. The closed-loop therapeutic regulation that this platform provides through its integration with AI models and a biosensing interface dramatically enhances nanomedicine targeting specificity and therapeutic indices over conventional approaches. A complete paradigm shift to adaptive, personalized smart therapeutics directed by the 4D nanoimaging system will facilitate the development of real-time, microenvironment-specific interventions. While preclinical data have been encouraging, issues like biocompatibility, scalability, and clinical translation remain. It is expected that the integration of AI-driven analytics in multifunctional nanoplatforms will jumpstart the translation of these technologies into clinical settings.

## Abstract 2

4D nanoimaging integrates time-resolved imaging, smart nanoplatforms and adaptive drug release to track dynamic disease microenvironments, enabling image-guided, feedback-controlled and personalized therapy.

## Graphical abstract

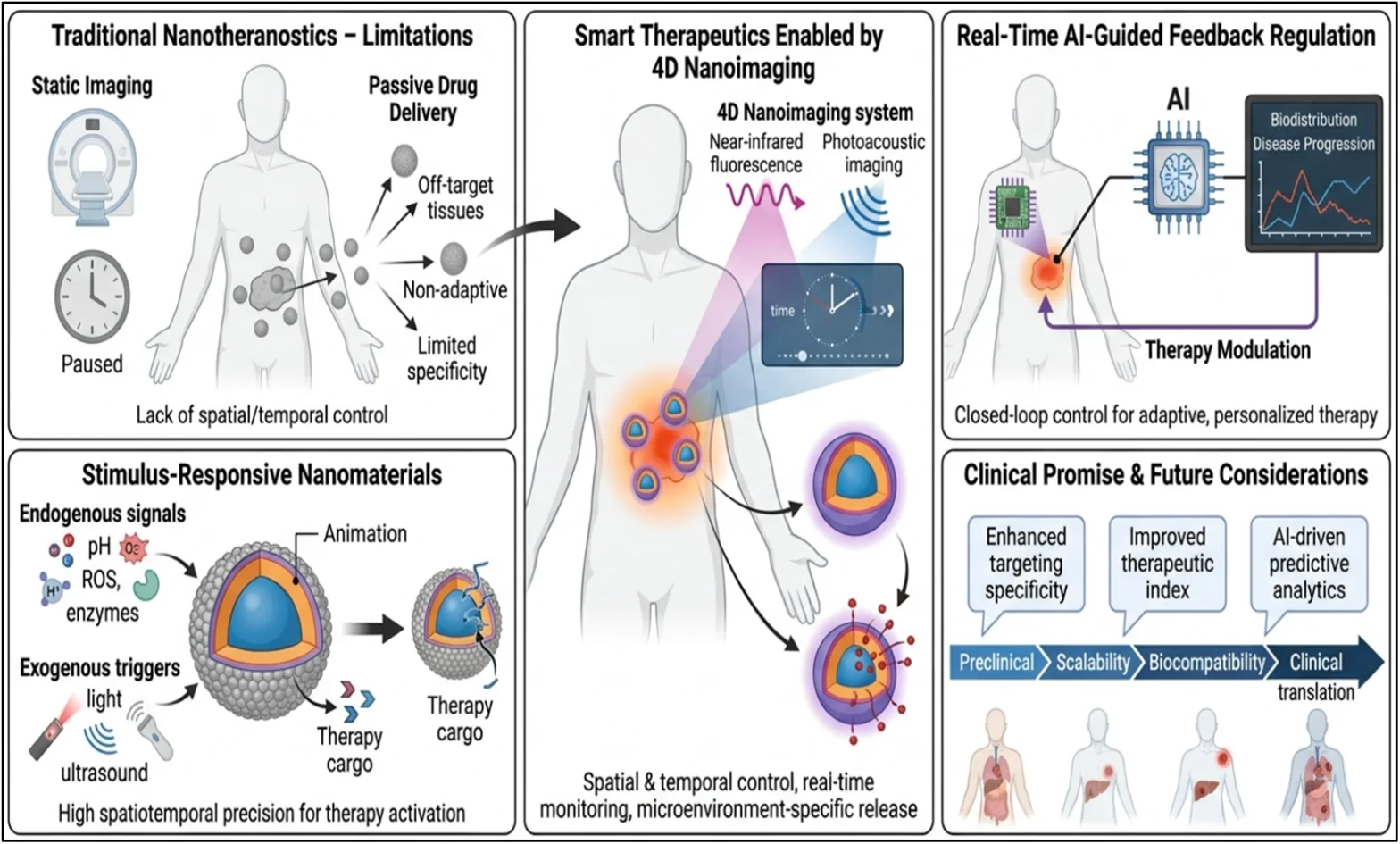

## 1. Introduction

### 1.1 Evolution of nanotheranostics

The field of nanotheranostics is rapidly evolving from conventional nanocarriers that primarily rely on passive accumulation to intelligent 4D platforms capable of dynamically modulating therapeutic delivery and enabling real-time imaging in response to physiological and pathological signals.^1,2^ This represents a paradigm shift that incorporates advanced functionalities, allowing adaptive responses to complicated microenvironmental changes and spatiotemporal variations in disease microenvironments to improve therapeutic efficacy and diagnostic accuracy.^3^ Real-time nanoparticles capable of responding to inherent biological cues or external signals constitute a breakthrough in this area and enable exact control of drug delivery for the targeted treatment of cancer.^4,5^ These intelligent nanomaterials are intended to respond to particular endogenous stimuli, including pH, temperature, or enzyme activity, or exogenous stimuli so as to allow for highly localized and targeted therapies.^6^ Such a creative solution makes it possible to deliver the drug with the highest possible accuracy in terms of its spatial and temporal control, which has a huge effect on the specificity of the therapy and decreases the number of off-target effects due to the peculiarities of the pathological microenvironment.^7,8^ This kind of development is vital for surmounting the shortcomings of traditional oncology, where systemic toxicity and insufficient targeting tend to negatively affect the effectiveness of the treatment.^9,10^ When combined with other forms of cancer care and with the latest forms of biomedical imaging, these smart drug delivery systems could play a crucial role in improving therapeutic precision and efficacy in most modalities.^11,12^ Such a complex interaction of diagnostic imaging and responsive therapeutic delivery is the key focus of the new phenomenon that is 4D nanoimaging-directed smart therapeutics.

### 1.2 Conceptual framework of 4D nanoimaging-guided smart therapeutics

This paradigm hypothesizes the combination of real-time, high-resolution imaging modalities and stimuli-responsive nanoplatforms to control therapeutic interventions in complex disease microenvironments dynamically and in a closed loop. This synergetic concept aims to overcome the limitations of traditional therapies, in which individual medicines will be realized through the adaptive delivery of drugs and monitoring of the therapeutic response.^13^ The developed imaging techniques, like 4D nanoimaging, can offer improved spatiotemporal resolution in the diagnosis and treatment of most pathologies, hence maximizing the treatment effects and reducing the negative effects on normal tissues.^14,15^ This makes it easy to integrate diagnostic and therapeutic capabilities, a strategy known as theranostics, to allow for the detection and treatment of disease by the smart control of nanocarrier behavior in response to dynamic biological signals.^16,17^ This approach can be integrated to make therapeutic strategies adapt in a real-time fashion to continuously deliver feedback about the disease microenvironment to optimize the effectiveness and reduce off-target events.^18^ Such dynamic interplay between imaging and therapeutic action allows for a precision medicine approach in which the best dose and regimen of medication can be identified and refined in real-time, thus preventing under-treatment and over-treatment.^19^ This advanced control system is of utmost importance in responding to the heterogeneity of disease conditions, especially in oncology, where accurate drug delivery to the tumor location is extremely important in enhancing patient outcomes.^20,21^ The integration of diagnostics and therapy on the same platform contributes to a significant advancement of individual treatment outcomes through the possibility of viewing disorders in a comprehensive way, both at the molecular and tissue scales.^22^ This combined method enables adaptive therapeutic policies that can be optimally adjusted to the responses and disease course of the individual patient, resulting in the most therapeutic benefit and the least adverse effects.^23^

### 1.3 Clinical need for spatiotemporal control in disease microenvironments

Due to the heterogeneous and dynamic nature of disease microenvironments, especially in diseases such as cancer, therapeutic interventions have to be precisely spatiotemporally controlled to maximize efficacy and minimize off-target toxicity.^24,25^ This type of control enables the selective transport and release of therapeutic agents directly into the site of pathology, in many cases in reaction to disease-specific markers or externally provided stimuli, hence changing the passive therapies into active, smart therapies.^26,27^ Such an ability is essential in maximizing drug concentration at the target site and, at the same time, decreases systemic exposure, which is a major problem in traditional pharmacotherapy.^28^ Also, by utilizing the controlled physiological parameters of the diseased microenvironment, *e.g.*, pH, hypoxia, or enzymatic activity, these intelligent platforms can allow for the release of therapeutic agents to be delivered in a location and at a time most appropriate.^29^ Such accuracy is essential in theranostic studies, in which diagnostic imaging is used to control and track the local therapeutic response, thus enabling real-time evaluation of therapeutic responses and individualized corrections.^30,31^ The purpose of this tailored therapy is to address the shortcomings of conventional methods of cancer treatment, thereby maximizing the administration of drugs to specific tumor sites and minimizing the systemic toxicity that is usually caused by drug treatments.^32^ This is very important considering that ordinary anti-cancer medications may not be tumor-specific, thus causing severe side effects, including damage to healthy tissues^33^ (Table 1).

**Table 1:** Evolution, conceptual framework, and clinical need of 4D nanoimaging-guided smart therapeutics

Sections	Key theme	Core concepts	Technological advancements	Clinical significance	Ref.
Evolution of nanotheranostics	Transition from static to dynamic systems	Shift from passive nanocarriers to adaptive, stimuli-responsive platforms enabling real-time modulation of drug delivery	Smart nanoparticles responsive to endogenous (pH, enzymes, temperature) and exogenous stimuli; integration with imaging systems	Enhanced targeting, reduced systemic toxicity, improved therapeutic precision in oncology	1, 4, 7 and 12
Conceptual framework of 4D nanoimaging-guided smart therapeutics	Integration of imaging and therapy (theranostics)	Closed-loop systems combining real-time imaging with responsive drug delivery; adaptive therapeutic modulation	4D nanoimaging enabling spatiotemporal tracking; intelligent nanoplatforms for feedback-controlled therapy	Personalized medicine through real-time treatment optimization; improved efficacy and minimized off-target effects	13, 16, 19, 22 and 23
Clinical need for spatiotemporal control	Addressing disease microenvironment heterogeneity	Importance of precise spatial and temporal control in therapeutic delivery and activation	Stimuli-triggered drug release based on tumor microenvironment (hypoxia, pH, enzymes); image-guided therapy	Maximized drug accumulation at target sites, reduced toxicity, improved outcomes in cancer therapy	24, 26, 30, 32 and 33

### 1.4 Scope, objectives, and structure of the review

This review critically looks at the fundamental principles and the state-of-the-art developments in 4D nanoimaging-directed intelligent therapeutics, including an outline of the complex design parameters of stimuli-responsive nanoplatforms and how they are combined with advanced imaging technologies. It critically discusses the engineering principles that regulate the responsiveness of nanocarriers to endogenous (*e.g.*, pH, redox potential, and enzymatic activity) and exogenous stimuli (*e.g.*, light, magnetic fields, and ultrasound). It also discusses how these responsive systems can be used to achieve accurate spatiotemporal release of drugs and therapies and to monitor therapeutic responses in a dynamic disease microenvironment. The review will further explore the clinical implications and future outlook of these combined platforms, highlighting their possible revolutionary effect on precision medicine due to their ability to improve diagnostic services and focused treatment procedures.

Though several excellent reviews have summarized advances in nanoimaging, theranostic nanomedicine, stimuli-responsive drug delivery, and artificial intelligence-assisted nanotechnology, most have focused on individual technological components rather than their functional integration. Reviews dedicated to nanoimaging primarily emphasize imaging modalities and contrast agents, whereas theranostic reviews largely concentrate on combining diagnosis and therapy. Similarly, reviews of stimuli-responsive nanomedicine generally classify nanocarriers according to endogenous or exogenous triggers, and AI-focused reviews mainly address computational analysis or image interpretation. In contrast, the present review adopts a systems-level perspective by integrating dynamic 4D nanoimaging, programmable stimuli-responsive nanoplatforms, adaptive nanostructures, artificial intelligence, and feedback-controlled therapeutic regulation within a single conceptual framework. This integrated perspective not only summarizes recent technological advances but also critically evaluates their convergence toward next-generation precision nanomedicine and highlights key challenges for clinical translation (Table 2).

**Table 2:** Comparison of the present review with recent representative reviews

Recent review	Primary focus	Key limitations	Present review contribution
Nanoimaging reviews	Imaging modalities and contrast agents	Limited discussion of therapeutic integration	Integrates imaging with programmable therapeutic regulation
Theranostic nanomedicine reviews	Combined diagnosis and therapy	Limited emphasis on dynamic imaging and temporal monitoring	Focuses on real-time/4D nanoimaging-guided therapeutic adaptation
Stimuli-responsive nanomedicine reviews	Endogenous and exogenous trigger mechanisms	Primarily material-centered; limited systems-level integration	Compares stimuli-responsive systems and places them within adaptive therapeutic ecosystems
AI in nanomedicine reviews	Image analysis, prediction, and computational models	Limited discussion of programmable nanocarriers and imaging-guided therapy	Integrates AI with multimodal imaging, adaptive nanoplatforms, and feedback-controlled drug delivery
Multimodal imaging reviews	Integration of imaging modalities	Limited discussion of stimuli-responsive drug delivery and intelligent nanomaterials	Discusses multimodal imaging as one component of adaptive therapeutic systems rather than an isolated technology
Present review	4D nanoimaging-guided smart therapeutics	Provides an integrated framework spanning imaging, nanomaterials, AI, adaptive feedback, and clinical translation	Introduces a unified conceptual model linking dynamic nanoimaging, programmable nanostructures, intelligent therapeutic control, disease-specific applications, and translational challenges

## 2. Disease microenvironments as dynamic therapeutic targets

### 2.1 Spatiotemporal heterogeneity in the tumor microenvironment (TME)

The TME comprises a complex and dynamic structure of cellular and acellular components, including cancer cells, stromal cells, immune cells, extracellular matrix, and an abnormal vasculature that causes tremendous spatial and temporal alterations in pathophysiological parameters.^34^ Such dynamic properties as changes in pH, hypoxia, and redox potentials have a significant impact on tumor development, metastatic capacity, and responsiveness to therapy.^35,36^ The inherent heterogeneity demands the development of more complicated stimuli-responsive nanomedicines that are capable of reconfiguring their responses to these diverse pathological cues, consequently enhancing targeted delivery and therapeutic effect.^37,38^ One such system is internal reservoir-responsive nanocarriers, such as pH-responsive, redox gradient, and enzyme activity-responsive devices, which offer improved control over drug delivery at the tumor bed, minimizing the undesirable off-target effects and maximizing the internalization of the therapy.^39,40^ These systems have been designed to change their physicochemical properties, such as size, charge, or conformation, in response to certain microenvironmental cues, allowing them to deliver drugs on-demand and accumulate more effectively in pathological areas.^41–44^ This selective release ensures that the release of drug payloads is accurately focused at the site of pathology, optimizing therapeutic effects and minimizing systemic toxicity.^45,46^ This tactic takes advantage of the existing contrast between normal and pathological tissues and provides a way to achieve highly targeted and effective therapy.^47^ In addition, novel intelligent nanoplatforms combine diagnostics and therapy, using internal and external stimuli, such as pH, temperature, and light, to enable targeted drug delivery, real-time imaging, and activated therapeutic activity.^48^ This complex interaction of elements in the tumor microenvironment poses an obstinate obstacle to traditional cancer therapies, which affect the penetration and effectiveness of drugs.^49,50^

### 2.2 Inflammatory and immune microenvironment dynamics

Massive spatiotemporal heterogeneity is also present in the immune microenvironment, encompassing various immune cell populations and secreted factors, and it has a profound impact on tumor immune evasion and responsiveness to immunotherapies.^51^ This time-varying pattern of immune cell inflammation and cytokine gradients determines the effectiveness of immunomodulatory nanotherapies aimed at restructuring the immunosuppressive niche or boosting the capacity of immune effector cells.^52,53^ Temporal coordination of immune responses and their spatial localization to the tumor, therefore, is an important factor to consider in the rational design of nanocarriers able to respond flexibly to changing immune conditions and achieve the delivery of immunomodulatory reagents with high spatiotemporal fidelity.^54,55^ Such adaptive nanocarriers need to overcome the complex cellular interactions and biochemical gradients to regulate immune checkpoints locally, induce immunogenic cell death, or activate T-cells, surmounting the resistance mechanisms intrinsic to the immune suppressive tumor microenvironment. Endogenous endowments, such as acidosis and high levels of glutathione in the tumor, are used to release controlled drug release of nanomedicines and, thus, targeted therapy to a specific tumor environment in a precise manner.^56^

### 2.3 4D nanostructure and material design of spatiotemporal control

Therapeutic agent budgeting within an intricate and complex disease microenvironment requires the ability to precisely control the spatiotemporal distribution of therapeutic agents through the design of nanostructures with physicochemical properties that are precisely tuned to respond to pathophysiological signals. This is achieved through the development of nanoplatforms that are capable of detecting and responding to endogenous action, including pH, redox potential, and enzymatic activity, or exogenous stimulation, including light and magnetic fields, to obtain on-demand drug delivery and targeted therapeutic modulation.^57,58^

### 2.4 Microenvironmental features: pH, hypoxia, redox gradients, and enzymatic activity

The non-homogeneous dispersion and dynamic variability of these biophysical and biochemical parameters in disease microenvironments, especially in tumors, are important indicators and represent highly selective targets in the rational formulation of smart nanotherapeutics.^59,60^ These phenomena, such as acidosis and high glutathione levels, can be used as endogenous triggers in order to be the subject of specific pathological environment therapeutic intervention.^61^ Examples include pH-responsive nanocarriers that take advantage of the acidic tumor milieu to deliver payloads and redox-responsive systems that can exploit the high levels of glutathione in the intracellular environment to release a drug.^62^ Likewise, nanoplatforms that respond to enzymes take advantage of the existing aberrantly expressed or overexpressed enzymes in diseased tissues to promote the localized delivery and activation of drugs.^63^ The resulting highly selective and effective nanomedicines can be developed through this strategic use of intrinsic microenvironmental cues to decrease systemic toxicity and increase therapeutic efficacy.^64^ In particular, the low pH values of tumors (around 6.5) in comparison with those of normal tissues (pH 7.2–7.4) create a differential stimulus in pH-sensitive drug-delivery systems.^65,66^ On the other hand, the increased levels of reactive oxygen species and glutathione in tumor cells, an outcome of the increased oxidative stress, offer the prospect of designing redox-responsive nanoarchitectures that degrade exclusively under these reductive intracellular conditions.^67^ This method allows superior control over the delivery of the therapeutic payload in the pathological site, hence increasing drug concentration and decreasing off-target effects.^68^ Another approach to selectively release therapeutic agents is the activation of enzyme-controlled platforms by the overexpression of tumor microenvironment-specific enzymes, such as cathepsin B and matrix metalloproteinase-2.^69^

### 2.5 Temporal evolution of disease states and therapeutic windows

The temporal dynamics of disease in general and the dynamic nature of the microenvironment and therapeutic windows in particular are the most critical factors in optimizing the timing and efficacy of 4D nanotherapeutics. Such a temporal insight can guide the development of multi-responsive nanocarriers that can modify their drug-release kinetics in response to changing biochemical recognitions and cellular behaviors during disease progression.^70^ This entails detailed disease trajectory modeling to forecast the best intervention timepoints and program corresponding nanotherapeutic interventions. The resulting microenvironmental sensitivity allows one to create adaptive nanoplatforms capable of modulating drug release kinetics and targeting in real-time in response to changes in the microenvironment, thus optimizing therapeutic efficacy and reducing toxicity. Such a dynamic targeting method, where drug delivery is done with precise spatial and temporal control, greatly enhances drug selectivity and minimizes off-target effects.^71,72^ Such timely control over drug delivery is essential to the therapy of transient disease states or particular cellular activity states that are most prone to therapeutic intervention. To track the dynamic changes in the microenvironment and optimize therapeutic delivery, real-time monitoring features must be introduced to 4D nanoimaging systems.^73^ Such a real-time imaging-therapy feedback response is important to enhance the performance of stimuli-responsive nanocarriers, especially to detect enzyme concentrations or low pH in the disease microenvironment.^74,75^ Thus, endogenously responsive nanocarriers constitute a promising area; these carriers are frequently overexpressed in the tumor microenvironment and can be used to achieve highly specific and localized drug delivery.^76,77^ These enzyme-triggered platforms can be programmed to particularly degrade or release their cargo at high enzymatic activity, indicating pathological conditions, which reduces systemic exposure and improves therapeutic index.^78^ This will enable precise spatiotemporal control of drug release with resultant improved therapeutic effect and decreased systemic toxicity, particularly in chemotherapy.^79,80^ Such systems can be optimized further, *e.g.*, with hierarchical targeting systems, where dynamically modulated physicochemical properties, *e.g.*, size and charge to the surface, are added to enhance accumulation and penetration through various biological barriers.^81^

Although the tumor microenvironment has served as the primary model for developing stimuli-responsive nanomedicine, similar pathological microenvironments are present in several non-oncological diseases. Chronic inflammatory disorders, fibrotic diseases, cardiovascular diseases, and neurodegenerative disorders exhibit characteristic biochemical and physiological alterations that can be exploited for targeted imaging and smart therapeutic delivery.^72^ Common features include elevated reactive oxygen species, abnormal enzyme activity, dysregulated cytokine production, extracellular matrix remodeling, hypoxia, and altered vascular permeability, all of which provide opportunities for microenvironment-responsive nanoplatforms. In inflammatory diseases, such as rheumatoid arthritis and inflammatory bowel disease, excessive production of pro-inflammatory cytokines, oxidative stress, acidic extracellular pH, and increased vascular permeability facilitate selective accumulation and activation of responsive nanocarriers.^73^ Similarly, fibrotic disorders are characterized by excessive extracellular matrix deposition, persistent inflammation, transforming growth factor-β signaling, and matrix metalloproteinase dysregulation, providing molecular targets for enzyme-responsive and matrix-sensitive nanotherapeutic systems.^73^

Cardiovascular diseases, particularly atherosclerosis and myocardial infarction, exhibit localized oxidative stress, inflammatory macrophage infiltration, endothelial dysfunction, and protease activation that can be exploited for targeted molecular imaging and site-specific drug delivery.^74^ In neurodegenerative disorders, including Alzheimer's and Parkinson's diseases, chronic neuroinflammation, oxidative injury, protein aggregation, and blood–brain barrier dysfunction create disease-specific microenvironments that support the development of targeted nanoplatforms for both diagnostic imaging and therapeutic intervention.^76^ These examples demonstrate that the principles underlying microenvironment-responsive nanoimaging are broadly applicable across multiple disease settings, although the dominant biological triggers and therapeutic objectives vary according to disease pathology.^76^

Consequently, the concept of disease microenvironment-responsive nanomedicine should be regarded as a general precision medicine strategy rather than one limited to oncology. By exploiting disease-specific biochemical and physiological signatures, smart nanoplatforms can enable targeted imaging, controlled therapeutic activation, and dynamic monitoring across a broad spectrum of human diseases.

### 2.6 Quantitative parameters governing microenvironment modulation

To accurately tune disease microenvironments, a quantitative description of the most significant parameters, such as interstitial fluid pressure, nutrient gradients, and immune cell infiltration dynamics, is needed. The quantitative interactions of these parameters are important in designing nanotherapeutics that can act within the complex biophysical environment of diseased tissues, which eventually affect therapeutic responses. Sophisticated bioimaging technologies are required to accurately map these parameters and forecast how they will respond to therapeutic interventions.^82^ Monitoring of these microenvironmental factors, such as temperature and pH, can be realized in real-time by the incorporation of photoluminescent nanothermometers or pH-sensing probes into nanocomposite systems, enabling continuous evaluation of therapeutic activity.^83^ Such quantitative knowledge can be used to create finely adjusted nanomaterials, which can display particular and environmentally sensitive activities, to potentially maximize therapeutic actions.^84,85^ This does not exclude the specific manipulation of enzyme-sensitive nanocarriers, whose rates of degradation and the curve of drug release could be highly adjusted on the basis of the increased enzyme activity of diseased tissues.^86^ The rationale behind this is that the notable enzymatic overexpression in pathological locations, including the approximately 6-fold upsurge in matrix metalloproteinase-2 in MDA-MB-231 cells or approximately 8-fold higher hyaluronidase in high-grade bladder cancer than in normal tissues, can be utilized in order to deliver drugs locally.^87^ This exclusive enzymatic targeting limits off-target effects and greatly improves the therapeutic index by focusing drug action in the areas of greatest need. In addition to enzyme-based approaches, other microenvironmentally sensed signals, including variations in pH or redox potential, can be used to achieve controlled drug delivery, with more advanced smart nanoplatforms emerging. Such advanced platforms may also incorporate stimuli-responsive features to tune properties such as size, charge or surface properties to circumvent spatial barriers within the complex tumor microenvironment, including a dense extracellular matrix or high interstitial fluid pressure, which frequently impede therapeutic delivery.^88^ An example is nanoparticles that have been designed to degrade the components of the extracellular matrix, including collagen or hyaluronan, to create nanoparticles with a greater ability to penetrate solid tumors^89^ (Fig. 1).

**Fig. 1:**
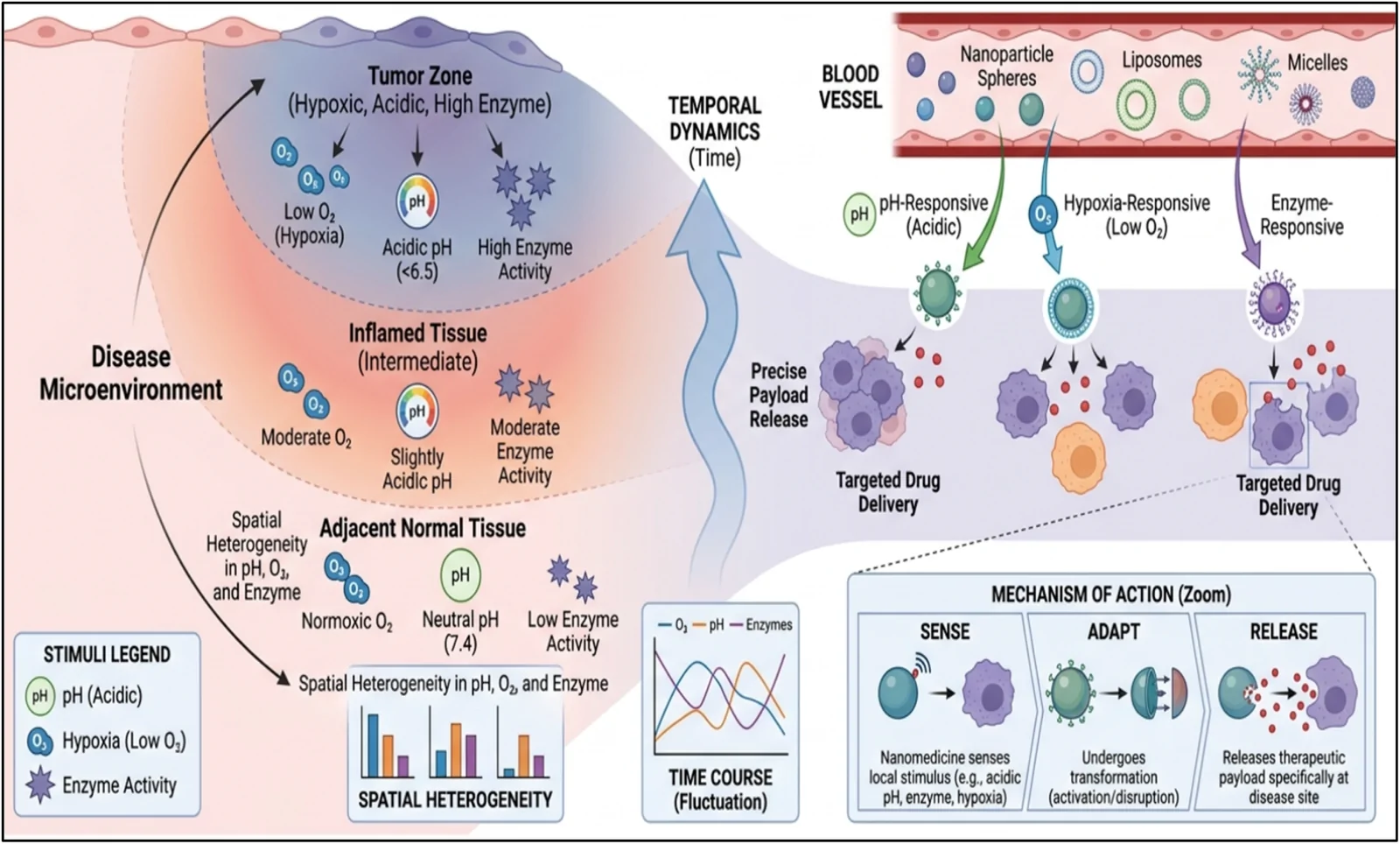
Disease microenvironment as a dynamic therapeutic target: leveraging spatiotemporal heterogeneity with stimuli- responsive nanomedicines.

The disease microenvironment is characterized by measurable physicochemical changes that serve as important design criteria for stimuli-responsive nanoplatforms. For example, the extracellular pH of most healthy tissues is tightly maintained at approximately 7.35–7.45, whereas the extracellular pH of solid tumors typically ranges from 6.5 to 6.9, with endosomal and lysosomal compartments exhibiting even lower pH values of 5.5–6.0 and 4.5–5.5, respectively.^83,84^ Similarly, the interstitial fluid pressure in normal tissues is generally in the 0–3 mmHg range, whereas many solid tumors exhibit elevated pressures ranging from 10 to 40 mmHg, with values exceeding 50 mmHg reported in aggressive malignancies.^85^ Tumor hypoxia is also reflected by oxygen partial pressures frequently below 10 mmHg, compared with the approximately 40–60 mmHg values in normal tissues. In addition, matrix metalloproteinases (particularly MMP-2 and MMP-9), hyaluronidase, and cathepsins are often overexpressed in diseased tissues, while intracellular glutathione concentrations may reach 2–10 mM, substantially higher than the extracellular levels (2–20 µM), providing important triggers for redox-responsive nanotherapeutics.^86,88^

## 3. Fundamentals of 4D nanoimaging

### 3.1 Definition and principles of 4D imaging (3D + time dimension)

In imaging science, 4D imaging encompasses the acquisition of volumetric (three-dimensional) image datasets over time, thereby enabling visualization of structural or functional changes as a dynamic sequence (3D + time).^90^ Classic examples include respiratory-gated 4D computed tomography, cardiac 4D MRI, dynamic PET, and volumetric ultrasound imaging, where successive three-dimensional datasets are reconstructed to characterize continuously evolving biological processes.^91^ It encompasses temporal resolution, dynamic quantitative imaging, and spatiotemporal molecular mapping, allowing continuous characterization of biological processes rather than isolated observations.

It is important to note that the concept of 4D imaging adopted in this review is consistent with internationally accepted terminology in medical imaging, where the fourth dimension represents the temporal evolution of three-dimensional (3D) volumetric datasets acquired over time.^92^ This established framework has been widely applied in modalities, such as 4D computed tomography (4D CT), 4D magnetic resonance imaging (4D MRI), and dynamic positron emission tomography (PET). In the present review, the term 4D nanoimaging refers to the application of this well-established spatiotemporal imaging paradigm to nanoparticle-enabled molecular imaging and image-guided smart therapeutics, rather than introducing a new definition of 4D imaging.^91^

Within nanomedicine, the concept of 4D nanoimaging extends this framework toward dynamic visualization of nanoparticle transport, biodistribution, target accumulation, therapeutic activation, and clearance.^92^ Importantly, however, not every imaging modality used in nanotheranostics inherently provides true volumetric time-resolved imaging. Some techniques primarily enable longitudinal monitoring or real-time molecular visualization, whereas others support genuine 3D + time acquisition through tomographical reconstruction.^93^

Temporal resolution is a defining characteristic of 4D nanoimaging because it determines the ability to capture rapid biological events, such as nanoparticle transport, vascular perfusion, cellular uptake, drug release, and therapeutic response. High temporal resolution enables repeated volumetric image acquisition over short time intervals, providing continuous insight into dynamic physiological processes that cannot be adequately represented by single static images.^93^ Accordingly, throughout this review, the term 4D nanoimaging is used to describe imaging platforms capable of monitoring the spatial distribution and temporal evolution of nanoparticle behavior when supported by volumetric reconstruction, tomographical imaging, or dynamic multimodal acquisition. Modalities lacking volumetric acquisition are discussed as dynamic or longitudinal imaging techniques rather than intrinsic 4D systems.^94^

In addition, 4D nanoimaging facilitates dynamic quantitative imaging, in which imaging-derived parameters—including nanoparticle concentration, pharmacokinetics, tissue perfusion, receptor expression, and therapeutic response—are measured continuously or sequentially over time. These quantitative datasets provide objective biomarkers for monitoring treatment efficacy and optimizing therapeutic interventions.^90^

Another defining feature is spatiotemporal molecular mapping, which simultaneously characterizes the spatial distribution and temporal evolution of molecular and cellular events within living tissues. Rather than simply identifying the location of nanoparticles, 4D nanoimaging enables visualization of dynamic interactions between nanotherapeutics and the tumor microenvironment, including changes in vascular permeability, enzyme activity, pH, hypoxia, immune-cell infiltration, and drug activation.^91^

Importantly, 4D nanoimaging differs fundamentally from conventional longitudinal imaging. Longitudinal imaging typically consists of discrete imaging examinations performed at widely separated time points (*e.g.*, before treatment, during therapy, and after treatment), providing snapshots of disease progression.^92^ In contrast, 4D nanoimaging captures continuous or high-frequency volumetric datasets that reveal the dynamic evolution of biological processes in real time or near real time. Consequently, 4D nanoimaging not only monitors structural changes but also enables quantitative assessment of evolving molecular events, supporting adaptive therapeutic decision-making and feedback-controlled smart nanotherapeutic systems.^93^

### 3.2 Imaging resolution metrics: spatial, temporal, and sensitivity limits

These parameters determine the accuracy and sensitivity of imaging devices, which have a direct impact on the identification of nanoscale objects and changes in the body over time. Spatial resolution is defined as the smallest feature that can be resolved; temporal resolution represents the capability of observing dynamic processes, and sensitivity is the minimum signal that can be detected.^95^ These parameters are essential for the optimization of subcellular nanoparticle interactions and real-time pharmacokinetic phenomena that tend to take place at high velocities and low concentrations in complicated biological fluids.^96^ This necessitates the research and development of new sophisticated microscopies, such as super-resolution, to beat the diffraction limit and institute sub-cellular observations of nanomedicine dynamics.^97^ Nanoparticles can now be studied at the cellular scale in intact whole organisms using new tissue-clearing methods that overcome the limitations of traditional imaging methods, including insufficient resolution or poor systemic applicability.^98^ Such time-course analyses of high-resolution images provide a more complete perspective of nanoparticle endocytosis and intracellular trafficking, which is paramount in enhancing its targeting approaches in nanomedical research.^99^ This increased spatial and temporal resolution can also be used to gain better insights into the effects of design parameters of nanomaterials on their behavior *in vivo*, particularly their uptake by specific endocytotic pathways and their interaction with particular subsets of myeloid cells.^100^ Future studies will explore a combination of these high-resolution imaging modalities with improved light source analysis techniques, so that a comprehensive, label-free analysis of transport and transformation of nanomedicine *in vivo* can be achieved.^101,102^ Nanotechnology is also critical in improving the diagnostic approach by improving the resolution, sensitivity and specificity of various imaging modalities, like CT, MRI, PET, ultrasound and optical imaging systems.^103^ These enhancements allow one to identify diseases earlier and more accurately monitor the effects of treatment, which is beyond the range of traditional imaging technology.^104,105^

As summarized in Table 3, no single imaging modality simultaneously provides optimal spatial resolution, penetration depth, temporal resolution, and molecular sensitivity. Optical imaging techniques offer excellent temporal resolution and molecular sensitivity but are limited by imaging depth. Conversely, MRI, CT, PET, and SPECT provide deep-tissue visualization and established clinical utility, although each technique features trade-offs in spatial resolution, acquisition time, sensitivity, radiation exposure, or cost. These complementary characteristics have driven the development of multimodal imaging strategies that combine the strengths of individual modalities for comprehensive spatiotemporal characterization and image-guided smart therapeutics.

**Table 3:** Quantitative comparison of imaging modalities used in 4D nanoimaging

Imaging modality	Spatial resolution	Penetration depth	Temporal resolution	Molecular sensitivity	Clinical maturity	Major limitation
NIR-I fluorescence	10–100 µm	5–10 mm	Real-time (ms–s)	High	Preclinical/early clinical	Limited tissue penetration
NIR-II fluorescence	10–50 µm	10–30 mm	Real-time (ms–s)	High	Early clinical	Limited approved fluorophores
Photoacoustic imaging	50–300 µm	20–50 mm	ms–s	Moderate–High	Early clinical	Depth-dependent signal attenuation
MRI	50–500 µm	Whole body	Seconds–minutes	Moderate	Established clinical	Low molecular sensitivity
CT	50–500 µm	Whole body	Seconds	Low	Established clinical	Ionizing radiation
PET	4–6 mm	Whole body	Minutes	Very high (pM–nM)	Established clinical	Limited spatial resolution
SPECT	7–10 mm	Whole body	Minutes	High (nM)	Established clinical	Lower resolution than PET
Ultrasound	100–500 µm	Several cm	Real-time (ms)	Low	Established clinical	Operator dependency
Multimodal imaging	Modality-dependent	Variable	Variable	High	Emerging	Increased complexity and cost

As summarized in Table 4, each imaging modality provides distinct strengths with respect to spatial resolution, temporal resolution, imaging depth, and molecular sensitivity. Optical techniques, such as NIR-I, NIR-II, and photoacoustic imaging, offer excellent temporal resolution for dynamic imaging but are limited by tissue penetration. Conversely, MRI, CT, PET, and SPECT provide deeper tissue visualization, although with varying trade-offs in spatial resolution, molecular sensitivity, acquisition time, radiation exposure, and cost. Consequently, multimodal imaging approaches are increasingly being explored to integrate the complementary advantages of individual modalities for comprehensive spatiotemporal characterization and image-guided smart therapeutics.

**Table 4:** Comparative resolution metrics of imaging modalities used in 4D nanoimaging

Imaging modality	Spatial resolution	Temporal resolution	Imaging depth	Molecular sensitivity	Major strength	Principal limitation
NIR-I fluorescence	High (µm–mm)	High (real-time)	Low (≈5–10 mm)	High	Rapid optical imaging with high sensitivity	Limited tissue penetration and autofluorescence
NIR-II fluorescence	High (≈10–100 µm)	High (real-time)	Moderate (up to several cm)	High	Improved penetration and reduced scattering	Limited clinically approved fluorophores
Photoacoustic imaging	High (≈50–300 µm)	Moderate–High	Moderate (≈2–5 cm)	Moderate	Combines optical contrast with ultrasound resolution	Limited penetration in deep tissues
MRI	High (≈50–500 µm)	Moderate	Whole body	Moderate	Excellent soft-tissue contrast and anatomical detail	Lower molecular sensitivity and longer acquisition time
CT	Very high (≈50–500 µm)	High	Whole body	Low	High spatial resolution and rapid acquisition	Ionizing radiation and limited molecular information
PET	Moderate (≈4–6 mm)	Moderate	Whole body	Very high	Highly sensitive molecular imaging	Radiation exposure and relatively low spatial resolution
SPECT	Moderate (≈7–10 mm)	Moderate	Whole body	High	Functional imaging with established tracers	Lower spatial resolution than PET
Multimodal imaging	Variable	Variable	Variable	High	Integrates complementary anatomical and molecular information	Increased system complexity and cost

### 3.3 Real-time tracking of nanoparticle biodistribution and kinetics

Real-time monitoring is critical in the explanation of the complex pharmacokinetics and pharmacodynamics of nanomedicines since it provides critical data about their absorption, distribution, metabolism and excretion properties. This approach helps researchers to optimize the delivery of drugs by making sure that therapeutic agents reach their target sites and are effective within the desired duration.^106^ Their versatile ability to monitor the localization and interactions of nanoparticles *in vivo* with target cells or tissues offers unprecedented insights into targeting specificity and off-target accumulation, which are paramount for assessing therapeutic efficacy and potential toxicity.^107,108^ Radiotracer-based methods, for example, can be used to conduct longitudinal nanoparticle fate research, which is essential and can give important insights into accumulation, distribution, and clearance kinetics with time.^109^ Positron emission tomography and single-photon emission computed tomography have outstanding sensitivity, making them useful for tracking radiolabeled nanoparticles. However, due to their inherent spatial resolution, they often require co-registration with computed tomography or magnetic resonance imaging to accurately locate the tissue anatomy.^110^ This multimodal method plays a vital part in the correlation of the behavior of functional nanoparticles to high-resolution anatomical contexts, especially when it comes to analyzing their distribution in complicated biological systems.^111^ These developments in real-time monitoring, augmented with nanotechnology, have not only increased the accuracy of the diagnosis, but also enhanced the imaging of drug delivery and potential toxicity, maximizing drug release research under controlled conditions.^112,113^ Moreover, real-time imaging, combined with state-of-the-art nanoplatforms, can be adopted to provide complex feedback control, enabling on-demand modulation of therapeutic interventions according to monitored biodistribution and localized drug release dynamics. This approach enables therapeutic delivery to be adaptively modulated to optimize dosage and timing, achieving the desired effect and reducing systemic side effects. The application of pharmascintigraphy *via* gamma scintigraphy, SPECT and PET is vital for the noninvasive, real-time monitoring of the biodistribution, pharmacokinetics, and therapeutic efficacy of nanomedicines. This methodology offers information necessary to optimize formulations and ensure accurate targeting.^114,115^ In addition, the union of radiopharmaceutical science and nanotechnology has facilitated the creation of nanoparticles that serve as advanced vectors for the targeted delivery of therapeutic and diagnostic cargo across biomagnetic terrains, with exceptional precision^116^ (Table 5).

**Table 5:** Comparative performance of major imaging modalities used in 4D nanoimaging

Performance parameter	MRI	PET	CT	NIR fluorescence	Photoacoustic imaging	OCT	Ultrasound	Ref.
Spatial resolution	50–500 µm	4–6 mm	50–500 µm	10–100 µm	50–300 µm	1–15 µm	100–500 µm	95, 97 and 100
Penetration depth	Whole body	Whole body	Whole body	5–30 mm	20–50 mm	1–3 mm	Several cm	95–98
Temporal resolution	Seconds–minutes	Minutes	Seconds	Real-time (ms–s)	Real-time (ms–s)	Real-time	Real-time	95 and 103–105
Molecular sensitivity	Moderate	Very high	Low	High	Moderate–High	Low	Low	101 and 102
Ionizing radiation	No	Yes	Yes	No	No	No	No	106–111, 114 and 115
Contrast requirement	Often required	Radiotracer required	Often required	Fluorophore required	Optical absorber required	Usually not required	Contrast optional	106–109, 112 and 113
Clinical maturity	Established	Established	Established	Early clinical	Early clinical	Established (selected applications)	Established	107–111
Major strength	Excellent soft-tissue contrast	Highest molecular sensitivity	Excellent anatomical detail	High sensitivity and real-time imaging	Functional and structural imaging	Micrometer-scale resolution	Portable, inexpensive, real-time	112 and 113
Major limitation	Long acquisition time, lower sensitivity	Radiation, high cost	Radiation, limited molecular information	Limited tissue penetration	Limited imaging depth	Limited penetration depth	Operator dependence	114–116

Despite their exceptional molecular sensitivity, radiotracer-based imaging modalities have several practical limitations that influence their widespread clinical application. PET and SPECT involve exposure to ionizing radiation, requiring careful optimization of administered activity to balance diagnostic accuracy with patient safety.^114^ Furthermore, the short half-lives of many clinically used radionuclides, such as ^18^F (approximately 110 min), ^11^C (approximately 20 min), and ^68^Ga (approximately 68 min), necessitate specialized production facilities and efficient radiopharmaceutical distribution. In addition, PET and SPECT imaging require dedicated instrumentation, radiochemistry infrastructure, and trained personnel, contributing to relatively high operational costs and limited accessibility in many healthcare settings. Hybrid imaging systems and advances in radiotracer development continue to improve clinical utility; however, further optimization of cost-effectiveness, standardized imaging protocols, and broader availability will be essential to facilitate wider clinical adoption.^115,116^Table 5 highlights the complementary performance characteristics of the major imaging modalities used in 4D nanoimaging. No single modality simultaneously achieves optimal spatial resolution, penetration depth, molecular sensitivity, and temporal resolution. Consequently, multimodal imaging strategies are increasingly employed to combine the strengths of individual techniques for comprehensive spatiotemporal imaging and image-guided smart therapeutics.

### 3.4 Data acquisition, reconstruction, and quantification strategies

Certain strategies are critical to the transformation of raw imaging signals into meaningful biological data, such as advanced image processing algorithms, noise rejection, and quantification of the concentration and distribution of nanoparticles in complex physiological environments.^117,118^ Such strategies entail the use of advanced computational techniques to achieve accurate complex signal deconvolution, tissue attenuation correction, and radiolabeled agent or fluorescent probe localization.^119^ Moreover, artificial intelligence and machine learning algorithms continue to play a key role in automating nanoparticle tracking systems, making high-resolution images, improving data analysis, and providing accurate quantification of nanoparticle properties and distributions.^120^ This is one of the significant advancements that increase diagnostic imaging accuracy by exploiting the sensitivity and specificity of new contrast agents.^121^ High-level algorithms can be used to isolate quantitative values of the uptake rates, retention time, and clearance of nanoparticles, which are required to comprehend their actions *in vivo* and maximize their therapeutic benefits.^122^ This improved quantification is essential for capturing the spatiotemporal dynamics of nanomedicines, providing a holistic perspective of their reactions in disease microenvironments.^22^ Further, the powerful quantification of nanoparticle properties in these microenvironments contributes to predicting their therapeutic effects and informs the development of next-generation nanomedicines with enhanced targeting specificity and release characteristics.^123^ Adjacent frontiers of data science, such as machine learning and AI, may provide chances to increase the efficiency and precision of image creation and data analysis.^124^ Such AI-based solutions may be involved in predictive diagnosis and adaptive therapy, wherein custom treatment programs dynamically adapt to the responses of individual patients and disease development.^125^ Such implementations of AI and machine learning on data analysis pipelines can greatly simplify the interpretation of large datasets produced by 4D nanoimaging and detect the existence of subtle patterns and correlations that would otherwise remain unnoticed when using traditional data-analysis techniques.^126,127^ In particular, deep learning architectures, including convolutional neural networks and recurrent neural networks, can be trained on large datasets of past data and be used to make predictions regarding how nanoparticles will behave under different physiological conditions, which will optimize the design of more robust and effective nanotherapeutic formulations^128^ (Fig. 2 and Table 6).

**Fig. 2:**
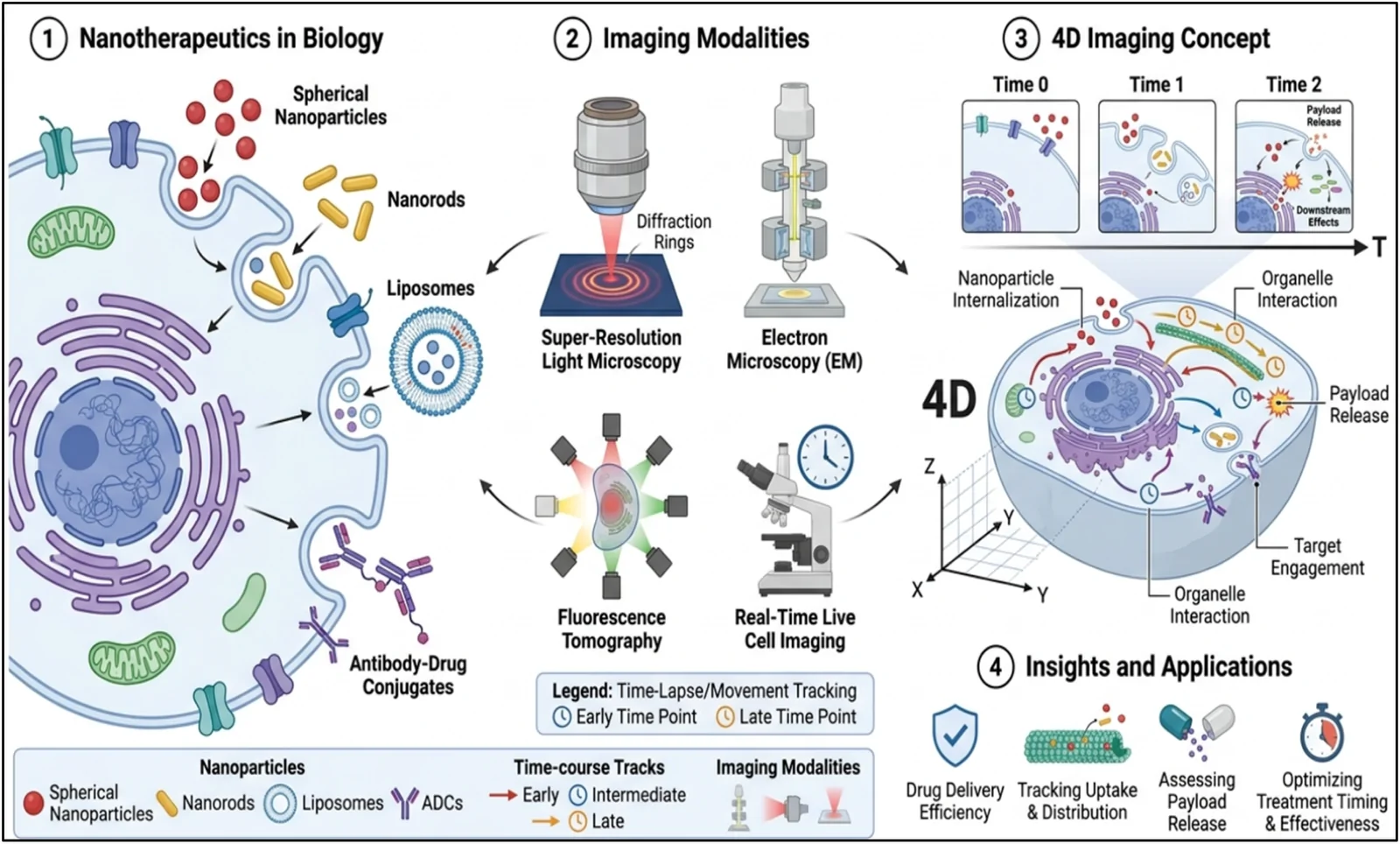
Fundamentals of 4D nanoimaging for nanotherapeutics.

**Table 6:** Imaging modalities according to their 4D capability

Imaging modality	True 3D acquisition	Temporal imaging	Genuine 4D capability	Typical application
NIR planar fluorescence	No	Yes	No	Longitudinal imaging
Fluorescence molecular tomography	Yes	Yes	Yes	Dynamic biodistribution
Photoacoustic microscopy	Limited	Yes	Partial	Functional imaging
3D Photoacoustic tomography	Yes	Yes	Yes	Dynamic vascular imaging
PET	Yes	Yes	Yes	Dynamic pharmacokinetics
PET/CT	Yes	Yes	Yes	Whole-body nanoparticle tracking
MRI	Yes	Yes	Yes	Dynamic contrast imaging
CT	Yes	Yes	Yes	Dynamic perfusion

Thus, 4D nanoimaging should be regarded not merely as three-dimensional imaging acquired over time, but as a dynamic imaging paradigm that integrates high-temporal-resolution volumetric acquisition with quantitative molecular mapping to enable adaptive monitoring of biological processes and image-guided smart therapeutics.

### 3.5 Emerging imaging modalities in nanoimaging

In addition to established imaging modalities, several emerging optical and hybrid imaging techniques are attracting increasing attention because of their ability to provide complementary structural, molecular, and functional information for precision nanomedicine. Although most remain at the preclinical or early translational stage, these technologies may significantly enhance future 4D nanoimaging-guided therapeutic strategies.^115^

Raman imaging, particularly surface-enhanced Raman scattering (SERS), offers highly specific molecular fingerprinting with excellent sensitivity and minimal photobleaching. Functionalized nanoparticles can substantially amplify Raman signals, enabling targeted molecular imaging and multiplexed detection of disease biomarkers. However, relatively weak intrinsic Raman scattering, limited tissue penetration, and the need for specialized instrumentation continue to restrict widespread clinical implementation.^115,116^

Hyperspectral imaging (HSI) simultaneously acquires spatial and spectral information, allowing the discrimination of tissues based on their unique spectral signatures. When combined with targeted nanoparticles, HSI enables improved tissue characterization, biomarker identification, and intraoperative guidance without ionizing radiation. Nevertheless, large data volumes, prolonged image-processing times, and limited penetration depth remain important challenges for routine clinical applications.^117^

Cerenkov luminescence imaging (CLI) exploits optical photons generated during radioactive decay and provides an opportunity to combine radionuclide imaging with high-sensitivity optical visualization. CLI has shown promise for image-guided surgery and molecular imaging using clinically established PET radionuclides. However, signal attenuation in deep tissues and relatively low photon yield currently limit its broader clinical utility.^118^

X-ray luminescence imaging (XLI) combines the deep tissue penetration of X-rays with optical emission from scintillating nanoparticles, enabling high-resolution molecular imaging at greater imaging depths than conventional fluorescence techniques. This approach has demonstrated encouraging results in preclinical image-guided therapeutic applications, although further optimization of scintillator materials, imaging sensitivity, radiation dose, and clinical instrumentation is required before widespread translation.^118,119^

Collectively, these emerging imaging technologies complement established modalities by providing additional molecular specificity, spectral information, and multimodal imaging capabilities. Their integration with smart nanoplatforms and artificial intelligence may further expand the capabilities of next-generation nanoimaging-guided precision therapeutics.

## 4. Advanced nanoimaging modalities for real-time guidance

### 4.1 Near-infrared (NIR-I and NIR-II) fluorescence imaging

This modality builds upon the low autofluorescence and deep tissue penetration properties of near-infrared light to provide high-resolution, real-time imaging of nanoplatform biodistribution and molecular processes in biological systems. NIR-I (700–900 nm) and NIR-II (1000–1700 nm) windows have some unique benefits: with respect to the signal-to-noise ratio, as well as the penetration depth, this technology enables non-invasive tracing of therapeutic agent delivery and real-time evaluation of disease progression.^11^ In particular, NIR-II imaging provides real-time longitudinal imaging, which uses longer wavelengths (1000–1700 nm) and greatly reduces the scattering of photons and tissue autofluorescence, resulting in improved spatial resolution and penetration depths than NIR-I.^2^ This allows easy visualization of nanoparticles in deep tissues with enhanced signal quality and allows for the precise tracking of theranostic agents in complex biological environments.^129^ NIR fluorescence, due to its non-invasive properties, makes longitudinal studies possible on longer time scales, which are necessary in the process of establishing the dynamic correlation between nanotherapeutics and disease microenvironments. This particularly applies in measuring the pharmacokinetic and biodistribution of the nanoparticles in real time, thereby maximizing therapeutic delivery strategies.^130^ Besides, machine learning algorithms could enhance the processing and analysis of NIR fluorescence data, enabling the accurate determination of nanoparticle accumulation and therapeutic effects in cells and at a subcellular level.^5,131^ Auto-detection of the smallest morphological and molecular expression changes within the tissue is easy by this process and is vital for the early detection of disease and the customized adjustment of treatment options.^132^ The high signal-to-noise ratio, enhanced resolution, and deep penetration of NIR-II imaging provide a window for real-time, longitudinal imaging, which is useful particularly when resolving elaborate vascular architecture and tumor margins in deep tissues.^33^ Each of these capabilities makes NIR-II imaging an attractive methodology to inform complicated surgeries and accurately ascertain therapeutic effects in anatomically inaccessible locations. The development of new NIR-II imaging techniques that include real-time longitudinal imaging fluorophores and nanoparticle-based probes^133,134^ is key for the further improvement of sensitivity and specificity for this imaging modality. Conventional NIR fluorescence imaging is primarily a two-dimensional optical imaging technique and, therefore, should not automatically be regarded as a true 4D modality. Its principal strength lies in longitudinal monitoring of nanoparticle accumulation and therapeutic responses.^131^ Nevertheless, when combined with fluorescence molecular tomography (FMT), diffuse optical tomography, or light-sheet volumetric reconstruction, NIR fluorescence can generate sequential three-dimensional datasets, enabling genuine 4D visualization of nanoparticle biodistribution.^131,132^

Despite its excellent sensitivity and reduced tissue autofluorescence, NIR-II imaging still faces several challenges that limit its widespread clinical implementation. Its penetration depth remains insufficient for imaging deeply located organs compared with nuclear imaging techniques, and many NIR-II fluorophores require further optimization regarding long-term biocompatibility, pharmacokinetics, and biodegradability. In addition, standardized imaging protocols and clinically approved contrast agents remain limited, restricting routine clinical adoption. Future research should focus on improving fluorophore safety, quantitative imaging accuracy, and large-scale clinical validation.^132^

### 4.2 Photoacoustic imaging: mechanisms and deep-tissue applications

Photoacoustic imaging takes advantage of the photoacoustic effect, whereby the energy stored in ultraviolet light is converted into ultrasonic waves, and the latter are measured to create high-resolution images with depths surpassing those of other optical imaging techniques.^135,136^ It is an intermediate approach that combines the high optical contrast of a fluorescence system with the high spatial resolution and deep tissue penetration of ultrasound, and thus, it is most appropriate in visualizing nanoparticles in a complex biological system.^25^ This combination opens the possibility of non-invasive, real-time imaging of nanoparticle localization and functional indices, like oxygen saturation, in deep tissues, which are essential to drug delivery and treatment. The convergence of photoacoustic imaging and novel nanotechnology has facilitated the use of innovative theranostic platforms to both image and treat tumors, particularly in the field of oncology where the localization of tumors and the monitoring of therapeutic effects are extremely important. In particular, nanoparticles in photoacoustics, including gold nanoparticles, can be used as contrasting agents by converting the light energy absorbed into acoustic signals that can be used to provide high-resolution images of biological samples and deep tissues.^137^ Multimodal imaging can be achieved with the tunable absorption characteristics of several photoacoustic agents, which can give a better picture of the physiological microenvironment.^138^ Photoacoustic imaging, particularly NIR-II imaging that provides real-time longitudinal imaging, has high potential to be used clinically because it has a superior ability to penetrate tissue compared to other fluorescence-based techniques.^139^ This imaging modality has been further improved by the development of NIR-II photoacoustic imaging, which decreases background interference and increases sensitivity, spatial resolution and depth of tissue penetration of the X-rays.^140^ High-resolution is especially helpful in imaging fast biological events and outlining the edges of tumors with greater detail.^141^ In photoacoustic imaging, pulses of laser light are introduced to a specimen, and the constituent molecules absorb the light energy, producing thermoelastic expansion that results in measurable acoustic waves.^142^ Modern matrix-array photoacoustic tomography permits the acquisition of volumetric-reconstruction data repeatedly over time, enabling dynamic visualization of vascular remodelling, oxygen saturation, and nanoparticle accumulation. Such implementations represent genuine 4D photoacoustic imaging, whereas conventional two-dimensional photoacoustic imaging should instead be regarded as dynamic functional imaging.^142^ Although photoacoustic imaging combines high optical contrast with ultrasonic resolution, several technical limitations remain. Image quality may be influenced by tissue heterogeneity, optical attenuation, and acoustic scattering, particularly in deep tissues. The availability of standardized clinical instrumentation and reproducible quantitative analysis also remains limited. Consequently, further technological refinement and multicenter validation studies are required before widespread clinical implementation can be achieved.^142^

### 4.3 Multimodal imaging platforms (fluorescence-PA-MRI-CT integration)

In multimodal platforms, pressure waves are captured by an ultrasound transducer, and thereafter, detailed images depicting light absorption characteristics in the tissue can be reconstructed.^28^ Photoacoustic imaging can be combined with other modalities, which include positron emission tomography, to enjoy the advantages of high-resolution anatomical imaging and molecular specificity.^143^ This synergistic approach gives a description of the biological mechanism in detail by correlating the anatomy to the molecular pathways, thereby enhancing the accuracy of diagnosis and therapeutic-treatment recommendation. This multimodal approach offers powerful and quantitative data to assess the efficiency of treatment and the state of the disease in real-time to address the limitations of single-modal approaches. These systems tend to exploit the latest developments in laser technology, multispectral algorithms and inversion methods to boost the quality of the signal being acquired and deliver serviceable data, *e.g.*, blood oxygenation and metabolic activity data.^144,145^ Photoacoustic imaging can also be easily translated to clinical practice since it is inherently compatible with other traditional ultrasound systems, which enables one to gather structural and functional data of target tissues in real-time.^146^ This allows the physiological events and therapeutic feedback to be monitored in real time, which is needed in personalized medicine approaches.^147,148^ Multimodal imaging should not be considered synonymous with 4D imaging. Rather, multimodal systems combine complementary structural and functional information acquired from different imaging techniques. When one or more constituent modalities perform repeated volumetric acquisition over time (for example, dynamic PET/CT or PET/MRI), the integrated platform may support 4D characterization of nanoparticle behavior. However, simple co-registration of independently acquired datasets does not itself constitute 4D imaging.^148^ All of these high multimodal imaging services and high-performance computational workflows can enable predictive analytics, whereby clinicians can predict the outcome of the disease and can tailor therapeutic protocols to the unique biological response. These predictive features are crucial to precision medicine, whereby nanotherapy dose and delivery time can be actively altered to achieve highest efficacy and minimize side effects.^51^ PET and SPECT provide highly sensitive molecular imaging but involve exposure to ionizing radiation, dependence on short-lived radioisotopes, and relatively high operational costs. The requirement for specialized radiochemistry facilities and cyclotron infrastructure limits accessibility in many healthcare settings. Moreover, the integration of multifunctional nanoplatforms with clinically approved radiotracers requires extensive regulatory evaluation before routine clinical use.^148^

### 4.4 Signal amplification and noise reduction strategies

The continued creation of new contrast agents, new reconstruction methods, and machine learning strategies is important to improve signal-to-noise values and reduce artifacts in 4D nanoimaging data. These are solutions that are essential to improve the quantitative validity and clinical utility of these imaging techniques.^149^ Developing sophisticated hardware, such as optimized detectors and light sources, and improving data-acquisition protocols also help in increasing the overall performance of these complex imaging systems.^150^ These strategies are also essential for the smooth linkage of multimodal imaging systems and the development of theranostic nanocarriers to enable real-time tracking of drug and therapeutic responses.^51^ Newer methods of computed tomography, including quantitative photoacoustic tomography (qPAT) and AI-assisted image processing, are being used more and more to achieve high-quality estimates of chromophore concentrations and overall image quality with reduced speckle noise and better segmentation.^151^ These calculation methods, combined with the new technology of transducers and new beamforming methods, can further improve the spatial and temporal resolutions of photoacoustic imaging, which can be used to better visualize complex biological systems and processes.

#### 4.4.1 AI-assisted image reconstruction

Artificial intelligence has significantly improved image reconstruction, segmentation, and quantitative analysis; however, its clinical implementation remains constrained by limited external validation, algorithmic bias, dataset heterogeneity, and reduced interpretability of complex deep learning models. Differences in imaging protocols across institutions may further affect model generalizability. Establishing standardized datasets, transparent reporting guidelines, and prospective multicenter validation studies will be essential for reliable clinical deployment.^150^

#### 4.4.2 Smart nanoplatforms

Multifunctional smart nanoplatforms represent a promising strategy for precision medicine but also introduce considerable challenges. Increasing structural complexity may complicate large-scale synthesis, quality control, and batch-to-batch reproducibility. Furthermore, long-term toxicity, biodegradation, immunogenicity, and manufacturing costs remain insufficiently characterized for many advanced nanomaterials. Addressing these issues will be critical for successful regulatory approval and commercial translation.^151^

#### 4.4.3 Stimuli-responsive nanomedicine

Although stimuli-responsive nanocarriers enable controlled drug release and improved targeting, biological heterogeneity among patients may reduce the predictability of endogenous triggers, such as pH, redox status, or enzyme expression. Similarly, externally activated systems often require specialized equipment and careful optimization of treatment parameters. Hybrid multi-stimuli platforms may improve therapeutic precision but also increase formulation complexity and regulatory challenges.^149^

### 4.5 Comparative performance analysis of imaging modalities

To define the strengths and weaknesses of different imaging modalities in terms of spatial and temporal resolution, depth of penetration and sensitivity, it is important to make an extensive comparative analysis of different imaging modalities and thus help in choosing the best imaging modality among various applications in biomedical studies.^152^ Indicatively, whereas photoacoustic tomography provides better penetration depth and structural data, it can be outperformed by modalities such as fluorescence microscopy in terms of time resolution in superficial imaging tasks, requiring consideration of the research question and target tissue.^151,153^ This work-intensive analysis also considers other factors, such as signal-to-noise ratio, motion artifact sensitivity, and exogenous contrast agent requirement, which, in joint consideration, affect the choice of the most appropriate imaging model to utilize in dynamic nanoscale processes.^154^ This comparative analysis also takes into account the clinical translational potential, cost-effectiveness, and regulatory issues of both imaging methods with the aim of closing the gap between experimental innovation and practical implementation.^155,156^ In the future, the combination of new nanomaterials with advanced molecular imaging promises to provide real-time measurements and adaptive interventions, thus optimizing treatment effectiveness and reducing side effects^157^ (Fig. 3).

**Fig. 3:**
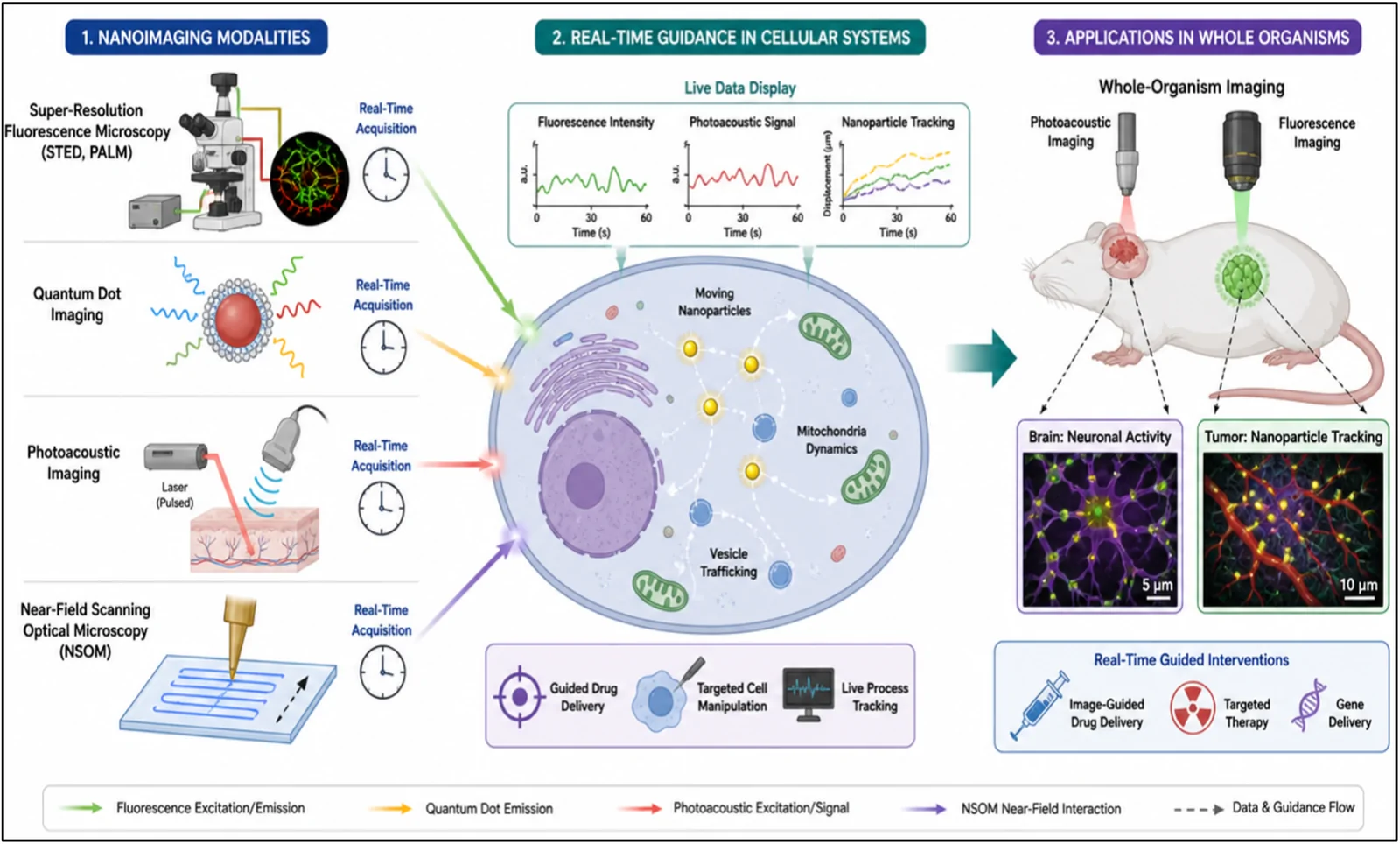
Advanced nanoimaging modalities for real-time guidance in biological systems.

## 5. Design of stimuli-responsive smart nanoplatforms

Stimuli-responsive nanoplatforms have emerged as an important strategy for improving the precision of image-guided therapeutics by enabling site-specific activation in response to endogenous or externally applied stimuli. Unlike conventional nanocarriers, these systems are engineered to respond selectively to pathological cues, such as acidic pH, reactive oxygen species (ROS), enzymes, hypoxia, or externally applied triggers, including light, ultrasound, magnetic fields, and temperature.^158^ Although all stimuli-responsive platforms share the common objective of achieving controlled therapeutic activation while minimizing off-target effects, each approach employs distinct activation mechanisms that influence nanoparticle design, imaging performance, therapeutic efficacy, and clinical applicability. The following sections summarize the unique characteristics, advantages, and current limitations of the major classes of stimuli-responsive nanoplatforms.^159^

### 5.1 Physicochemical design principles (size, shape, and surface engineering)

Smart nanoplatforms have physicochemical properties, such as size, shape, surface charge, and material composition, that provide fundamental control over their interaction with bio-systems, bio-distribution and cellular uptake processes. These parameters play a crucial role when it comes to establishing the *in vivo* functionality of nanocarriers, as well as in determining whether they can react to certain endogenous or exogenous stimuli in a controlled and accurate way.^6^ The rational design of these nanoplatforms also incorporates the optimization of drug loading capacity, release kinetics, and long-term stability that are crucial in achieving the desired therapeutic outcomes.^3^ Further, intelligently choosing material elements, including polymers, lipids, or inorganic nanoparticles, has a direct effect on the biocompatibility, biodegradability, and immunogenicity of the nanoplatforms, impacting the final biomedical applicability of the nanoplatforms. Techniques for functionalizing the surface, including conjugation of targeting ligands or stealth coats, are significant in enhancing specificity and prolonging circulation, additionally maximizing the delivery of drugs to disease sites.^10^ Smart responsive nanoplatforms, in turn, often comprise elements that can respond to a plethora of endogenous and exogenous stimuli, allowing the initiation of therapeutic effects conveniently and the fine-tuning of activation.^48,64^ This requires a variety of microenvironmental stimuli, such as pH, redox potential, and enzyme activity, to induce drug release or activation, transferring passive delivery to active, targeted therapy.^17,158^ These adaptive nanocarrier therapeutic payloads are released in response to particular triggers in the target microenvironment, improving therapeutic selectivity.^62^

### 5.2 Endogenous stimuli-responsive systems

These are designed to take advantage of distinctive physiological states common in disease microenvironments, including aberrant pH, high redox potential, or certain overexpression, and are carefully engineered to control exactly the delivery of therapeutic agents.^40,71^ This selectivity reduces systemic toxicity and increases the efficacy of different therapeutic agents, as it is clear that the drug is released at the pathological location.^159^

#### 5.2.1 pH-responsive nanocarriers

pH-responsive nanoplatforms exploit the acidic extracellular environment of diseased tissues and the lower pH of intracellular endosomes and lysosomes to achieve selective activation.^160^ These systems typically employ acid-labile linkers, ionizable polymers, or pH-sensitive coatings that remain stable under physiological conditions but undergo structural changes in acidic environments, enabling localized drug release and enhanced intracellular delivery.^161^ Such strategies have shown considerable promise for improving therapeutic precision while limiting systemic exposure. Nevertheless, variations in tissue acidity among different disease types and individual patients may influence activation efficiency, highlighting the need for further optimization of pH-responsive designs.^162^ As an example, electrostatically attractive nanoprotherapeutics, such as cationic nanocarriers, can be used to take advantage of the proton sponge effect and endosomal escape in tumor microenvironments that highly express acid-glycasedecanoylactones (AGLs).^81^ Also, the differences in the pH of normal tissues (pH 7.0–7.5), tumor extracellular space (pH 6.5–6.9), and intracellular lysosomes (pH 4.5–5.5) allow the kinetics of drug release to be controlled to a fine degree, reducing off-target effects and increasing therapeutic indices.^65^ It is this specific pH sensitivity that allows the creation of intelligent nanoplatforms that can release their payload in a very controlled fashion with minimal side effects, resulting in the maximum therapeutic effect.^43,73^ Some block copolymers with acid-cleavable bonds or ionizable functional groups are designed to undergo cargo release under the low pH conditions typical of tumor microenvironments.^163^

#### 5.2.2 Redox-responsive systems

Redox-responsive systems exploit the increased intracellular glutathione levels and redox potentials of many cancer cells, through the use of disulfide bonds or other reducible bonds that can be cleaved under such conditions to release therapeutic agents.^164^ This redox disparity between healthy and diseased cells represents an excellent mechanism for achieving selective drug delivery, increased treatment effect, and decreased systemic toxicity.^66^ Dual-responsive nanocarriers that combine pH and redox responsiveness have been designed to increase tumor-specific drug delivery by depleting synergistic releases.^52^ These systems take advantage of the common biochemical properties of the microenvironment of the tumor to provide highly localized activation of drugs, which is of great benefit compared to normal, non-responsive drug delivery methods.^37,42,46^

#### 5.2.3 Enzyme-triggered platforms

These schemes take advantage of the hyperactivation of certain enzymes in pathological microenvironments, such as diverse proteases in tumor tissues, to activate the regulated breakdown of nanocarrier constituents or the breakdown of drug conjugates, releasing the therapeutic cargo.^45,165^ Such enzyme specificity can be used for the extremely localized release of drugs, reducing systemic exposure and improving therapeutic indices by only releasing drugs at active sites when pathological enzyme activity is present.^44^ As an example, intracellularly cleavable linkages, such as enzyme-sensitive linkers, can trigger a stability switch in polymer-conjugated drugs, coordinating subsequent drug release.^166^ Additionally, advanced enzyme-based systems feature cleavage bond techniques, *e.g.*, the scission of boronic acid or polylinkages by H_2_O_2_, which cause nanoparticle and cargo release at the core.^7^ This method takes advantage of the increased concentration of certain enzymes in diseased tissue to narrow the focus of drug delivery and improve targeting specificity.^70^

#### 5.2.4 Comparative perspective on endogenous stimuli

Although pH-, redox-, and enzyme-responsive nanoplatforms are often presented as independent strategies, they differ substantially in biological specificity, spatial precision, and translational feasibility. pH-responsive systems exploit the universal characteristics of the tumor microenvironment but often suffer from relatively small pH gradients between healthy and diseased tissues, limiting specificity.^65^ Redox-responsive systems provide improved intracellular selectivity through elevated glutathione concentrations but may exhibit heterogeneous responses across tumor subtypes. Enzyme-responsive platforms generally achieve the highest biological specificity because they exploit disease-associated enzymatic overexpression; however, their effectiveness depends strongly on interpatient variability and enzyme expression profiles. Consequently, hybrid nanoplatforms integrating multiple endogenous triggers are increasingly being explored to overcome the limitations associated with individual stimuli and improve therapeutic robustness.^165^

#### 5.2.5 ROS-responsive nanoplatforms

ROS-responsive nanoplatforms utilize the elevated oxidative stress characteristic of many pathological conditions as an endogenous trigger for therapeutic activation. Oxidation-sensitive linkers, thioketal-containing polymers, and ROS-cleavable nanocarriers enable selective drug release within oxidative microenvironments while supporting molecular imaging of disease activity.^163^ These systems are particularly attractive for disorders associated with chronic inflammation and cancer; however, variability in ROS levels across tissues and disease stages may affect therapeutic consistency and requires careful optimization during nanoplatform design.^165^

Collectively, stimuli-responsive nanoplatforms provide versatile strategies for achieving image-guided precision therapeutics by exploiting endogenous pathological signals or externally applied physical triggers. Although all approaches aim to improve therapeutic selectivity through controlled activation, they differ in their activation mechanisms, disease specificity, imaging compatibility, and translational readiness. Future development should focus on integrating complementary stimuli, improving manufacturing reproducibility, validating long-term safety, and establishing standardized protocols to facilitate successful clinical translation.

### 5.3 Exogenous stimuli-responsive systems

Unlike endogenous systems, exogenous stimulus-responsive systems are driven by physical external stimuli, *e.g.*, light, magnetic fields, or ultrasound, and external forces are used to regulate drug release kinetics and spatial localization. This extrinsic control is most beneficial in breaking through constraints imposed by endogenous stimuli, including off-target release and early degradation in circulation.^39^ Further, they are externally modulated, providing increased spatiotemporal control and enabling clinicians to accurately specify when and where therapeutic agents are released, a feature especially useful in the case of deep-seated tumors or dynamic disease conditions.^167^

#### 5.3.1 NIR/light-triggered nanotherapeutics

These systems exploit the capabilities of near-infrared light to pass through biological tissues with minimal scattering and absorption and activate drug release or therapeutic effects at a specific site and at a certain distance, without the need to be invasive.^87^ This feature enables accurate spatiotemporal control of drug delivery, with incident light being able to generate localized heating of photothermal agents to cause the release of encapsulated therapeutics.^168^ In other cases, the photoisomerization or photocleavage reactions in the nanocarrier can be directly used to induce the dissociation of the payload upon specific light exposure.^169^ Photosensitive linkers can experience conformational changes using particular wavelengths, which cause nanostructures to disintegrate and release drugs on command.^170^ This controlled external delivery enables the on-demand delivery of drugs, improving therapeutic efficiency in areas where traditional delivery modalities do not reach and reducing side effects in the system. Beyond that, gene delivery systems that are light-responsive can be precisely targeted using external light sources, allowing the release of nucleic acids in a highly localized and temporally specific manner, which is essential when using gene therapy in a targeted fashion.^77,171^

#### 5.3.2 Magnetic, ultrasound, and thermal activation

These systems exploit external physical forces to generate therapeutic effects or cause drug release to provide very precise control over spatiotemporal delivery. The magnetic nanoparticles can be directed to the areas of interest (targets) by remote magnetic fields, and localized drug delivery can be achieved *via* hyperthermia or mechanical operation (magnetic particles).^72^ Likewise, focused ultrasound may trigger cavitation or heating and cause cell membrane permeabilization or targeted drug release of heat-sensitive nanocarriers.^84^ The concept of thermal activation is based on the use of polymers or lipid-phase transitions that are sensitive to temperature, and it commonly involves the use of photothermal or magnetic hyperthermia to release encapsulated cargo at a predetermined thermal target rapidly.^80,172^ Furthermore, photothermal agents like gold nanorods may be capable of converting NIR radiation into thermal energy, thus releasing payloads of temperature-sensitive nanocarriers.^173^ The benefits of such a multi-modal strategy of photoactivation and other external stimuli, like NIR radiation, include the development of advanced systems of delivery with greater accuracy and effectiveness.^174,175^ Sophisticated theranostic applications are also enabled with the help of this integrated strategy, so that the therapeutic responses could be monitored in real time, along with triggered drug delivery.^23^

#### 5.3.3 . 4D nanostructure design to control spatiotemporal dynamics

The design of nanostructures forms the basis for attaining spatiotemporal control of therapeutic intervention. This is through the design of materials that have certain physicochemical features that allow dynamic responsiveness to endogenous physiological stimuli or exogenous physical stimuli and hence control drug release kinetics and targeting accuracy.^176^

#### 5.3.4 Comparative evaluation of exogenous triggers

External stimuli offer superior temporal control over drug activation compared with endogenous triggers because therapeutic initiation is determined by the operator rather than the biological microenvironment. However, each modality presents distinct engineering constraints.^168^ Near-infrared activation provides excellent spatial precision but limited penetration depth. Ultrasound achieves deeper tissue penetration but may produce heterogeneous acoustic exposure in irregular tumors. Magnetic field-guided systems enable remote localization yet require specialized instrumentation and often demonstrate relatively modest targeting efficiency in deep tissues.^174^ Consequently, no single external stimulus currently satisfies all clinical requirements, supporting the growing interest in multimodal activation strategies that combine complementary physical triggers.^176^

### 5.4 Triggered drug release kinetics and efficiency optimization

In this section, a closer examination of the quantitative aspects that dictate the rate and extent of the liberation of therapeutic agents by smart nanoplatforms and how the therapeutic effect can be optimized to minimize off-target effects is performed. Careful considerations of the degradation rates of nanocarriers, limits of stimuli-response, and encapsulation efficiencies of the payload can be adopted as optimization measures to suit the requirements of the therapeutic agent, in terms of either pharmacodynamics or pharmacokinetics.^177^ This can be done by modifying the physicochemical properties of the nanocarriers to give the required release profiles, which can be burst release to trigger immediate therapy intervention or sustained release to produce a long-term effect. Advanced kinetic modelling and *in vivo*/*in vitro* testing are required to make estimates and optimize the behavior of drug release under complex biological scenarios, thereby achieving immense control of therapeutic levels at the target site.^178^ Diffusion barriers in the disease microenvironment frequently limit the efficacy of triggered drug release *in vivo*, and enhanced nanocarrier designs have been created to circumvent these obstacles by active transport or permeability enhancement. Such a high degree of optimization is required in the process of translating these advanced nanoplatforms into clinical therapies, especially those that require a high degree of dose control and focal therapy.^179^ The most important considerations in this optimization are drug loading and release kinetics, both of which must be tuned to achieve therapeutic levels without releasing excessive amounts of nanoparticles. These parameters are governed by the size, porosity and composition of nanoparticles.^58,180^ The rate of release of therapeutic agents from such matrices must be carefully tuned, and the required release profile must be achieved by balancing various parameters, including diffusion rates, degradation rates, and responsiveness to stimuli.^181^ As an example, drug elution can be fine-tuned by changing the polymer backbone or incorporating linkers that are cleavable, thereby maximizing therapeutic windows and minimizing systemic exposure^4,8^ (Table 7).

**Table 7:** Triggered drug release optimization and systemic behavior of smart nanoplatforms

Parameter/focus	Scientific description	Design and technological strategies	Therapeutic and clinical implications	Ref.
Triggered drug release kinetics	Quantitative regulation of drug release rate and extent from stimuli-responsive nanocarriers under physiological conditions	Tuning degradation rates, stimuli thresholds, and physicochemical properties of nanocarriers	Enables precise spatiotemporal drug delivery, improving therapeutic efficacy while minimizing off-target toxicity	177 and 178
Release profile engineering	Control of drug release patterns (burst *vs.* sustained) based on therapeutic requirements	Modulation of nanoparticle composition, porosity, and size; incorporation of cleavable linkers and polymer backbone modifications	Optimizes therapeutic windows and ensures controlled drug exposure at target sites	4, 8, 58, 180 and 181
Drug loading and release efficiency	Balancing encapsulation efficiency with controlled release kinetics to prevent premature leakage	Optimization of nanoparticle architecture and material composition	Ensures adequate drug concentration at target site without systemic loss	58 and 180
Overcoming microenvironment barriers	Addressing diffusion limitations and transport constraints within complex disease microenvironments	Design of nanocarriers enabling active transport, enhanced permeability, and stimuli-triggered penetration	Improves drug penetration and accumulation in tumors or diseased tissues	179
Kinetic modeling and validation	Prediction and validation of drug release behavior in biological systems	Advanced computational modeling, *in vitro*/*in vivo* correlation studies	Facilitates rational design and accelerates clinical translation of nanotherapeutics	178
Biocompatibility	Compatibility of nanocarriers with biological systems to minimize toxicity and immune responses	Surface engineering, material selection, and control of protein corona formation	Reduces immunogenicity and enhances the safety profile of nanomedicines	54, 182 and 183
Stability and circulation	Maintenance of structural integrity and prolonged systemic circulation of nanocarriers	PEGylation, surface modification, and optimization of physicochemical stability	Enhances circulation half-life and improves targeting efficiency	185 and 186
Pharmacokinetics and biodistribution	Evaluation of ADME properties governing systemic behavior of nanomedicines	Engineering size, shape, and surface properties to modulate biodistribution and clearance	Optimizes therapeutic index and reduces off-target accumulation	182 and 184
Size and shape optimization	Influence of nanoparticle geometry on biological interaction and transport	Precise control of nanoparticle size (typically 10–150 nm) and morphology	Improves tissue penetration, avoids rapid clearance, and enhances bioavailability	56, 187 and 188
Material composition and degradation	Impact of nanomaterial type on biodegradability and therapeutic performance	Use of mesoporous silica, polymeric nanoparticles, and biodegradable materials	Ensures controlled degradation, sustained drug release, and safe elimination	189–191

### 5.5 Biocompatibility, stability, and pharmacokinetics

Along with the localized efficacy, the systemic activity of nanocarriers, such as their circulation half-life, biodistribution, and elimination pathways, also contributes to the overall therapeutic use and safety profile. The critical considerations needed to ensure biocompatibility and stability to prevent immune responses and deliver the nanocarrier intact to the desired target are crucial.^182^ This warrants detailed research on the nano-bio interface, cellular uptake, and protein corona formation, which are the determinants of the ultimate fate and efficacy of the nanoplatforms *in vivo*.^183^ Therefore, designed surface properties and material architecture play an important role in minimizing immunogenicity and maximizing therapeutic index.^54^ Also, knowing how the nanocarrier design and physiological barriers (endothelial barriers, immune clearance mechanisms, *etc.*) interact is important to predict and optimize *in vivo* performance.^184^ Also, approaches to enhance stability, such as polyethylene glycol surface modification, can enable the extension of the circulation time and the prevention of premature removal by the reticuloendothelial system.^185^ The degradation kinetics of such nanocarriers can also be tailored to suit the therapeutic regimens by making sure that active messengers are released either for a long period or continuously and also making sure that the body clears them.^186^ The size and shape parameters deserve special attention, as even the nanoparticles in this range (150–300 nm) may damage bioavailability and tissue penetration because of their retention in the mononuclear phagocytic system.^56^ Conversely, larger nanoparticles can experience difficulty in penetrating dense tumor stroma or physiological barriers, highlighting the significance of size control to attain optimal treatment outcomes.^187,188^ The wise selection of the nanomaterial composition, *e.g.*, mesoporous silica or polymeric nanoparticles, is an important factor that defines the kinetics of degradation and biocompatibility profile within complex biological milieus (Fig. 4).^189^

**Fig. 4:**
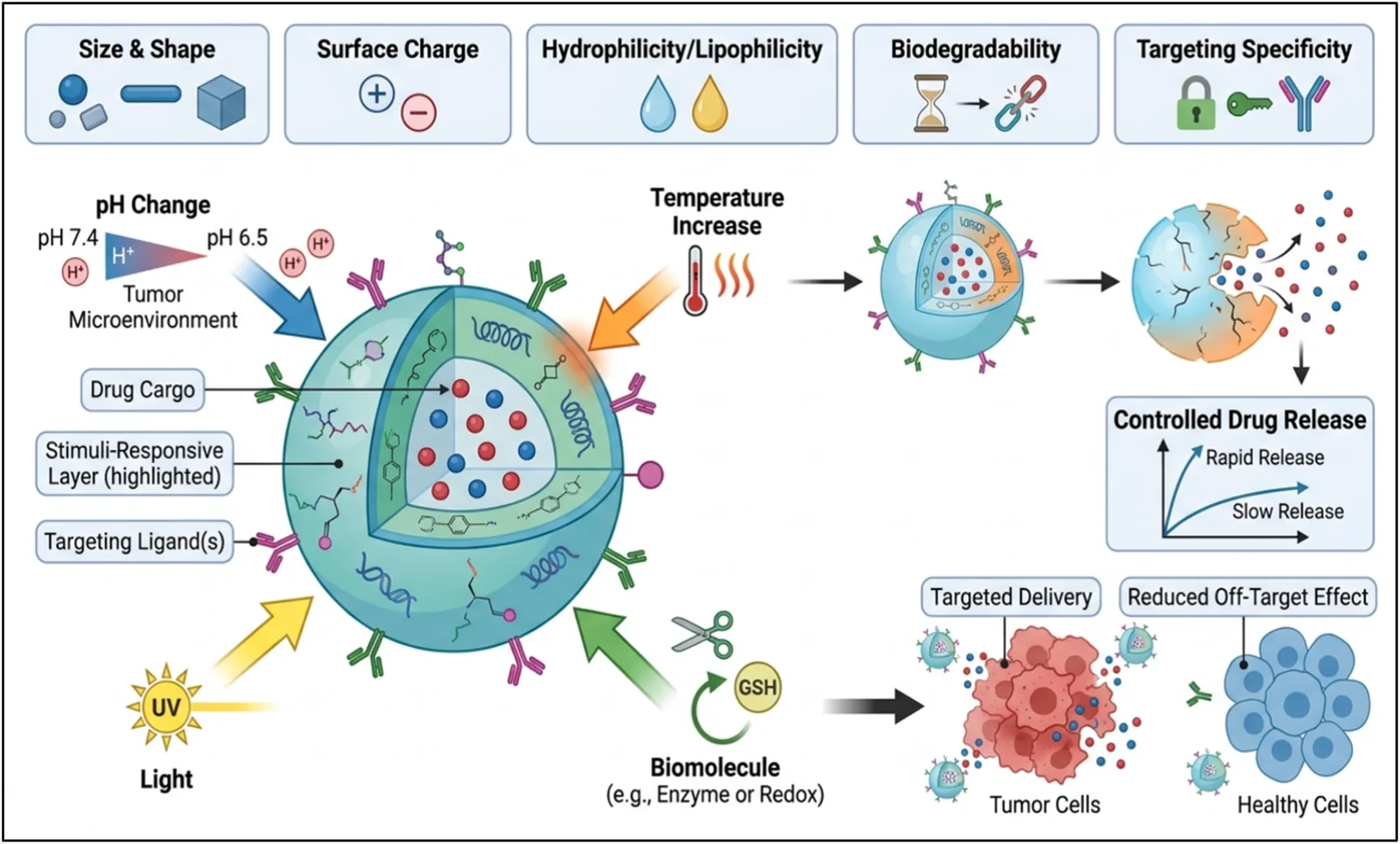
Physicochemical design and stimuli-responsive mechanisms of smart nanoplatforms for controlled drug delivery.

### 5.6 From stimuli-responsive nanocarriers to adaptive therapeutic systems

Existing reviews typically classify nanoplatforms according to their triggering mechanism, including pH, redox, enzyme, magnetic, or photo-responsive systems. While this taxonomy is useful for material design, it does not adequately describe how modern intelligent nanotherapeutics function during therapy. Rather than acting as isolated delivery vehicles, next-generation nanoplatforms increasingly integrate sensing, imaging, therapeutic activation, and adaptive feedback into a unified system. Consequently, the future of nanomedicine should be viewed not as a collection of independent stimuli-responsive materials but as programmable adaptive therapeutic ecosystems capable of continuously responding to changing biological conditions.

## 6 4D nanostructures and shape-adaptive systems

### 6.1 Concept of 4D nanomaterials: time-dependent structural transformation

Four-dimensional materials are so designed to achieve programmable changes in their three-dimensional structure with time to allow actuation by environmental signals or external forces. Such a temporal aspect introduces a new paradigm for engineering advanced nanodevices that can change their morphology, surface properties or internal structure in response to a particular spatiotemporal signal in complex biological systems.^82^ Such dynamic structural reconfigurations allow functionalities such as on-demand drug release, targeted cellular interactions and tunable imaging contrast, achieving better effects than fixed nanomaterials.^190^ This flexibility enables even greater drug-delivery accuracy, as nanostructures can move through a complex biological organism and release their cargo only upon arrival at a particular disease microenvironment, thereby minimizing the off-target effects.^191^ The identical dynamic shift also makes it possible to overcome biological barriers, improve circulation times, and enhance cellular uptake by reproducing biological processes in a natural manner.^192,193^ This programmatic, morphological and functional control provides a distinct advantage in optimizing the effectiveness of nanomedicine, avoiding the limitations of symmetric nanocarrier systems. These dynamic systems, especially the anisotropic systems with elongated rod or worm-like shapes, have been shown to have better biological functions, such as improved cell uptake and increased circulation lifetimes, when compared to their spherical analogs.^194,195^ The transition between various shapes (*e.g.*, between spheres and rods or the other way around) in response to certain signals can also maximize biodistribution, cellular uptake, and delivery of therapeutic payloads.^196^ Such advanced shape switching could be exploited to maximize pharmacokinetics and pharmacodynamics; for example, nanoparticles could be altered to adopt a size and shape that would allow them to stay in the bloodstream for a longer time, followed by conversion into a form that would allow increased tissue penetration and cellular uptake at the target location.^197^ This flexibility is further illustrated by systems that reorganize their conformation to achieve certain functions and thus constantly change in response to variations in their environment.^198^

### 6.2 Shape-morphing nanoparticles and smart assemblies

Advanced techniques use stimuli-responsive materials that produce the desired changes in size, shape, and surface properties, thus optimizing their engagement with biological barriers and enhancing therapeutic efficacy.^1,199^ As an example, it has been demonstrated that nanoparticles that have been designed to shape-shift are able to overcome kinetic barriers, afford reconfigurable structures that switch between alternative assembled mesophases, and increase cellular uptake.^200^ An example of this principle is deformable nanocarriers, which can switch their sizes and undergo morphological changes, promoting tumor-targeted aggregation, increased drug penetration and retention, and controlled drug release.^201^ Such dynamic morphological changes that include the conversion of morphological features, like spherical into rod-like morphologies, can be significant in influencing the rate and pathway of cellular internalization processes, in which anisotropic geometries are likely to be more effective for uptake compared to their isotropic counterparts.^202^ This includes the examples of rod-shaped particles that have increased cellular incorporation and worm-shaped carriers that have increased tissue penetration over the traditional spherical designs.^203^ This underscores the significance of shape control in order to maximize drug-delivery efficiency since morphology directly influences *in vivo* fate, biodistribution and cellular uptake of nanocarriers.^204–206^ Besides, a strategic combination of molecular self-assembly and stimulus-responsive smart materials allows for the design of such carriers rationally, which allows for precise structure–function relationships to be obtained during intelligent delivery.^207^ These versatile transformable nanomaterials that may be reconfigured with physical morphologies for different physiological situations are now considered a game changer in enhanced tumor theranostics to overcome the limitation of nanoparticles with fixed physical morphological features.^208^ This self-adaptive feature enables customized therapeutic effects, which are dynamically changed in response to the complicated, heterogeneous tumor microenvironment, maximizing both targeting and drug release dynamics.^209^

### 6.3 Programmable and self-regulating nanostructures

These new nanostructures will be redesigned to autonomously tune their operation, *e.g.*, drug release and targeting, with built-in feedback that responds to real-time changes in the microenvironment of disease.^5^ The latter can typically be accomplished with high-end material programming, where parts are responsive to any endogenous signal, such as pH response, redox functionality, or enzymatic activation, and will participate in a cascade of coordinated disassembly or controlled activations.^2^ Exogenous triggers, such as near-infrared light or magnetic fields, can be added to enable external control of drug release kinetics and localization.^29,33,210^ This system of two-way control allows extremely precise and adaptable therapeutic interventions, minimizing the off-target effects and providing the therapeutic payload to the target site as accurately as possible.^51^ This programmability is what enables some of the smarter nanomedicines to traverse the complex physiological systems and sense and react to the tiniest pathological indicators.^211^ This involves self-assembly nanocomposites, which can be dynamically assembled to create stimulus-responsive nanostructures, enhancing bioavailability, stability, and reducing systemic toxicity.^212^ A nano-theranostic system that can simultaneously monitor both disease progression and therapeutic responses in a non-invasive way and respond to therapeutic responses in real time on a cellular and molecular level is a brilliant step forward in the area of personalized medicine.^18^ Advanced nanoplatforms may be designed to respond to a particular tumor microenvironmental infrastructural (pH, temperature, enzyme activity, *etc.*) signal to trigger the controlled release of therapeutic agents and thus maximize drug activity and minimize toxicity in the system.^9^ To illustrate this point, the functionalized liposomes targeted using anti-CD44 and anti-PD-L1 DNA aptamers can be specifically used to target breast cancer cells and deliver Doxorubicin and IDO1 siRNA to induce immunogenic cell death and prevent IDO1 expression.^213^

### 6.4 Impact of structural dynamics on cellular uptake and therapeutic efficacy

These smart nanocarriers have significant interactions with cellular machineries, and their flexible structural variations can influence endocytosis pathways, intracellular trafficking, and the final therapeutic efficacy. This has enabled the enhancement of both drug-delivery efficiency through the avoidance of many biological barriers and the intracellular localization of drugs within target cells, leading to improved therapeutic outcomes.^214,215^ The nano-carrier morphology, size and surface chemistry can be optimized to attain improved therapeutic performance through sophisticated material engineering.^216^ This provides a highly diverse array of therapeutic advantages, such as highly enhanced drug encapsulation, increased circulation, and good penetration within complex tissue structures.^217^ This optimization can be generally translated into better patient outcomes, as more focused and effective delivery of drugs can be conducted by revolutionizing treatment options in a variety of diseases, especially cancer.^6,11^ These smart responsive nanoplatforms combine therapeutic and diagnostic functions and are designed to react to internal and external stimuli, like pH, temperature, and redox potential, in order to release regular drugs and track real-time images.^48^ Other advanced systems may include self-regulated systems, in which the release of therapeutic agents may be carefully regulated by a self-regulating feedback loop sensitive to disease biomarkers, improving therapeutic efficacy and reducing off-target effects.^10,16^ These smart nanocarriers incorporate aptamers, which can be precisely targeted to diseased cells, and the minimum systemic toxicity is achieved with high specificity.^218,219^ As an example, pH-specific i-motif sequences were conjugated to gold nanostars and employed in combined cancer treatment, leveraging dynamic DNA nanostructures to actively target disease sites.^220^ Besides, DNA-based nanostructures are remarkably versatile, enabling versatile adoptions and incorporation of different therapeutic functional groups to form multimodal nanosystems with superior functionalities, including near-infrared photoresponsiveness or magnetic targeting.^221,222^ The specific tunability of DNA nanocarriers with respect to size, shape, and location of ligands further enables robust assembly and programmed loading of drugs, thereby reducing batch-to-batch variability^223^ (Fig. 5).

**Fig. 5:**
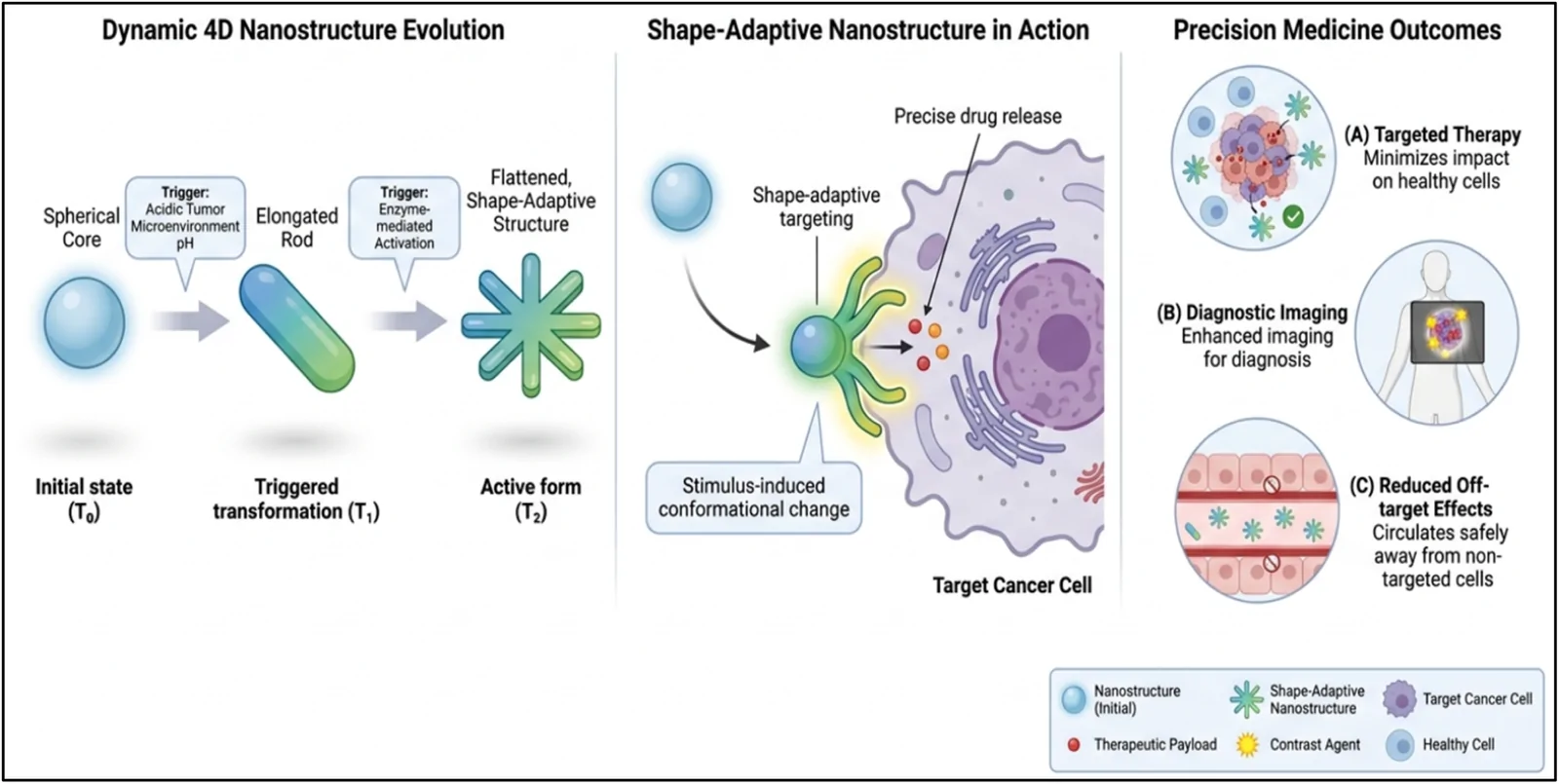
Dynamic 4D nanostructures and shape adaptive systems in precision nanomedicine.

Recent experimental studies have demonstrated that shape-adaptive nanomaterials can dynamically alter their morphology in response to endogenous stimuli, such as acidic pH, enzymatic activity, redox conditions, or externally applied physical triggers, thereby improving their biological performance. For example, transformable nanoparticles capable of switching from spherical structures with prolonged blood circulation to elongated or fibrous assemblies after reaching the tumor microenvironment have shown enhanced tumor-site retention, deep tissue penetration, and improved intracellular accumulation. Other multistage systems undergo size reduction or *in situ* self-assembly following enzymatic activation, facilitating improved diffusion through dense tumor matrices while enhancing imaging contrast and therapeutic efficacy. When integrated with fluorescence, photoacoustic, magnetic resonance, or radionuclide imaging, these adaptive nanoplatforms enable real-time visualization of nanoparticle redistribution, treatment monitoring, and image-guided drug delivery. Despite these encouraging preclinical findings, further optimization of transformation efficiency, biosafety, scalable manufacturing, and long-term *in vivo* evaluation will be necessary before clinical translation can be achieved.^200^

## 7. Feedback-controlled and closed-loop therapeutic systems

### 7.1 Real-time imaging-guided drug release mechanisms

The convergence of advanced imaging modalities with stimuli-responsive nanoplatforms has the potential to enable real-time monitoring of drug delivery, biodistribution, and release kinetics, thereby supporting adaptive therapeutic interventions.^224,225^ Despite these promising capabilities, the clinical translation of closed-loop image-guided therapeutic systems remains at an early stage. Major challenges include achieving reproducible synchronization between imaging signals and therapeutic responses, ensuring quantitative accuracy across different imaging platforms, and integrating heterogeneous imaging and biological datasets into reliable decision-support frameworks. In addition, regulatory pathways for multifunctional theranostic systems remain poorly defined because such platforms combine diagnostic imaging, drug delivery, and, in many cases, AI-assisted decision-making within a single product, substantially increasing manufacturing and validation requirements.^224,225^

DNA nanotechnology exemplifies both the opportunities and limitations of next-generation programmable nanomedicine. DNA-based nanocarriers can be engineered to respond to endogenous stimuli, such as ATP concentrations or pH variations, within the tumor microenvironment and can dynamically reconfigure their structures to modulate drug release or activate therapeutic functions.^226–229^ While these properties provide exceptional programmability, spatial precision, and biocompatibility, significant barriers must be overcome before routine clinical implementation can be achieved. These include achieving large-scale, cost-effective manufacturing, long-term structural stability under physiological conditions, reproducibility across production batches, and standardized quality-control protocols.^230,231^ Furthermore, although DNA nanostructures can simultaneously incorporate diverse therapeutic payloads, including small molecules, proteins, and nucleic acids, increasing multifunctionality also increases manufacturing complexity, analytical characterization requirements, and regulatory scrutiny.^232^

Similarly, the development of DNA-based nanorobots, biosensors, and programmable DNA origami platforms illustrates the expanding scope of precision nanomedicine beyond conventional drug delivery.^233–235^ However, most reported systems remain at the proof-of-concept or preclinical stage, with limited evidence regarding long-term safety, clinical scalability, cost-effectiveness, and interoperability with existing clinical imaging workflows. Future research should therefore prioritize standardized manufacturing processes, robust preclinical validation, multimodal data integration, explainable AI-driven therapeutic decision support, and harmonized regulatory frameworks to facilitate clinical translation. Addressing these challenges will be essential for realizing the theranostic potential of programmable DNA nanostructures for real-time treatment monitoring and adaptive precision oncology.^236,237^

### 7.2 Biosensing interfaces for microenvironment monitoring

Biointerfaces incorporating molecular recognition elements and signal transduction mechanisms have demonstrated the ability to detect subtle physiological changes, including variations in biochemical markers, pH, temperature, and oxygen concentrations, with potential applications in adaptive therapeutic systems.^238^ However, the clinical implementation of such closed-loop platforms remains constrained by the need for highly reliable sensor performance, robust signal reproducibility, and seamless integration of biosensing outputs with therapeutic decision-making. Although machine learning and advanced bioinformatics offer opportunities to interpret complex physiological datasets and support real-time therapeutic optimization, these computational approaches require extensive validation using large, diverse clinical datasets and must address challenges related to model interpretability, data standardization, cybersecurity, and regulatory approval before routine clinical deployment.^239^

DNA nanostructures provide exceptional spatial addressability, enabling precise positioning of biorecognition elements and therapeutic components to improve molecular targeting and control over drug release kinetics within heterogeneous disease microenvironments.^240–243^ Nevertheless, translating these design advantages into clinically robust platforms remains challenging because precise molecular engineering alone does not guarantee reproducible *in vivo* performance. Biological variability, structural stability under physiological conditions, scalable manufacturing, and standardized quality-control procedures continue to limit technological readiness despite encouraging preclinical results.^240–243^ Recent advances in dynamic and stimuli-responsive DNA nanotechnology have expanded the range of programmable diagnostic and therapeutic systems capable of responding to diverse endogenous and exogenous stimuli, including nucleic acids, ions, pH, and temperature changes.^244–247^ Despite these innovations, most platforms remain at an early stage of development, with limited evidence supporting long-term safety, manufacturing scalability, clinical interoperability, and cost-effectiveness. Future research should therefore prioritize standardized evaluation frameworks, integration with clinically validated multimodal imaging and AI-assisted decision-support systems, comprehensive regulatory guidance for multifunctional nanodevices, and large-scale translational studies to bridge the gap between promising laboratory demonstrations and routine clinical practice.^244–247^

### 7.3 Closed-loop systems: from sensing to actuation

Integrated feedback systems that couple real-time physiological measurements from biosensing interfaces with stimuli-responsive nanotherapeutics represent an emerging strategy for adaptive drug delivery and precision medicine.^248^ Although these platforms have demonstrated encouraging proof-of-concept performance, their technological readiness remains limited by challenges in achieving reliable sensor–therapeutic communication, maintaining system stability under dynamic physiological conditions, and ensuring reproducible therapeutic responses across diverse patient populations. The development of autonomous closed-loop therapeutic systems will also require rigorous validation of sensor accuracy, computational decision-making, and long-term safety before clinical implementation.^249^ Compared with conventional therapeutic approaches, closed-loop nanomedicine offers the theoretical advantage of dynamically responding to the spatial and temporal heterogeneity of disease microenvironments.^250^ However, this potential has yet to be translated into routine clinical practice because of unresolved issues related to device integration, interoperability between biosensors, imaging platforms, and therapeutic components, as well as the absence of standardized regulatory pathways for multifunctional adaptive systems. In addition, manufacturing complexity, quality assurance, and cost-effectiveness remain significant barriers to large-scale clinical adoption.^250^

DNA nanorobots further illustrate the promise of programmable nanomedicine through their ability to navigate biological environments, recognize molecular stimuli, and deliver therapeutic payloads with high spatial precision.^251,252^ Nevertheless, most applications remain confined to experimental settings, with limited evidence supporting long-term *in vivo* stability, immune compatibility, scalable manufacturing, and reproducible clinical performance. Future research should therefore prioritize robust translational studies, standardized production protocols, integration with clinically validated bioelectronic and AI-assisted decision-support systems, and harmonized regulatory frameworks. Addressing these challenges will be essential before the combination of DNA nanotechnology and next-generation bioelectronics can realize its anticipated role in personalized medicine and intelligent therapeutic systems.^253^

### 7.4 Quantitative models for feedback regulation

Computational models are expected to play a central role in future closed-loop nanotherapeutic systems by translating real-time biological data into optimized therapeutic decisions and adaptive drug-delivery strategies.^254,255^ However, achieving reliable clinical implementation will require computational frameworks capable of integrating heterogeneous biochemical, imaging, and physiological datasets while maintaining robustness, interpretability, and reproducibility across diverse clinical settings. The validation of these algorithms remains a significant challenge, particularly because predictive performance depends on large, high-quality datasets and standardized data acquisition protocols that are currently lacking.^254,255^

Advances in bioimaging, feedback control, and programmable nanorobotics are anticipated to improve the spatial and temporal precision of autonomous therapeutic systems, including coordinated behaviors within complex biological environments.^256^ Nevertheless, these technologies remain largely experimental, with substantial barriers related to real-time control accuracy, system reliability, manufacturing scalability, and regulatory approval. Similarly, DNA-based neural networks and AI-integrated nucleic acid amplification technologies represent innovative approaches for programmable biochemical computation and biomarker-driven therapeutic decision-making.^257,258^ Despite their conceptual promise, evidence supporting their clinical feasibility, reproducibility, and long-term operational stability remains limited, highlighting the need for rigorous translational validation.

Algorithm-guided DNA molecular machines and AI-enabled nanorobotic platforms have been proposed as next-generation systems capable of autonomous therapeutic regulation and highly specific biochemical responses.^259^ However, their technological readiness remains low because of unresolved challenges in computational transparency, safety validation, quality assurance, and integration with existing clinical workflows. Future research should therefore focus on explainable AI, standardized benchmarking of intelligent nanotherapeutic platforms, scalable manufacturing strategies, and harmonized regulatory frameworks to support clinical translation.^120,157^ In parallel, the integration of these technologies with patient-specific digital twins represents a promising long-term direction for predictive therapeutic modeling and personalized treatment optimization, although this vision will require robust multimodal data integration, prospective clinical validation, and interoperable digital health infrastructures before widespread implementation becomes feasible.^260^

### 7.5 Integration of artificial intelligence for predictive therapeutics

The convergence of AI and nanotechnology has emerged as a promising strategy for improving nanocarrier design, drug-delivery optimization, and pharmacokinetic modelling.^261,262^ Although AI-driven predictive models have demonstrated the ability to accelerate the identification of optimal nanomaterial properties, drug–nanoparticle interactions, and targeting strategies, their clinical utility remains dependent on the availability of high-quality, standardized, and representative datasets.^263,264^ Variability in experimental protocols, limited interoperability among data sources, and insufficient external validation continue to restrict the generalizability of current AI models, highlighting the gap between computational performance and real-world clinical applicability. AI also offers opportunities to support personalized nanomedicine by integrating molecular, imaging, and biosensor-derived data to guide patient-specific therapeutic selection and dosing strategies.^265–267^ However, translating these capabilities into routine clinical practice requires overcoming significant challenges related to multimodal data integration, algorithm transparency, bias mitigation, privacy protection, and regulatory oversight. Furthermore, AI-assisted treatment recommendations must demonstrate consistent clinical benefit through prospective validation and integration within existing healthcare workflows before they can be widely adopted.^265–267^

The application of AI to nanopharmacology has the potential to improve the design, optimization, and evaluation of nanoscale drug-delivery systems, including the prediction of *in vivo* behavior, optimization of drug loading and release kinetics, and individualized therapeutic planning.^268–273^ Nevertheless, most reported studies remain confined to computational or preclinical settings, with limited evidence of scalable manufacturing, reproducible clinical performance, or cost-effectiveness. Future research should therefore prioritize standardized benchmarking datasets, explainable and clinically interpretable AI models, prospective multicenter validation, integration with digital health infrastructures, and harmonized regulatory frameworks. Addressing these priorities will be essential for translating AI-enabled nanomedicine from proof-of-concept investigations into clinically deployable precision therapeutics^268–273^ (Fig. 6 and Table 8).

**Fig. 6:**
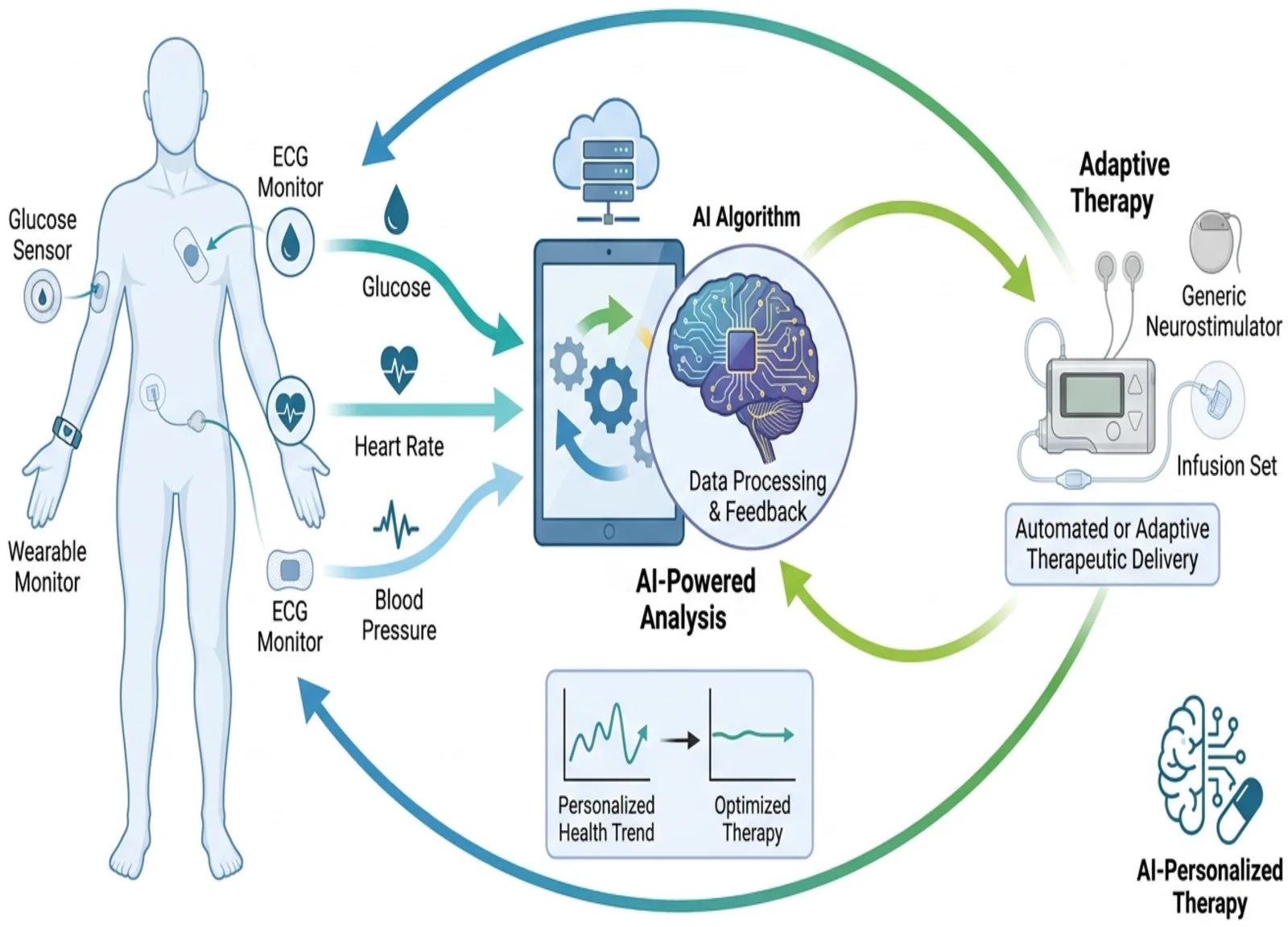
Feedback controlled and closed loop therapeutic systems: from real time sensing to AI-powered personalized therapy.

**Table 8:** Critical comparison of current smart nanotherapeutic strategies

Strategy	Major advantage	Major limitation	Clinical maturity	Remaining challenge
pH responsive	Simple design	Low specificity	Moderate	Tumor heterogeneity
Redox	Intracellular targeting	Variable GSH	Moderate	Patient variability
Enzyme	High specificity	Enzyme heterogeneity	Early	Biomarker validation
NIR	Excellent control	Limited penetration	Moderate	Deep tumors
Ultrasound	Deep penetration	Acoustic variability	Advanced	Standardization
Magnetic	Remote guidance	Low accumulation	Early	Clinical hardware
Multi-trigger	Higher precision	Increased complexity	Emerging	Manufacturing

Although AI-assisted therapeutic control has demonstrated considerable promise in experimental studies, current systems remain largely dependent on pre-trained datasets collected under controlled laboratory conditions. Their ability to adapt to the substantial biological heterogeneity encountered in clinical oncology remains insufficiently validated. Furthermore, real-time closed-loop decision-making requires rapid multimodal data integration, standardized imaging biomarkers, and regulatory frameworks that remain underdeveloped. Consequently, AI should currently be regarded as an enabling technology rather than a fully autonomous controller of therapeutic decision-making.

### 7.6 Artificial intelligence: current challenges and clinical implementation

Artificial intelligence (AI), particularly deep learning, has emerged as an important tool for image reconstruction, segmentation, feature extraction, quantitative biomarker analysis, and treatment-response prediction in nanoimaging.^251^ Convolutional neural networks (CNNs) remain the most widely used architecture for medical image analysis, while transformer-based models and hybrid deep learning frameworks are increasingly being explored for multimodal image integration and complex spatiotemporal data analysis. These approaches have demonstrated promising performance in improving image quality, accelerating image reconstruction, and enabling automated interpretation of molecular imaging data.^253^

Despite these advances, successful clinical implementation depends on the availability of large, diverse, and well-annotated datasets. Models developed using limited or single-center datasets may exhibit reduced generalizability when applied to different patient populations, imaging systems, or acquisition protocols.^254^ Consequently, rigorous external validation, multicenter evaluation, and standardized reporting of model performance are essential to ensure robustness and reproducibility. Another important consideration is the interpretability of AI algorithms. Although deep learning models often achieve excellent predictive performance, their complex decision-making processes may reduce clinician confidence and hinder regulatory acceptance. Explainable artificial intelligence (XAI) techniques, including attention maps, feature attribution methods, and saliency visualization, are increasingly being adopted to improve model transparency and facilitate clinical interpretation.^255,256^

AI systems may also be affected by algorithmic bias arising from imbalanced datasets, demographic variability, or differences in imaging equipment and institutional protocols. Such biases can influence diagnostic performance and limit equitable clinical implementation. Careful dataset curation, standardized validation procedures, and continuous post-deployment monitoring are therefore necessary to ensure reliable performance across diverse clinical settings.^257^

Finally, the clinical adoption of AI-assisted nanoimaging requires compliance with evolving regulatory frameworks governing software as a medical device (SaMD), data privacy, cybersecurity, and continuous algorithm updates. Close collaboration among imaging scientists, clinicians, computer scientists, and regulatory authorities will be essential for the safe and effective integration of AI into future nanoimaging-guided precision medicine.^258^ Although AI has considerable potential to enhance nanoimaging-guided precision therapeutics, its successful clinical implementation will depend not only on algorithmic performance but also on robust validation, interpretability, fairness, and regulatory compliance.

## 8. Spatiotemporal control of therapeutic interventions

### 8.1 Precision targeting and controlled drug distribution

Precision targeting is achieved by localizing therapeutic agents to particular disease locations with minimal systemic exposure, commonly using active targeting schemes that leverage certain molecular markers on diseased cells or passive targeting using the accumulation of nanoparticles in regions with leaky vasculatures. Such precision is essential to achieving the maximum therapeutic efficacy and minimizing side effects, which is the main focus of precision medicine.^128,274^ This dynamic spatiotemporal control goes beyond the initial localization of the therapeutic agent to the control of the activity and concentration of the therapeutic agent in the target microenvironment with time, which is frequently achieved through stimuli-responsive drug–release processes. These control systems are required to tightly tune the availability of drugs to changes in the disease microenvironment in real-time to guarantee long-term therapeutic efficacy and reduce collateral damage to normal tissues. This involves optimizing the size, surface charge and conjugation of ligands to maximize accumulation at pathological sites and ensure effective internalization in cells.^158,275^ The latter can also be enhanced using AI-inspired nanoarchitectonics, facilitating the synthesis of hierarchical nanostructures that provide a high degree of spatial and temporal control of drug release and localization.^276^ Nanoparticles can also be used for multi-material printing, which allows for the formation of complex nanostructures with multiple functionalities for improved drug delivery and therapeutic effects,^277^ and AI-based nanoparticle design, which further streamlines the functionalities for more specific and effective targeting.^278,279^ This is especially important in the case of the creation of personalized nanocomposite-based drug-delivery systems; with the help of AI and 3D printing, it is possible to precisely regulate drug release kinetics and tailor dosage forms.^280^ This new convergence of technologies opens the door to extremely personalized medicine, whereby treatment regimens are tailored to each patient, with significant improvements to the treatment outcome and patient compliance.^58,281^

### 8.2 Temporal programming of drug release

This encompasses the coordination of the release of therapeutic agents within a predefined time interval or in response to certain biological signals to sustain optimal concentrations of drugs at the target site and reduce systemic exposure and toxicities. Engineered nanocarriers can execute this temporal programming to achieve either pulsatile or sustained *in vivo* release profiles, frequently controlled by endogenous or exogenous stimuli.^282^ Such an advanced regulation of release kinetics plays a vital role in managing chronic diseases, avoiding drug resistance, and improving therapeutic indices through matching the availability of drugs to the development of diseases or circadian cycles. Besides, AI algorithms that aid the temporal programming of drug release can optimize release profiles according to real-time physiological data and disease progression models.^164,283^ This enables optimal adjustments to drug delivery in an adaptive manner to meet the changing needs of the patient, achieving the maximum therapeutic effect and reducing detrimental effects.^284^ This integration of state-of-the-art materials with computational intelligence enables one to design complex drug-delivery systems that will dynamically respond to changing biological conditions to provide optimal therapeutic doses at the right place and at just the right time. Machine learning also contributes to this adaptive capacity since drug-release-rate adjustments can be made on a real-time basis in response to ongoing physiological feedback in order to enhance the accuracy and effectiveness of drug delivery.^285^ These AI-driven systems adopt predictive modeling to make the best decisions on the drug formulation and drug behavior to ensure a paradigm shift to ultra-personalized and effective therapeutic interventions.^286,287^ Programming drug-delivery systems based on artificial intelligence and machine learning represents a decisive move towards a personalized approach to medicine with the possibility of monitoring the patient in real-time and establishing adjustable controls to achieve the best therapeutic effect and minimize side effects.^288,289^ Such a high-level integration makes it possible to adjust drug release dynamically, unlike traditional approaches, and to provide more accurate and convenient administration of therapeutic agents.^290^

### 8.3 Enhancing therapeutic index and reducing off-target effects

Therapeutic interventions that achieve the best effect on pathological cells or tissues and, at the same time, reduce the impact on normal tissues are important. Such interventions are obtained by taking advantage of advanced delivery systems, including targeted nanocarriers and stimuli-responsive systems, which selectively accumulate and change in diseased sites, delivering therapeutic agents to where they are most needed and avoiding the unwanted activation of normal tissues.^291^ The ability of 4D nanoimaging to provide the fine spatiotemporal control of drug localization and drug release kinetics, which is necessary to attain the optimal therapeutic and the minimal systemic toxicity effects, enables such strategies. This is further maximized with AI algorithms that predict the optimal drug concentrations and release profiles, thus reducing adverse events and enhancing patient outcomes.^292,293^ This predictive capability can be employed to maximize drug dosages and treatment regimens, eventually leading to an increased therapeutic index and reduced off-target toxicities.^294,295^ This approach to analysis facilitates easy design and optimization of smart nanoparticles, elimination of limitations related to the conventional drug-delivery system, and enhancement of therapeutic processes.^5,54^ This approach can be applied to eliminate drug-discovery bottlenecks by integrating AI in nanomedicine, enabling the design of drugs in the most optimal manner; the information and predictive-analysis results provided by AI will enhance the effectiveness of treatment and targeted delivery.^296,297^ The capability of AI to process multidimensional data also enables the differentiation of properties of nanocarriers, such as size, shape, and surface chemistry, to improve biodistribution and persistence under biologically complex conditions.^298,299^ The prediction of nano-bio interactions, *e.g.*, protein-corona formation, and optimization of drug loading and release kinetics can also be considered an additional application of AI^300^ (Table 9).

**Table 9:** Spatiotemporal control strategies in 4D nanoimaging-guided smart therapeutics

Parameter/focus	Scientific description	Technological strategies	Therapeutic and clinical implications	Ref.
Precision targeting	Selective localization of therapeutic agents at disease sites *via* active (ligand–receptor) and passive (EPR effect) targeting mechanisms	Ligand conjugation, optimization of nanoparticle size and surface charge, AI-driven nanoarchitectonics, 3D/multi-material printing	Enhances drug accumulation at target sites, improves cellular internalization, and minimizes systemic toxicity	128, 158, 274 and 275
Controlled drug distribution	Regulation of drug concentration and activity within the target microenvironment over time	Stimuli-responsive nanocarriers, hierarchical nanostructures, AI-guided nanoparticle design	Ensures sustained therapeutic efficacy and minimizes collateral damage to healthy tissues	58, 180, 276 and 281
Temporal programming of drug release	Controlled release of therapeutics over time or in response to biological/external stimuli	Pulsatile and sustained release systems, stimuli-responsive polymers, smart nanocarriers	Aligns drug release with disease progression, improves therapeutic window, and reduces drug resistance	282
AI-driven adaptive drug delivery	Dynamic adjustment of drug-release profiles based on real-time physiological data and predictive modeling	Machine learning algorithms, predictive analytics, and real-time feedback systems	Enables personalized therapy with optimized dosing, improved efficacy, and reduced adverse effects	164, 283, 286, 288 and 290
Enhancement of therapeutic index	Maximization of therapeutic efficacy while minimizing toxicity through targeted and controlled delivery	Stimuli-responsive nanocarriers, real-time nanoimaging feedback, AI-based dose optimization	Improves treatment outcomes and reduces off-target effects in complex diseases, such as cancer	291, 294 and 295
AI-optimized nanocarrier design	Data-driven customization of nanoparticle properties for optimal biological interaction	AI/ML-based modeling of size, shape, surface chemistry, and protein-corona interactions	Enhances biodistribution, cellular uptake, and drug release efficiency	5, 54, 296, 298 and 300
Conventional *vs.* smart nanomedicine	Comparison between static nanocarriers and adaptive 4D nanoimaging-guided systems	Integration of real-time imaging, feedback loops, and AI-enabled adaptive delivery systems	Demonstrates superiority of smart therapeutics in precision, adaptability, and reduced toxicity	126, 127, 131, 301 and 302
Computational and *in silico* optimization	Use of advanced computational tools to design and predict nanocarrier behavior	AI platforms (*e.g.*, *in silico* tumor modeling), support vector machines, neural networks	Accelerates nanomedicine development, improves formulation stability, and enables personalized treatment strategies	11 and 303–306

### 8.4 Comparative analysis with conventional nanomedicine

Although passive targeting and sustained release are central to traditional nanomedicine, 4D nanoimaging-based smart therapeutics incorporate dynamic feedback loops, which allow adaptive drug delivery based on dynamic changes in the microenvironment. This feature represents a significant improvement from unresponsive nanocarriers to adaptive systems that are able to regulate therapeutic responses in a highly controlled manner in terms of physiological needs.^301^ This adaptive, AI and machine learning-mediated approach allows real-time, personalized modulation of treatment, ensures optimum drug activity and reduced systemic toxicity, and overcomes the constraints of traditional, non-adaptive nanomedicine.^126,127,131,302^ This paradigm shift employs advanced computational models to project biodistribution, nano-drug loading and nano-bio interactions to simplify the design and development of next-generation nanocarriers.^11,303^ Furthermore, it is possible to predict the *in silico* tumor microenvironment using AI-based systems, such as EVOnano, allowing the optimization of nanocarrier properties and swiftly creating adaptable nanomedicines with a high tumor specificity.^304^ AI-based systems represent a highly developed technology, requiring the application of computational analysis to examine the relationships between drugs and nanoparticles and determine the encapsulation efficiency, which promotes more stable and efficient preparations.^305^ Machine learning models, including support vector machines and artificial neural networks, further streamline nanoparticle design, simulating drug release profiles and reshaping therapeutic regimens to patient-specific data^306^ (Fig. 7).

**Fig. 7:**
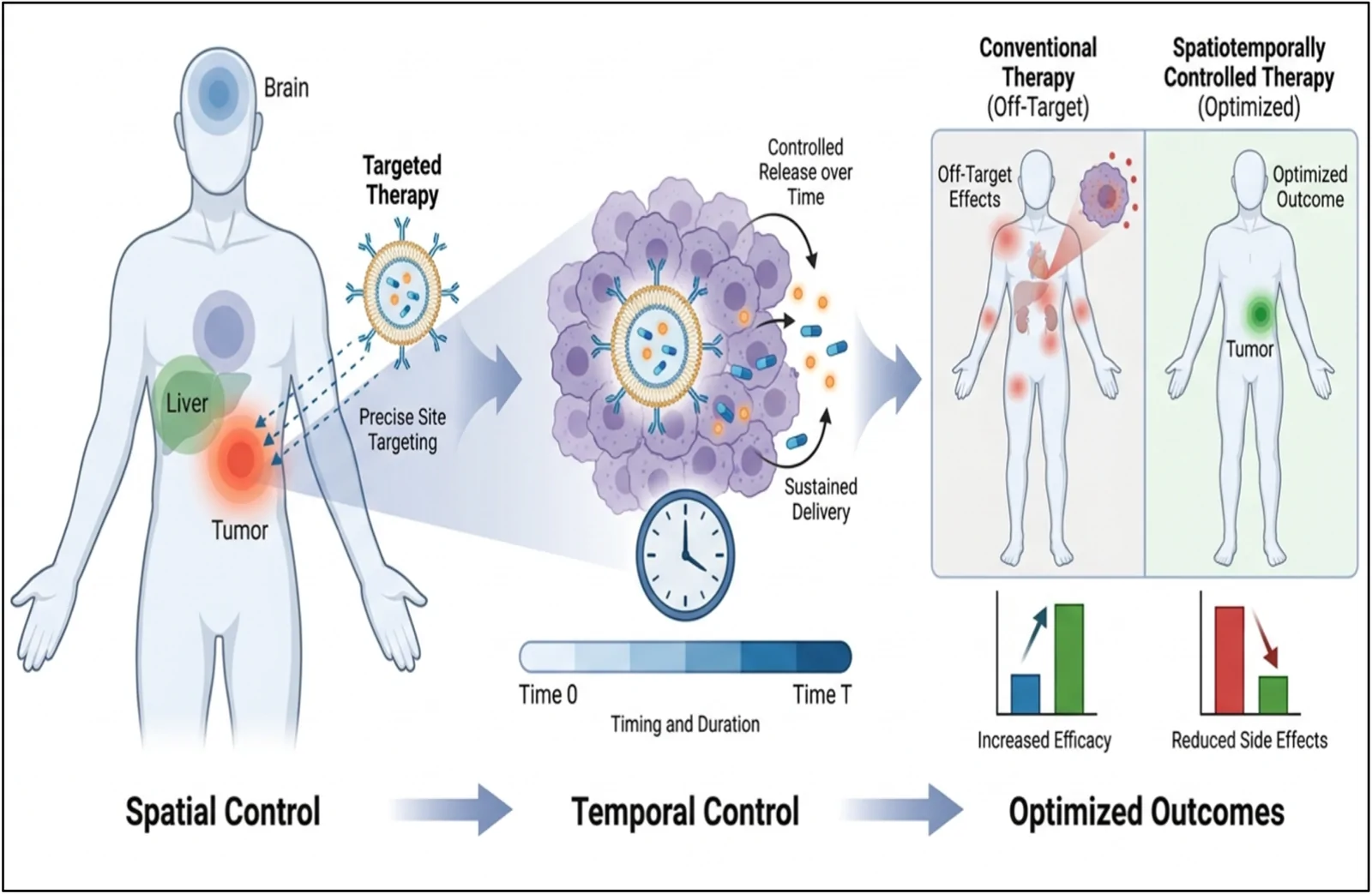
Spatiotemporal control of therapeutic interventions.

## 9. Applications in disease models

### 9.1 Cancer: tumor progression monitoring and targeted therapy

In cancer research, 4D nanoimaging provides the initial data concerning the spatial–temporal dynamics of tumor development and treatment effects and can assist a researcher to accurately target rogue cells, without damaging normal tissues.^307^ It enables the formation of small-sized nanocarriers that have the ability to target tumors with higher permeability, retention and real-time efficacy and biodistribution.^82,123^ This type of advanced imaging with AI-powered analysis will be capable of tracking the precise localization of therapeutic agents within the multidimensional tumor microenvironment and providing a comprehensive view of the kinetics of treatment and optimal dosage, allowing personalized treatment of cancer.^308^ This feedback control, in real time, makes it possible to implement adaptive changes to treatment regimens, reducing resistance mechanisms and enhancing long-term therapeutic responses.^36^ Combining preclinical MRI and CT methods also makes it feasible to develop personalized nanotheranostics since it offers a good understanding of the distribution, accumulation, and therapeutic benefits of such nanoparticles.^111^ This allows for the formation of multifunctional nanocarriers that have the capacity to carry out diagnostics, real-time monitoring of drug delivery and targeted treatment of cancers simultaneously.^51^ These techniques alone and together allow for real-time measurement of tumor response to nanotherapeutics, which can be tuned to the treatment course based on various stimuli to guarantee optimal therapeutic outcomes and minimal off-target effects. Such fine control of these spatiotemporal distributions of therapeutic agents in tumors can be significantly enhanced by Monte Carlo simulations; these can be used to model the interactions of nanomaterials with biological tissues to forecast contrast enhancement and optimize dosimetry of radiation-based imaging procedures.^122^ These simulations are important in understanding the biodistribution of nanoparticles and simplifying treatment strategies in dynamic and complex tumor microenvironments.^309^

### 9.2 Inflammatory diseases and immune modulation

Likewise, in autoimmune and inflammatory diseases, 4D nanoimaging can be used to trace the localization of smart nanoparticles at inflammatory locations, making it possible to target immune cells or inflamed tissue with immunomodulatory or anti-inflammatory drugs. This would enable the accurate localization of therapeutic agents in a dynamic immune microenvironment, thus streamlining the process of effective disease management through dose and time control.^20^ This is essential to create personalized nanomedicine that can control immune responses in a highly specific manner, minimize side effects at the systemic level, and improve therapeutic outcomes. This would allow for the creation of nanoparticles that are sensitive to a particular inflammatory biomarker, providing a powerful solution for early detection and treatment of chronic inflammatory diseases.^130^ In addition to therapeutic delivery, 4D nanoimaging provides a powerful platform to assess disease progression and therapeutic response under such dynamic inflammatory conditions in real time and in a non-invasive fashion. This enables the creation of enhanced functional nanomaterials, which combine diagnostic properties with therapeutic applications, for treating disorders of the nerves and heart, as well as inflammatory mechanisms.^310^ Indicatively, nanotechnology has been actively explored to come up with novel systems for targeted drug delivery in cardiovascular disease cases, wherein atherosclerosis and myocardial infarction are combated by fine-tuning cellular mechanisms. Imaging techniques, such as positron emission tomography and single-photon emission computed tomography, are highly sensitive and can be used to monitor radiolabelled nanoparticles, showing their systemic behavior and localization in sites of inflammation.^110^ This can be utilized to carefully assess the nanotherapeutic effectiveness of targeting inflammatory responses, thereby guiding the future creation of highly specific interventions^311–313^ (Table 10).

**Table 10:** Applications of 4D nanoimaging-guided smart nanoplatforms across major disease models

Disease model	Smart nanoplatform	Stimulus/trigger	Imaging modality	Therapeutic strategy	Key outcome	Translational status	Representative ref.
Breast cancer	pH-responsive polymeric nanoparticles	Acidic TME	NIR-II fluorescence	Targeted chemotherapy	Enhanced tumor accumulation, reduced systemic toxicity	Preclinical	307 and 308
Lung cancer	Gold nanorods	NIR irradiation	Photoacoustic imaging	Photothermal therapy	Improved tumor ablation and imaging guidance	Preclinical	309 and 311
Glioblastoma	Lipid nanoparticles	BBB-targeting ligands	MRI + fluorescence	Drug delivery	Improved blood–brain barrier penetration	Preclinical	310, 313 and 316
Hepatocellular carcinoma	Mesoporous silica nanoparticles	Redox-responsive	MRI/PA imaging	Chemo-photothermal therapy	Controlled drug release and superior imaging	Preclinical	321 and 322
Bladder cancer	Hyaluronidase-responsive nanoparticles	Hyaluronidase	NIR fluorescence	Intravesical drug delivery	Increased tumor selectivity	Experimental	317, 319 and 320
Pancreatic cancer	Enzyme-responsive nanocarriers	MMP activation	Photoacoustic imaging	Targeted chemotherapy	Improved penetration through dense stroma	Preclinical	323 and 324
Cardiovascular disease	ROS-responsive nanoparticles	Oxidative stress	MRI	Anti-inflammatory therapy	Reduced plaque inflammation	Experimental	325 and 327
Neurodegenerative disorders	Magnetic nanoparticles	External magnetic field	MRI	Targeted neurodrug delivery	Enhanced brain localization	Early preclinical	318 and 328
Bacterial infection	Enzyme-responsive liposomes	Bacterial enzymes	Fluorescence imaging	Antibiotic delivery	Improved infection localization	Preclinical	311 and 314

### 9.3 Neurodegenerative disorders and blood–brain barrier challenges

A 4D-nanoimaging system would be essential for addressing neurodegenerative disorders because it overcomes the major obstacle of the blood–brain barrier, and it is possible to visualize and accurately deliver therapeutic nanoparticles to the damaged brain areas. The technology can be used to measure the permeation of nanoparticles across the blood–brain barrier in real-time and their relationship with pathological aggregates or abnormal neural cells, thus streamlining the treatment of conditions such as Alzheimer's and Parkinson's disease. An example is an anti-inflammatory agent and anti-A antibody functionalized magnetic iron oxide nanoparticles that have been demonstrated to improve MRI contrast and plaque-related inflammation in Alzheimer's disease models.^314^ The state-of-the-art multimodal imaging systems that combine fluorescence and photoacoustic imaging have the potential to offer high-resolution measurements of the spatial and temporal distributions of nanotherapeutics in the central nervous system, which can aid in the development of strategies to bypass the blood–brain barrier.^315^ These spatiotemporal management characteristics are also becoming invaluable in the search for cures for cardiovascular diseases, wherein theranostic nanoparticles are used to diagnose and selectively treat diseases, such as atherosclerosis, which is one of the leading causes of cardiovascular death.^316^ Nanotheranostics, frequently utilizing iron oxide or gold nanoparticles, polymeric nanoparticles, liposomes, and micelles, can be applied to the real-time imaging of atherosclerotic plaques and drug delivery, enhancing diagnostic accuracy and therapeutic results^317^ (Table 10). The constant observation brought about by 4D nanoimaging can inform the therapeutic response, and real-time corrections to the treatment regimens can be made to ensure the therapeutic response is at the highest possible level.^318^ This is especially useful for delivering therapeutic agents across the blood–brain barrier for the treatment of neurodegenerative disease, where traditional delivery of drugs frequently fails.^319^

### 9.4 Tissue regeneration and degenerative diseases

It is our view that 4D nanoimaging offers unparalleled opportunities to track the adoption and efficacy of smart biomaterials and scaffolds loaded with stem cells, capturing the dynamic changes required for cellular differentiation, extracellular matrix remodelling, and tissue regeneration. This technology enables the delivery of growth factors and progenitor cells with high accuracy in real-time and the optimization of regenerative processes in complex biological environments.^320^ This type of imaging can be used to quantitatively measure the repair dynamics of tissues, such as vascularization, cellular growth, and biomaterial degradation, directing the optimization of regenerative medicine strategies. Further, a real-time feedback loop is required to create next-generation regenerative therapies capable of responding to dynamic biological microenvironments, facilitating excellent tissue integration and functional regeneration.^320^ Furthermore, stimuli-responsive nanoplatforms and 4D imaging bring about additional optimization of regeneration strategies through on-demand delivery of therapeutic cargo *via* physiological stimulation, improving tissue repair kinetics and graft survival. This monitoring and control accuracy is particularly important for patients suffering from chronic disorders, such as atherosclerosis. In this context, nanomedicine technologies are already being used to enable sophisticated diagnosis and administer nanofocused therapy, designed to target dedicated pathophysiological functions in the vascular microenvironment.^321,322^ This combined method enables visualization of both plaque stability and the local delivery of anti-inflammatory or anti-proliferative drugs, thus improving the treatment process of cardiovascular diseases. Additionally, next-generation NIR-II platforms, which offer real-time longitudinal imaging with tunable biocompatibility, can be used to deliver therapeutics and provide efficient bioimaging in cardiovascular settings.^25^ This is due to the versatility of 4D nanoimaging and smart nanotherapeutics, which have achieved breakthroughs across various challenging medical conditions, such as neurodegenerative, cardiovascular, and other chronic disorders (Table 10).

Though numerous disease-specific applications of nanoimaging-guided smart therapeutics have been reported, direct comparison across disease models remains challenging because of differences in nanoplatform design, imaging strategies, therapeutic objectives, and stages of clinical development. Table 10 summarizes the principal characteristics, reported outcomes, and translational status of the representative disease-specific applications discussed in this section.

### 9.5 Cardiometabolic disease applications

The integration of 4D nanoimaging with smart nanotherapeutics can potentially transform cardiometabolic disease treatment, due to the capacity to deliver drugs with precision and spatial control; moreover, monitoring of real-time metabolic perturbations can be performed.^3^ This approach makes it possible to dynamically assess therapeutic interventions aimed at controlling metabolic processes, thereby maximizing the treatment regimen of such illnesses as diabetes and dyslipidemia. This integration can guide the administration of insulin mimetics or lipid-lowering agonists to specific organs without systemic side effects and while maximizing therapeutic responses. The study of real-time interactions between nanoparticles and cellular and subcellular components in metabolic tissues offers important information on the mechanisms of action and potential off-target effects, thus supporting personalized medicine strategies in the treatment of underlying complex cardiometabolic diseases. The extensive nature of cardiovascular diseases as a leading cause of death worldwide, makes nanomedicine approaches that utilize cutting-edge imaging highly promising for achieving enhanced diagnosis and therapeutic effects.^323^ In particular, imaging and treatment of vulnerable atherosclerotic plaques can be executed with high specificity using stimuli-responsive nanoplatforms and surface-functionalized nanoagents, which are vital for cardiovascular health management.^324^ One such technique includes ultrafast superparamagnetic iron oxide-enhanced magnetic resonance imaging, which has the ability to combine heterogeneous macrophage infiltration patterns in patients with stroke, facilitating targeted anti-inflammatory therapy.^325^ These developments underscore the relevance of nanotheranostics in providing a holistic disease-management approach, integrating complicated diagnostics with specific therapeutics in the treatment of complicated pathophysiological conditions.^326^ An integrated diagnostic and therapeutic approach is particularly significant in the treatment of chronic conditions, like cardiovascular diseases, where conventional treatment approaches have little impact and often induce long-term systemic adverse effects^327,328^ (Table 10).

Although numerous multifunctional nanoimaging-guided therapeutic systems have demonstrated promising preclinical performance, only a limited number have progressed to clinical implementation. Table 11 summarizes representative clinically established or clinically evaluated nanoimaging-guided technologies, illustrating the current translational landscape and the principal challenges that remain before fully integrated 4D nanoimaging-guided smart therapeutics can be routinely implemented in precision medicine.

**Table 11:** Representative clinical translation of nanoimaging-guided therapeutic platforms

Disease area	Representative nanoplatform/imaging agent	Imaging modality	Clinical status^a^	Primary clinical application	Major translational challenges
Solid tumors	Liposomal doxorubicin (image-guided formulations)	MRI/CT	FDA-approved/clinical	Targeted chemotherapy and treatment monitoring	Limited imaging specificity
Liver cancer	Yttrium-90 microspheres	PET/CT, SPECT/CT	Routine clinical practice	Radioembolization and response assessment	Patient selection and dosimetry
Sentinel lymph node mapping	Indocyanine green (ICG)	NIR fluorescence	Routine clinical practice	Fluorescence-guided surgery	Limited tissue penetration
Neuroendocrine tumors	^68^Ga-labeled radiotracers	PET/CT	Routine clinical practice	Molecular imaging and therapy planning	Radiotracer availability
Prostate cancer	PSMA-targeted radiopharmaceuticals	PET/CT	Routine clinical practice	Tumor localization and treatment guidance	Cost and isotope logistics
Brain tumors	5-ALA fluorescence guidance	Fluorescence imaging	Routine clinical practice	Fluorescence-guided surgery	Limited applicability to all tumor types
Multifunctional theranostic nanoplatforms	Imaging-guided smart nanoparticles	MRI/PET/NIR/PA	Early clinical/preclinical	Combined diagnosis and therapy	GMP manufacturing, regulatory approval, reproducibility

a Clinical status represents the current level of clinical implementation of representative nanoimaging-guided technologies; fully integrated 4D nanoimaging-guided smart therapeutic systems remain largely in the preclinical or early translational stage.

Despite encouraging preclinical outcomes across multiple disease models, most nanoimaging-guided therapeutic systems remain at an early stage of clinical development. Differences in disease biology, patient heterogeneity, and imaging infrastructure continue to limit direct translation. Furthermore, relatively few randomized clinical trials have evaluated these technologies, emphasizing the need for standardized protocols and robust clinical evidence before routine implementation^327^ (Table 12).

**Table 12:** Advantages and current limitations of major technologies

Technology	Major advantages	Current limitations	Future priorities
NIR-II imaging	High sensitivity, low autofluorescence	Limited imaging depth, few approved fluorophores	Clinical validation, safer probes
Photoacoustic imaging	High spatial resolution, functional imaging	Limited penetration, lack of standardization	Quantitative imaging, multicenter studies
PET/SPECT	High molecular sensitivity	Radiation exposure, high cost, infrastructure requirements	Novel tracers, hybrid imaging
AI-assisted reconstruction	Faster reconstruction, improved image quality	Dataset bias, limited explainability, external validation	Transparent AI, standardized datasets
Smart nanoplatforms	Targeted drug delivery, programmable release	Manufacturing complexity, toxicity, scalability	GMP production, long-term safety
Stimuli-responsive systems	Controlled therapeutic activation	Biological heterogeneity, formulation complexity	Multi-stimuli systems, clinical validation

## 10. Quantitative performance metrics and benchmarking

### 10.1 Imaging resolution and penetration depth analysis

In this section, we are going to critically review the spatial, temporal, and sensitivity limits of the other 4D nanoimaging modalities with a keen interest in the depths of penetration of all the modalities in biological tissues and the effects of these modalities in clinical translation. A comparative analysis of these measures in different platforms, such as NIR-II fluorescence and photoacoustic imaging, will bring to light trade-offs between the resolution and depth for a particular application, *e.g.*, drug delivery and deep tissue therapeutic applications. The quantitative methods for identifying the optimal imaging parameters, such as signal-to-noise ratio and contrast-to-noise ratio, will be discussed in terms of detecting and quantifying nanoparticles under complicated biological settings. These modalities will be evaluated based on their capability to distinguish the different types of tissues and pathological states, which are important for the precise diagnosis of diseases and tracking of the progression of treatment options.^329^ This involves an assessment of their ability to follow dynamic disease changes in the microenvironment, including inflammation and angiogenesis, which is essential for defining disease progression and therapeutic efficacy.^330,331^ This type of assessment also requires consideration of the impact of physiological parameters, such as organ- and disease-specific variations, on imaging effects and the interpretation of quantitative imaging biomarkers.^332^

### 10.2 Drug release efficiency and kinetic modelling

In this subsection, more sophisticated approaches to accurately determine the release rate and degree of therapeutic agents by smart nanoplatforms in physiological and pathological conditions will be discussed. Advanced kinetic models will be included in this analysis to estimate and optimize the profiles of drug release to achieve therapeutic effects and reduce off-target effects. Specific emphasis will be placed on the interdependence of different nanoparticle design factors, such as size, shape, and surface chemistry, and their effects on the release kinetics of drugs, particularly with relation to endogenous and exogenous stimuli.^2^ This will be followed by the assessment of drug bioavailability and biodistribution, which are crucial in the overall determination of therapeutic index and clinical utility of such developed nanotherapeutics.^333^ This entails stiff scrutiny of the spatiotemporal regulation of drug discharge, which is significant in enhancing therapeutic impacts and decreasing undesired responses.^28^ These quantitative values play a pivotal role during the assessment of the preclinical effectiveness of bioresponsive nanomaterials and help in their translation into clinical practice.^179^ The difficulties that exist in extrapolating *in vitro* data on drug release to *in vivo* performance are also described, as well as the importance of having strong *in vivo* models and predictive pharmacokinetic/pharmacodynamic (PK/PD) relationships.^71^

### 10.3 Biodistribution and clearance profiles

This article presents a critical analysis of the systemic and tissue localization, as well as the clearance, of smart nanoplatforms, with particular emphasis on the effects of their physicochemical properties on these processes. In-depth knowledge of these profiles, including organ-specific absorption and long-term retention, is key to determining the therapeutic efficacy of these agents and their toxicity potential.^55^ Such factors are essential, requiring rigorous testing, in many cases, with the help of improved imaging methods to predict clinical results and develop safer and more efficient nanomedicines.^334^ Biodistribution strategies (surface functionalization or stimulus-responsive targeting) that provide systemic access to potent agents while enhancing therapeutic indices are described. This entails a keen examination of their interaction with the biological barriers and the immune cells, since this contributes significantly to what they will end up doing in the body. This rigorous assessment would be significant in simplifying delivery schedules and allowing the precise localization of therapeutic agents at disease locations, maximizing therapeutic effects and minimizing undesirable systemic results. Additionally, detailed pharmacokinetics and biodistribution analysis are invaluable in forecasting the action of smart nanoparticles in biological environments and designing nanoparticles to maximize drug delivery to disease-affected areas while avoiding adverse clinical outcomes.^5,335^ Furthermore, although multi-stimuli-responsive systems are more complex, they provide improved precision and require further knowledge in physics, chemistry, and biology to design and build the best systems.^64,336^ It is a big challenge to translate these complex multi-responsive systems from proof-of-concept *in vitro* experiments into robust *in vivo* analyses and finally to clinical trials.^6,16^

### 10.4 Therapeutic efficacy: preclinical *vs.* clinical correlations

In this part, a critical evaluation is carried out on the concordance between the therapeutic outcomes in the preclinical models and the outcomes achieved in human clinical trials, with a particular emphasis on the factors that tend to cause translational gaps. It explores the shortcomings of animal models in accurately projecting the features of human diseases and the heterogeneity of in-patient populations, which can greatly impact the efficiency of drug delivery and the response to therapy.^7^ The identification of predictive biomarkers and the development of stringent preclinical study designs that more effectively recapitulate human pathophysiology to enhance the translatability of new nanotherapeutics will be one of the key elements.^337^ These translational hurdles are also due to the challenges of scaling up production of complex multi-responsive nanoplatforms, as well as assuring their clinical safety.^77^ Therefore, the safety and biocompatibility measurements conducted over short- and long-term periods, including possible immunogenicity and metabolic profile measurements, are essential in progressing these nanomedicines to the next phases of clinical use.^70,338,339^ To overcome these issues, in-depth knowledge of essential variables like pharmacokinetics, biodistribution, and nano-bio interfacial relationships is needed.^10^ Furthermore, the persistent lack of knowledge on the physiological and pathological discrepancies between animal models and humans, especially regarding the ways in which these differences relate to the distribution and efficacy of nanomedicines, only widens the translational gaps.^34^ Thus, to close the preclinical-clinical gap, new experimental methods, advanced analytical models, and adaptive clinical approaches are required.^340^

### 10.5 Technical standardization and methodological validation

The successful translation of 4D nanoimaging-guided smart therapeutics requires standardized imaging protocols, validated analytical methods, and reproducible quantitative measurements.^341^ Variations in imaging hardware, acquisition parameters, reconstruction algorithms, and nanoparticle characterization can significantly affect image quality and therapeutic assessment, making cross-study comparisons difficult. Therefore, the harmonization of imaging protocols and the standardization of reporting practices are essential to improve reproducibility and facilitate multicentre studies.^342,343^

In addition, artificial intelligence-based image analysis requires rigorous validation using diverse datasets to ensure robustness and generalizability across different clinical settings.^344^ Standardized quantitative imaging biomarkers, together with consistent evaluation of nanoparticle pharmacokinetics, biodistribution, and therapeutic response, will further strengthen the reliability of 4D nanoimaging systems and support their future clinical applications.^345,346^

## 11. Translational and clinical considerations

The successful clinical use of nanomedicines relies on their compliance with regulatory pathways, scalability in production, and patient-specific factors, which define their applicability as therapeutic agents, as well as their market adoption.^347^ One of the major hindrances to such translation is the lack of standardized testing requirements for nanoparticles, thus making such regulatory approval costly and increasing the time needed to penetrate the market.^78,348^ What further complicates this landscape is that there was no universally accepted definition or characterization methodologies for nanomaterials, and it was hard to test them in a consistent manner and to perform inter-laboratory comparisons.^349^ Furthermore, the complicated structure and multi-structure of most nanomedicine designs require complex, multi-phase synthesis protocols that are not always standardized and may not have accompanying purification techniques, posing a major limitation in terms of production costs and time.^350^ This is compounded by the significant financial resources that are necessary to develop preclinical and clinical studies, as well as the difficulty of obtaining regulatory clearance, especially for nanomedicines that treat indications already covered by existing products in the market.^351^ All these are reasons behind the long development cycles for new nanodrugs, which may take over a decade and cost more than a billion dollars, requiring convincing clinical advantages to merit the high prices.^352^ Besides, the intricate regulatory environment, defined by the lack of explicit requirements regarding the use of nanomedicines, impedes their translation into clinical practice, causing delays in the approval procedure and ambiguity among manufacturers.^353^

## 12. Clinical translation and future perspectives

Although significant progress has been made in smart nanotherapeutics and advanced imaging technologies, several challenges must be overcome before their routine clinical implementation. In addition to the technical standardization discussed in Section 10.5, successful translation depends on overcoming regulatory requirements, large-scale manufacturing challenges, and comprehensive clinical validation.^354^ Multifunctional nanoplatforms require careful evaluation of safety, biocompatibility, pharmacokinetics, long-term toxicity, and therapeutic efficacy before regulatory approval.^355,356^

Large multicenter clinical studies will standardize clinical endpoints, and Good Manufacturing Practice (GMP)-compliant production will be essential for widespread clinical adoption.^357^ Looking ahead, the integration of programmable nanomaterials, multimodal imaging, and artificial intelligence is expected to enable increasingly personalized and adaptive therapeutic strategies. Continued collaboration among researchers, clinicians, industry, and regulatory agencies will be crucial for translating these technologies into routine precision medicine.^358^

### 12.1 Clinical translation and regulatory considerations

Although numerous nanoimaging-guided therapeutic platforms have demonstrated promising preclinical performance, relatively few have progressed to routine clinical use. Several nanoparticle-based formulations, including liposomal and iron oxide nanoparticles, have received regulatory approval for therapeutic or imaging applications, demonstrating the feasibility of nanomedicine translation.^354^ Similarly, clinically established imaging agents for MRI, PET, SPECT, CT, and fluorescence imaging provide important foundations for developing next-generation multifunctional nanoplatforms. However, the integration of diagnostic imaging, therapeutic payloads, and intelligent response mechanisms into a single nanosystem substantially increases regulatory complexity, compared with conventional pharmaceuticals or imaging agents.^355^

Successful clinical translation requires rigorous evaluation of long-term biocompatibility, pharmacokinetics, biodistribution, immunogenicity, biodegradability, and off-target toxicity through well-designed preclinical and clinical studies.^356^ In addition, large-scale manufacturing under Good Manufacturing Practice (GMP) conditions is essential to ensure batch-to-batch reproducibility, quality control, sterility, and product stability. The increasing structural complexity of multifunctional nanoplatforms also presents challenges for scalable synthesis and cost-effective production.^357^

Future clinical implementation will depend on standardized manufacturing processes, harmonized regulatory frameworks, multicenter clinical validation, and interdisciplinary collaboration among researchers, clinicians, industry, and regulatory agencies. Addressing these translational challenges will be essential for bridging the gap between promising laboratory innovations and routine precision medicine.^358^

Despite encouraging preclinical advances, the clinical adoption of multifunctional nanoimaging-guided therapeutics will depend on the successful navigation of regulatory approval, GMP-compliant manufacturing, robust clinical validation, and demonstration of long-term safety and cost-effectiveness.

## 13. Emerging trends and future perspectives

Next-generation nanotherapeutics will surmount existing obstacles by incorporating artificial intelligence-based design and new methods of production, creating intelligent and versatile nanomaterials. These developments are projected to focus on exploiting real-time imaging feedback to permit guided therapeutic therapy, optimize drug-delivery dynamics, and minimize off-target effects with highly adaptable, microenvironment-sensitive systems.^356^ Specifically, a 4D nanoimaging and predictive model (combined with AI) will enable the precise and adaptable adjustment of therapeutic delivery and activation in response to dynamic physiological changes, which will subsequently simplify the treatment process and decrease systemic toxicity. Such a paradigm shift will necessitate strong computational systems to integrate and analyze the data. Furthermore, it requires the development of new smart materials capable of independent in sensing and responsive therapeutic control.^357^ This convergence will also allow more individualized nanomedicine solutions, where therapies can be dynamically varied in response to patient feedback and evolving disease conditions. Consequently, this shift will supplant the current fixed treatment paradigms. These advanced systems will leverage the progress in quantum dots and plasmonic nanoparticles to provide higher spatiotemporal resolution and signal-to-noise ratio to allow for greater tissue penetration and real-time monitoring of therapeutic interventions.^358^ Nanoscale robotics and wireless drug-delivery systems are set to provide unprecedented levels of accuracy in drug delivery and release at the cellular and sub-cellular levels, revolutionizing not only the therapy of complex diseases with minimal invasiveness and with higher efficacy, but also the biomedical field in general (Fig. 8).

**Fig. 8:**
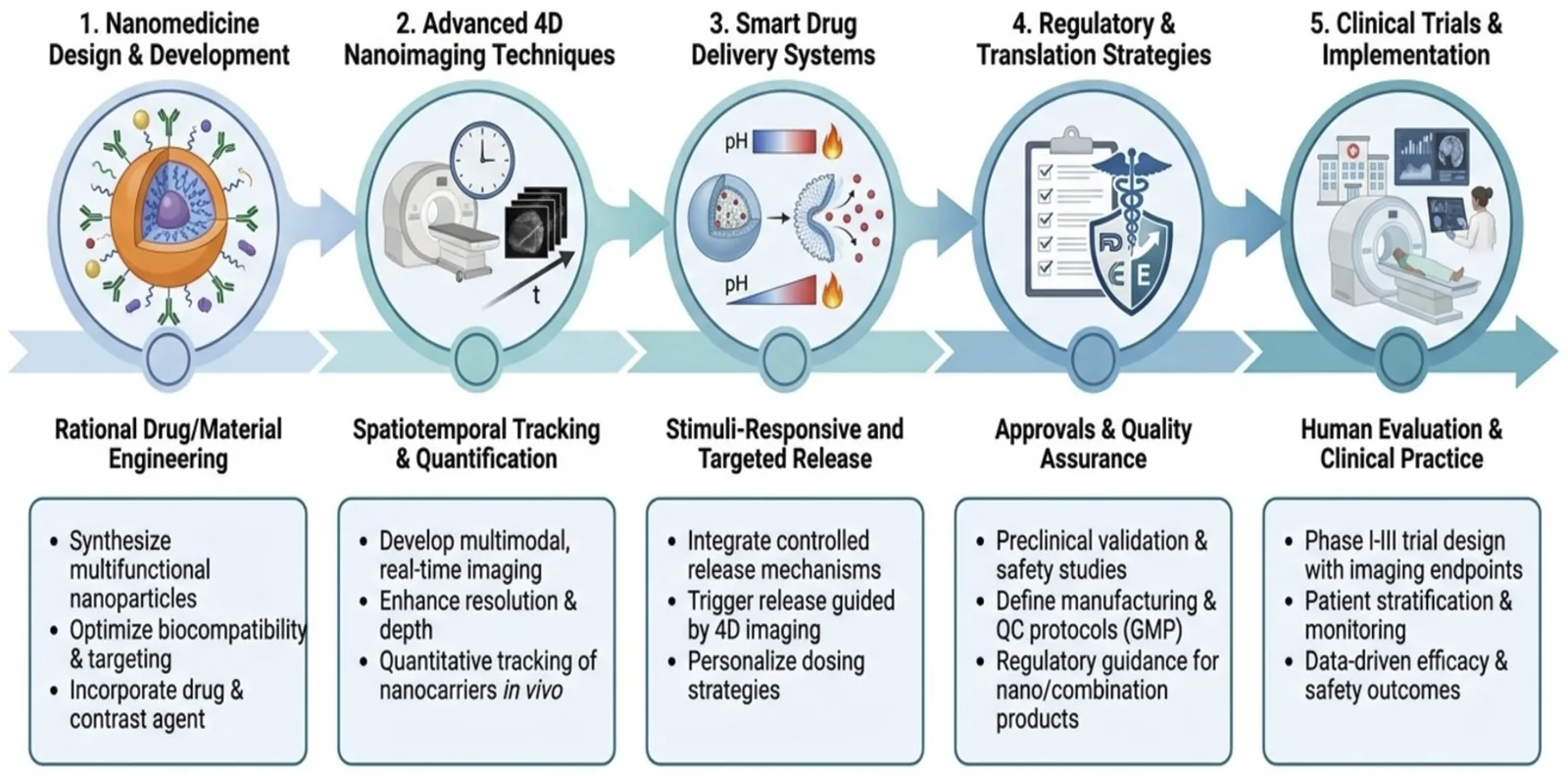
Future research roadmap for the clinical translation of 4D nanoimaging-guided smart therapeutics.

## 14. Conclusion

The development of nanomedicine marks a major shift from passive, static drug delivery systems to dynamic, feedback-regulated, and precision-driven therapeutic platforms. By integrating 4D nanoimaging-guided smart therapeutics, these platforms allow for real-time control over the disease microenvironment spatially and temporally. These systems incorporate high-resolution imaging with stimuli-responsive nanomaterials to allow for controlled on-demand release of drugs in response to endogenous and exogenous triggers and quantitative monitoring of the nanoparticle biodistribution, pharmacokinetics, and therapeutic effect. With the combination of these abilities, it is possible to build closed-loop therapeutic systems that apply diagnostic responses to therapeutic actuation, and thus, allow for the development of personalized and adaptive styles of treatment. Shape-adaptable and programmable 4D nanostructures enable greater functional versatility, facilitating dynamic interactions with changing pathological conditions, and the integration of artificial intelligence and machine learning enables predictive modelling, design optimization of nanocarriers, and real-time decision-making. Despite such significant developments, major obstacles to translational applications exist in areas such as biocompatibility, safety, signal sensitivity, deep-tissue imaging, production scalability, and regulation standardization. Overcoming these obstacles requires thorough clinical evaluation and interdisciplinary cooperation. Rather than representing isolated technological innovations, smart nanoplatforms are progressively converging toward integrated adaptive therapeutic ecosystems that combine molecular sensing, multimodal imaging, artificial intelligence, and programmable drug delivery. The principal transition identified in this review is, therefore, not the emergence of new stimuli-responsive materials themselves, but the evolution from passive drug carriers to intelligent feedback-regulated therapeutic systems. This conceptual integration distinguishes the present review from previous literature works that have primarily examined individual nanocarrier technologies independently. Collectively, these factors establish 4D nanoimaging-guided smart therapeutics as a potent and clinically viable approach for next-generation precision medicine, capable of revolutionizing disease diagnosis, monitoring, and treatment with real-time, patient-specific, microenvironment-responsive strategies. Despite these significant advances, important technical, regulatory, and clinical challenges must be overcome before 4D nanoimaging-guided smart therapeutics can achieve routine clinical implementation. Continued efforts in standardization, safety evaluation, scalable manufacturing, and clinical validation will be essential for successful translation.

## Author contributions

Conceptualization, ADS, DK, NC, TJW; data acquisition, TJW, MD; analysis and writing – original draft, ADS, MD, TJW, SBK; critical review and editing, ADS, TJW, SBK; and project administration, supervision, validation, writing – review & editing, ADS, MD, TJW, DK, NC, SBK.

## Conflicts of interest

There are no conflicts to declare.

## Data Availability

No new data were generated for this review.

## References

[cit1] Kim J., Lee Y., Kim Y., Rha H., Kim D., Debnath S. (2025). *et al.*, Site-Specific Transformable Nanostructures for Cancer Therapy and Diagnosis. Chem. Rev..

[cit2] Li H., Yue F., Luo Q., Li Z., Li X., Gan H. (2023). *et al.*, Stimuli-activatable nanomedicine meets cancer theranostics. Theranostics.

[cit3] Parvin N., Aslam M., Alam M. N., Mandal T. K. (2025). Nanotechnology Driven Innovations in Modern Pharmaceutics: Therapeutics, Imaging, and Regeneration. Nanomaterials.

[cit4] Vishwakarma K., Vishwakarma O. P. (2025). Revolutionizing Cancer Care Through Smart Drug Delivery Technologies. IJLTEMAS.

[cit5] Sun L., Liu H., Ye Y., Lei Y., Islam R., Tan S., Tong R., Miao Y. B., Cai L. (2023). Smart nanoparticles for cancer therapy. Signal Transduction Targeted Ther..

[cit6] Singh R., Sharma A., Saji J., Umapathi A., Kumar S., Daima H. K. (2022). Smart nanomaterials for cancer diagnosis and treatment. Nano Convergence.

[cit7] Wang Y., Deng T., Liu X., Fang X., Mo Y., Xie N. (2024). *et al.*, Smart Nanoplatforms Responding to the Tumor Microenvironment for Precise Drug Delivery in Cancer Therapy. Int. J. Nanomed..

[cit8] Mamidi N., Silva F. F. D., Salehi A. O. M. (2025). Advanced disease therapeutics using engineered living drug delivery systems. Nanoscale.

[cit9] Andoh V., Ocansey D. K. W., Naveed H., Wang N., Chen L., Chen K. (2024). *et al.*, The Advancing Role of Nanocomposites in Cancer Diagnosis and Treatment. Int. J. Nanomed..

[cit10] Tian H., Zhang T., Qin S., Huang Z., Zhou L., Shi J., Nice E. C., Xie N., Huang C., Shen Z. (2022). Enhancing the therapeutic efficacy of nanoparticles for cancer treatment using versatile targeted strategies. J. Hematol. Oncol..

[cit11] Nag S., Ong Y. S., Narayanan K., Subramaniyan V., Naidu R. (2025). Mechanistic Insights into Nanomaterials and Advanced Drug Delivery Platforms for the Theranostic Management of Hepatic Cancer: A Comprehensive Update. BioNanoScience.

[cit12] Alam M. A. (2026). Emerging smart nanocarrier based drug delivery systems for cancer therapeutics. Next Nanotechnol..

[cit13] Kumar S., Sridhar S. B., Malviya R., Prajapati B. G. (2024). Precision Oncology: Advances in Drug Delivery and Imaging. Recent Adv. Drug Delivery Formulation.

[cit14] Rizwanullah M., Ahmad M. Z., Ghoneim M. M., Alshehri S., Imam S. S., Md S., Alhakamy N. A., Jain K., Ahmad J. (2021). Receptor-mediated targeted delivery of surface-modifiednanomedicine in breast cancer: recent update and challenges. Pharmaceutics.

[cit15] Xu H., Guo Z., Li M., Chaves H. V., Pinto V. D., Du M., Bezerra M. M. (2024). Copper-based nanomaterials for image-guided cancer therapy. Bio Integration.

[cit16] Murugan B., Sagadevan S., Fatimah I., Oh W., Hossain M. A. M., Johan M. R. (2021). Smart stimuli-responsive nanocarriers for the cancer therapy – nanomedicine. Nanotechnol. Rev..

[cit17] Chang D., Ma Y., Xu X., Xie J., Ju S. (2021). Stimuli-responsive polymeric nanoplatforms for cancer therapy. Front. Bioeng. Biotechnol..

[cit18] Hou Y., Bu W., Ai H., Lu Z. R., Lammers T. (2021). Stimuli-responsive nanotheranostics. Adv. Healthcare Mater..

[cit19] Zhang L., Wang Z., Zhang R., Yang H., Wang W., Zhao Y. (2023). *et al.*, Multi-Stimuli-Responsive and Cell Membrane Camouflaged Aggregation-Induced Emission Nanogels for Precise Chemo-photothermal Synergistic Therapy of Tumors. ACS Nano.

[cit20] Wang B., Hu S., Teng Y., Chen J., Wang H., Xu Y., Wang K., Xu J., Cheng Y., Gao X. (2024). Current advance of nanotechnology in diagnosis and treatment for malignant tumors. Signal Transduction Targeted Ther..

[cit21] Mishra S. and Munje S. S., Role of Nanoparticles in Smart Drug Delivery and Targeted Cancer Therapy, 2026, pp. 413–441, 10.71443/9789349552968-15

[cit22] Ganguly S. and Margel S., Multimodal Imaging-Based Theragnostics, 2026, pp. 179–202, 10.1201/9781003733973-10

[cit23] Altinbasak I., Alp Y., Sanyal R., Sanyal A. (2024). Theranostic nanogels: multifunctional agents for simultaneous therapeutic delivery and diagnostic imaging. Nanoscale.

[cit24] Remmers R. C. P. A., Neumann K. (2023). Reaching new lights: a review on photo-controlled nanomedicines and theirin vivoevaluation. Biomater. Sci..

[cit25] Sharma A. K. (2023). Current trends in nanotheranostics: a concise review on bioimaging and smart wearable technology. Nanotheranostics.

[cit26] Parodi A., Rudzinska M., Leporatti S., Anissimov Y., Zamyatnin Jr A. A. (2020). Smart nanotheranostics responsive to pathological stimuli. Front. Bioeng. Biotechnol..

[cit27] Kumar K. B., Rajitha A., Rao A. K., Alam K., Albawi A., Sethi G. (2023). SMART Materials for Biomedical Applications: Advancements and Challenges. E3S Web Conf..

[cit28] Yasir M., Mishra R., Tripathi A. S., Maurya R. K., Shahi A., Zaki M. E., Al Hussain S. A., Masand V. H. (2024). Theranostics: a multifaceted approach utilizing nano-biomaterials. Discover Nano.

[cit29] Jabarkhil M., Azizi A. S., Imam S. Z., Alrabbat A., Hasan K. D., Hasan M. H. (2023). Revolutionising Cancer Diagnosis and Treatment: A Review on Advancements in Nanomaterial-based Theranostics. IJEMM.

[cit30] Wang Y., Staudinger J. N., Mindt T. L., Gasser G. (2023). Theranostics with photodynamic therapy for personalized medicine: to see and to treat. Theranostics.

[cit31] Hapuarachchige S., Artemov D. (2020). Theranostic pretargeting drug delivery and imaging platforms in cancer precision medicine. Front. Oncol..

[cit32] Censi R., Di Martino P. (2023). Advances in theranostics: Novel nanotools for the treatment and diagnosis of tumors. Front. Bioeng. Biotechnol..

[cit33] Song Y., Zou J., Castellanos E., Matsuura N., Ronald J. A., Shuhendler A. J. (2024). *et al.*, Theranostics - a sure cure for cancer after 100 years?. Theranostics.

[cit34] Waheed S., Li Z., Zhang F., Chiarini A., Armato U., Wu J. (2022). Engineering nano-drug biointerface to overcome biological barriers toward precision drug delivery. J. Nanobiotechnol..

[cit35] Cui F., Liu J., Pang S., Li B. (2022). Recent advance in tumor microenvironment-based stimuli-responsive nanoscale drug delivery and imaging platform. Front. Pharmacol.

[cit36] Kemp J. A., Kwon Y. J. (2021). Cancer nanotechnology: current status and perspectives. Nano convergence.

[cit37] Wei D., Sun Y., Zhu H., Fu Q. (2023). Stimuli-Responsive Polymer-Based Nanosystems for Cancer Theranostics. ACS Nano.

[cit38] Zhou Q., Xiang J., Qiu N., Wang Y., Piao Y., Shao S. (2023). *et al.*, Tumor Abnormality-Oriented Nanomedicine Design. Chem. Rev..

[cit39] Wang X., Xuan Z., Zhu X., Sun H., Li J., Xie Z. (2020). Near-infrared photoresponsive drug delivery nanosystems for cancer photo-chemotherapy. J. Nanobiotechnol..

[cit40] Rana A., Adhikary M., Singh P. K., Das B. C., Bhatnagar S. (2023). “Smart” drug delivery: A window to future of translational medicine. Front. Chem..

[cit41] Thomas R. G., Surendran S. P., Jeong Y. Y. (2020). Tumor microenvironment-stimuli responsive nanoparticles for anticancer therapy. Front. Mol. Biosci..

[cit42] Yu J., Liu Y., Zhang Y., Ran R., Kong Z., Zhao D., Liu M., Zhao W., Cui Y., Hua Y., Gao L. (2024). Smart nanogels for cancer treatment from the perspective of functional groups. Front. Bioeng. Biotechnol..

[cit43] Mi P. (2020). Stimuli-responsive nanocarriers for drug delivery, tumor imaging, therapy and theranostics. Theranostics.

[cit44] Zhu R., Yu X., Zhang Y., Zhang J., Li S., Li D. (2026). *et al.*, Smart stimuli-responsive polymeric nanoparticles in precision cancer therapy: From tumor microenvironment triggers to controlled release. Chem. Eng. J..

[cit45] Zeinali R., Zaeifi D., Zolfaghari-Moghaddam S. Y., Paul M. K., Biazar E. (2025). Current Advances in Nanocarriers for Cancer Therapy. Int. J. Nanomed..

[cit46] Rosić G. (2024). Cancer signaling, cell/gene therapy, diagnosis and role of nanobiomaterials. Adv. Biol. Earth Sci..

[cit47] Zheng R., Yu C., Yao D., Cai M., Zhang L., Ye F., Huang X. (2025). Engineering Stimuli-responsive materials for precision medicine. Small.

[cit48] Ahmed T. , Intelligent Responsive Nanoplatforms in Theragnostics, 2026, pp. 304–343, 10.1201/9781003733973-16

[cit49] Mendes B. B., Sousa D. P., Conniot J., Conde J. (2021). Nanomedicine-based strategies to target and modulate the tumor microenvironment. Trends Cancer.

[cit50] He J., Xue W., Li Y. (2026). Tumor microenvironment-responsive nanomedicine: Monitoring and modulating the tumor microenvironment for precision cancer therapy. Int. J. Nanomed..

[cit51] Chehelgerdi M., Chehelgerdi M., Allela O. Q., Pecho R. D., Jayasankar N., Rao D. P., Thamaraikani T., Vasanthan M., Viktor P., Lakshmaiya N., Saadh M. J. (2023). Progressing nanotechnology to improve targeted cancer treatment: overcoming hurdles in its clinical implementation. Mol. Cancer.

[cit52] Lu Q., Kou D., Lou S., Ashrafizadeh M., Aref A. R., Canadas I., Tian Y., Niu X., Wang Y., Torabian P., Wang L. (2024). Nanoparticles in tumor microenvironment remodeling and cancer immunotherapy. J. Hematol. Oncol..

[cit53] He J., Xue W., Li Y. H. (2026). Tumor Microenvironment-Responsive Nanomedicine: Monitoring and Modulating the Tumor Microenvironment for Precision Cancer Therapy. Int. J. Nanomed..

[cit54] Wei X., Jiang Y., Chenwu F., Li Z., Wan J., Li Z., Zhang L., Wang J., Song M. (2026). Synergistic ferroptosis–immunotherapy nanoplatforms: multidimensional engineering for tumor microenvironment remodeling and therapeutic optimization. Nano-Micro Lett..

[cit55] Cheng R., Santos H. A. (2023). Smart nanoparticle-based platforms for regulating tumor microenvironment and cancer immunotherapy. Adv. Healthcare Mater..

[cit56] Wu X., Yuan X., Zhang H., Liang Q., Ao Q. (2024). Biopolymer-Based Nanomedicine for Cancer Therapy: Opportunities and Challenges. Int. J. Nanomed..

[cit57] Sui X., Jin T., Liu T., Wu S., Wu Y., Tang Z., Ren Y., Ni D., Yao Z., Zhang H. (2020). Tumor immune microenvironments (TIMEs): responsive nanoplatforms for antitumor immunotherapy. Front. Chem..

[cit58] Alshangiti D. M., El-damhougy T. K., Zaher A. A., Madani M., Ghobashy M. M. (2023). Revolutionizing biomedicine: advancements, applications, and prospects of nanocomposite macromolecular carbohydrate-based hydrogel biomaterials: a review. RSC Adv..

[cit59] Peng S., Xiao F., Chen M., Gao H. (2022). Tumor-microenvironment-responsive nanomedicine for enhanced cancer immunotherapy. Adv. Sci..

[cit60] Wu J., Xue X., Qu H. (2025). Tumor microenvironment-responsive nanoplatforms for enhanced cancer immunotherapy: Advances and synergistic strategies. Nano TransMed.

[cit61] Huang J., Yang B., Peng Y., Huang J., Wong S. H., Bian L., Zhu K., Shuai X., Han S. (2021). Nanomedicine-boosting tumor immunogenicity for enhanced immunotherapy. Adv. Funct. Mater..

[cit62] Zhang W., Zeng X., Deng X., Yang F., Ma X., Gao W. (2025). Smart biomaterials: as active immune modulators to shape pro-regenerative microenvironments. Front. Cell Dev. Biol..

[cit63] Ji B., Wei M., Yang B. (2021). Recent advances in nanomedicines for photodynamic therapy (PDT)-driven cancer immunotherapy. Theranostics.

[cit64] Wang Y., Li S., Ren X., Yu S., Meng X. (2023). Nano-engineering nanomedicines with customized functions for tumor treatment applications. J. Nanobiotechnol..

[cit65] Pan Y., Tang W., Fan W., Zhang J., Chen X. (2022). Development of nanotechnology-mediated precision radiotherapy for anti-metastasis and radioprotection. Chem. Soc. Rev..

[cit66] Wang Z., Xiao M., Guo F., Yan Y., Tian H., Zhang Q., Ren S., Yang L. (2023). Biodegradable polyester-based nano drug delivery system in cancer chemotherapy: a review of recent progress (2021–2023). Front. Bioeng. Biotechnol..

[cit67] Sutrisno L., Ariga K. (2023). Pore-engineered nanoarchitectonics for cancer therapy. NPG Asia Mater..

[cit68] Siamof C. M., Goel S., Cai W. (2020). Moving beyond the pillars of cancer treatment: perspectives from nanotechnology. Front. Chem..

[cit69] Mandal A. (2023). Nanomaterials as targeted delivery system of therapeutics for inhibition of cancer. J. Drug Deliv. Ther..

[cit70] Ahmadi S., Rabiee N., Bagherzadeh M., Elmi F., Fatahi Y., Farjadian F. (2020). *et al.*, Stimulus-responsive sequential release systems for drug and gene delivery. Nano Today.

[cit71] Hegde M., Mishra A., Banerjee R., Bintee B., Alqahtani M. S., Abbas M., Shanmugam M. K., Drum C. L., Sethi G., Liu L., Kunnumakkara A. B. (2025). Bioresponsive engineered nanoparticles for immunomodulation. BMC Med..

[cit72] Li L., Hao R., Qin J., Song J., Chen X., Rao F. (2022). *et al.*, Electrospun Fibers Control Drug Delivery for Tissue Regeneration and Cancer Therapy. Adv. Fiber Mater..

[cit73] Zhang C., Wang X., Du J., Gu Z., Zhao Y. (2021). Reactive oxygen species-regulating strategies based on nanomaterials for disease treatment. Adv. Sci..

[cit74] Mosleh-Shirazi S., Abbasi M., Moaddeli M. reza, Vaez A., Shafiee M., Kasaee S. R. (2022). *et al.*, Nanotechnology Advances in the Detection and Treatment of Cancer: An Overview. Nanotheranostics.

[cit75] Guerassimoff L., Ferrere M., Bossion A., Nicolas J. (2024). Stimuli-sensitive polymer prodrug nanocarriers by reversible-deactivation radical polymerization. Chem. Soc. Rev..

[cit76] Zeng M., Yang Z., Yang L., Wang X., Wang Y., Chen T. (2026). *et al.*, Enzyme-responsive targeted nanomedicines: a novel strategy for cancer therapy. J. Mater. Chem. B.

[cit77] Zeng H., Zhang Y., Liu N., Wei Q., Yang F., Li J. (2024). Stimulus-Responsive Nanodelivery and Release Systems for Cancer Gene Therapy: Efficacy Improvement Strategies. Int. J. Nanomed..

[cit78] Gangadhar L., Subburaj S. (2025). Nanotechnology advances for biomedical applications. Front. Nanotechnol..

[cit79] Miao L., Kang Y., Zhang X. F. (2024). Nanotechnology for the theranostic opportunity of breast cancer lung metastasis: recent advancements and future challenges. Front. Bioeng. Biotechnol..

[cit80] Zhuang L., Lian Y., Zhu T. (2025). Multifunctional gold nanoparticles: bridging detection, diagnosis, and targeted therapy in cancer. Mol. Cancer.

[cit81] Fan D., Cao Y., Cao M., Wang Y., Cao Y., Gong T. (2023). Nanomedicine in cancer therapy. Signal Transduction Targeted Ther..

[cit82] Tuguntaev R. G., Hussain A., Fu C., Chen H., Tao Y., Huang Y., Liu L., Liang X. J., Guo W. (2022). Bioimaging guided pharmaceutical evaluations of nanomedicines for clinical
translations. J. Nanobiotechnol..

[cit83] Martins C., García-Astrain C., Conde J., Liz-Marzán L. M. (2024). Nanocomposite hydrogel microneedles: a theranostic toolbox for personalized medicine. Drug Deliv. Transl. Res..

[cit84] Fateh S. T., Moradi L., Kohan E., Hamblin M. R., Dezfuli A. S. (2021). Comprehensive review on ultrasound-responsive theranostic nanomaterials: mechanisms, structures and medical applications. Beilstein J. Nanotechnol..

[cit85] Zhao Y., Xia D., Luo Y. (2025). Editorial: Environmentally-responsive biomaterials for major diseases treatment. Front. Bioeng. Biotechnol..

[cit86] Li M., Zhao G., Su W. K., Shuai Q. (2020). Enzyme-responsive nanoparticles for anti-tumor drug delivery. Front. Chem..

[cit87] Zhang Q., Kuang G., Li W., Wang J., Ren H., Zhao Y. (2023). Stimuli-responsive gene delivery nanocarriers for cancer therapy. Nano-Micro Lett..

[cit88] Sheng J., Yuan W., Zhang M., Chen Z., Qi Y., Zhao Y., Zhang S. (2026). Overcoming tumor microenvironment barriers: transformable and bioinspired nanomedicine strategies for deep tumor penetration. J. Nanobiotechnol..

[cit89] Cheng Z., Li M., Dey R., Chen Y. (2021). Nanomaterials for cancer therapy: current progress and perspectives. J. Hematol. Oncol..

[cit90] Shi Y., Meel R. van der, Chen X., Lammers T. (2020). The EPR effect and beyond: Strategies to improve tumor targeting and cancer nanomedicine treatment efficacy. Theranostics.

[cit91] Patel A., Patel K., Patel V., Rajput M. S., Patel R., Rajput A. (2024). Nanocrystals: an emerging paradigm for cancer therapeutics. Futur. J. Pharm. Sci..

[cit92] Liu X., He F., Liu M. (2026). New opportunities of stimulus-responsive smart nanocarriers in cancer therapy. Nano Mater. Sci..

[cit93] Liu X., Jiang J., Meng H. (2019). Transcytosis-An effective targeting strategy that is complementary to “EPR effect” for pancreatic cancer nano drug delivery. Theranostics.

[cit94] Schilham M. G., Zamecnik P., Privé B. M., Israël B., Rijpkema M., Scheenen T., Barentsz J. O., Nagarajah J., Gotthardt M. (2021). Head-to-head comparison of 68Ga-prostate-specific membrane antigen PET/CT and ferumoxtran-10–enhanced MRI for the diagnosis of lymph node metastases in prostate cancer patients. J. Nucl. Med..

[cit95] Remlova E., Jessernig A., Bammel M., Nozdriukhin D., Chen Y., Cipolato O., Deán-Ben X. L., Herrmann I. K., Razansky D. (2026). Deep tissue optoacoustic monitoring of photothermal treatments in the NIR-II assisted with silica-coated gold nanorods. npj Imaging.

[cit96] Saladino G. M. (2026). Intravital microscopy for nanomedicine: investigating nanoparticle–tissue interactions in the native state. RSC Adv..

[cit97] Ortiz-Perez A., Zhang M., Fitzpatrick L. W., Izquierdo-Lozano C., Albertazzi L. (2023). Advanced optical imaging for the rational design of nanomedicines. Adv. Drug Delivery Rev..

[cit98] Rong Z., Ertürk A., Tang Y., Mai H. (2025). Tissue clearing and its application in nanoparticle development. Small.

[cit99] Almeida M. S. de, Sušnik E., Drašler B., Taladriz-Blanco P., Petri-Fink A., Rothen-Rutishauser B. (2021). Understanding nanoparticle endocytosis to improve targeting strategies in nanomedicine. Chem. Soc. Rev..

[cit100] Teunissen A. J., Burnett M. E., Prévot G., Klein E. D., Bivona D., Mulder W. J. (2021). Embracing nanomaterials' interactions with the innate immune system. Wiley Interdiscip. Rev.:Nanomed. Nanobiotechnol..

[cit101] Lee H., Kim J., Kim H. H., Kim C., Kim J. (2021). Review on Optical Imaging Techniques for Multispectral Analysis of Nanomaterials. Nanotheranostics.

[cit102] Cao M., Zhang K., Zhang S., Wang Y., Chen C. (2022). Advanced Light Source Analytical Techniques for Exploring the Biological Behavior and Fate of Nanomedicines. ACS Cent. Sci..

[cit103] Bhaskar P. , et al., Nanomedicine: Innovations, Applications, and Breakthroughs in the Quest for Health and Medicine’s Future, Springer Nature Switzerland, Cham, 2024, pp. 531–559

[cit104] Calderan L., Malatesta M. (2020). Imaging techniques in nanomedical research. Eur. J. Histochem..

[cit105] Chouhan A. P. S., Verma A., Singh V. (2023). The Significance of Nanomaterials in Medical Imaging and Diagnostics. Advances in medical diagnosis, treatment, and care (AMDTC) book series. IGI Global.

[cit106] Sher N., Ahmed M., Mushtaq N., Khan R. A. (2023). Acetylcholinesterase Activity in the Brain of Rats: Presence of an Inhibitor of Enzymatic Activity in Heliotropium eichwaldi L. Induced Silver/Gold Allied Bimetallic Nanoparticles. Nano Biomed. Eng..

[cit107] Malatesta M. (2022). Molecular Imaging in Nanomedical Research 2.0. Int. J. Mol. Sci..

[cit108] Ohenhen P. (2022). Nanotechnology in US Medical Diagnostics: A Comprehensive Review. J. Technol. Innov..

[cit109] Zhou W., Zhang J., Wang X., Yang M. (2023). Radiolabeled Tracing Techniques Illuminating Blood Pharmacokinetics in Nanomedicine. Nano Biomed. Eng..

[cit110] Liu Y., Lin Z., Wang Y., Chen L., Wang Y., Luo C. (2024). Nanotechnology in inflammation: cutting-edge advances in diagnostics, therapeutics and theranostics. Theranostics.

[cit111] Bonlawar J., Setia A., Challa R. R., Vallamkonda B., Mehata A. K., Viswanad M. K., Muthu M. S. (2024). Targeted Nanotheranostics: Integration of Preclinical MRI and CT in the Molecular Imaging and Therapy of Advanced Diseases. Nanotheranostics.

[cit112] Wei H., Bruns O. T., Kaul M. G., Hansen E. C., Barch M., Wiśniowska A., Chen O., Chen Y., Li N., Okada S., Cordero J. M. (2017). Exceedingly small iron oxide nanoparticles as positive MRI contrast agents. Proc. Natl. Acad. Sci..

[cit113] Sim S., Wong N. K. (2021). Nanotechnology and its use in imaging and drug delivery. Biomed. Rep..

[cit114] Salgueiro M. J., Morettón M. A., Medina V. A., Chiappetta D. A., Zubillaga M. (2025). Pharmacoscintigraphy: Advancing Nanotheranostic Development Through Radionuclide Imaging. J. Nanotheranostics.

[cit115] Salgueiro M. J., Moretton M. A., Medina V., Chiappetta D., Zubillaga M. (2025). Pharmacoscintigraphy: advancing nanotheranostic development through radionuclide imaging. J. Nanotheranostics.

[cit116] Dhoundiyal S., Srivastava S., Kumar S., Singh G., Ashique S., Pal R., Mishra N., Taghizadeh-Hesary F. (2024). Radiopharmaceuticals: navigating the frontier of precision medicine and therapeutic innovation. Eur. J. Med. Res..

[cit117] Mishra V., Kumari N., Vyas M., Aljabali A. A. A., Chattaraj A., Mishra Y. (2025). Advances in multimodal imaging techniques in nanomedicine: enhancing drug delivery precision. RSC Adv..

[cit118] Najdian A., Beiki D., Abbasi M., Gholamrezanezhad A., Ahmadzadehfar H., Amani A. M. (2024). *et al.*, Exploring innovative strides in radiolabeled nanoparticle progress for multimodality cancer imaging and theranostic applications. Cancer Imaging.

[cit119] Pellico J., Gawne P. J., TM de Rosales R. (2021). Radiolabelling of nanomaterials for medical imaging and therapy. Chem. Soc. Rev..

[cit120] Das K. P. (2023). Nanoparticles and convergence of artificial intelligence for targeted drug delivery for cancer therapy: Current progress and challenges. Front. Med. Technol..

[cit121] Das P., Gupta M., Sahu A., Mukherjee T., Mohanty S., Nayak N. (2025). *et al.*, Advances in Nanomedicine: Transforming Diagnostic Imaging with Novel Contrast Agents. Curr. Drug Metab..

[cit122] Chow J. C. L. (2025). Nanomaterial-Based Molecular Imaging in Cancer: Advances in Simulation and AI Integration. Biomolecules.

[cit123] Wu Q., Yao X., Duan X., Chen X., Zhang J. (2026). Nanomedicine reimagined: translational strategies for precision tumor theranostics. Adv. Mater..

[cit124] Weber W. A., Czernin J., Anderson C. J., Badawi R. D., Barthel H., Bengel F., Bodei L., Buvat I., DiCarli M., Graham M. M., Grimm J. (2020). The future of nuclear medicine, molecular imaging, and theranostics. J. Nucl. Med..

[cit125] Ganguly S. , Future Potential of Biomedical Theragnostics Nanoplatforms in Real-Life Conditions, 2026, pp. 360–379, 10.1201/9781003733973-18

[cit126] Rahmani S., Sohrabei S., Nazari E., Abduavaz G., Zebo K., Axmedov S. (2026). *et al.*, Artificial Intelligence Predictive Models in Nanoparticle Analysis for Cancer Targeted Therapy: A Systematic Review. Nano TransMed.

[cit127] Luo G., Jiang X., Hu C., Li L., Yan L., Xiao G. (2026). *et al.*, Artificial intelligence-powered nanomedicine. Chem. Soc. Rev..

[cit128] Kapoor D. U., Sharma J. B., Gandhi S., Prajapati B. G., Thanawuth K., Limmatvapirat S. (2024). *et al.*, AI-driven design and optimization of nanoparticle-based drug delivery systems. Sci. Eng. Health Stud..

[cit129] Hassan Y. M., Wanas A., Ali A., El-Sayed W. M. (2025). Nanophotonics in Molecular Imaging and Biomedicine: Diagnostics, Therapies, and Translational Challenges. Mol. Imaging Biol..

[cit130] Srivastav A. K. and Das P., Nanoparticles in Diagnostics and Imaging, Nanobiotechnology, Humana Press, 2025, pp. 163–181, 10.1007/979-8-8688-1775-5_9

[cit131] Chou W. C., Canchola A., Zhang F., Lin Z. (2025). Machine learning and artificial intelligence in nanomedicine. Wiley Interdiscip. Rev.:Nanomed. Nanobiotechnol..

[cit132] Jumper J., Evans R., Pritzel A., Green T., Figurnov M., Ronneberger O., Tunyasuvunakool K., Bates R., Žídek A., Potapenko A., Bridgland A. (2021). Highly accurate protein structure prediction with AlphaFold. Nature.

[cit133] Zhao M., Chen X. (2024). Recent Advances in NIR-II Materials for Biomedical Applications. Acc. Mater. Res..

[cit134] Zhang N. N., Lu C. Y., Chen M. J., Xu X. L., Shu G. F., Du Y. Z., Ji J. S. (2021). Recent advances in near-infrared II imaging technology for biological detection. J. Nanobiotechnol..

[cit135] Li B., Zhao M., Lin J., Huang P., Chen X. (2022). Management of fluorescent organic/inorganic nanohybrids for biomedical applications in the NIR-II region. Chem. Soc. Rev..

[cit136] Gao Q., Zhang J., Gao J., Zhang Z., Zhu H., Wang D. (2021). Gold nanoparticles in cancer theranostics. Front. Bioeng. Biotechnol..

[cit137] Zanbili F., Poursattar Marjani A. (2025). Innovative green and bio-based approaches for photosensitive nanoparticle synthesis: a review on methodologies, characterization, and applications. Micro Nano Syst. Lett..

[cit138] Guo Y., Li Z., Guo B., Wang B., Tu Y. (2024). Targeting-specific Nanoprobes in the Second Near-infrared Window for Biomedical Applications. Nano Biomed. Eng..

[cit139] Sun X., Li Y., Liu X., Cui D., Shi Y., Huang G. (2024). Tumor-specific enhanced NIR-II photoacoustic imaging via photothermal and low-pH coactivated AuNR@ PNIPAM-VAA nanogel. J. Nanobiotechnol..

[cit140] Wang S., Zhao Y., Xu Y. (2020). Recent advances in applications of multimodal ultrasound-guided photoacoustic imaging technology. Vis. Comput. Ind. Biomed. Art..

[cit141] Li C., Liu C., Fan Y., Ma X., Zhan Y., Lu X. (2021). *et al.*, Recent development of near-infrared photoacoustic probes based on small-molecule organic dye. RSC Chem. Biol..

[cit142] Sridharan B., Lim H. G. (2023). Advances in photoacoustic imaging aided by nano contrast agents: special focus on role of lymphatic system imaging for cancer theranostics. J. Nanobiotechnol..

[cit143] Goel M., Mackeyev Y., Krishnan S. (2023). Radiolabeled nanomaterial for cancer diagnostics and therapeutics: principles and concepts. Cancer Nanotechnol..

[cit144] Gupta D., Roy P., Sharma R., Kasana R., Rathore P., Gupta T. K. (2024). Recent nanotheranostic approaches in cancer research. Clin. Exp. Med..

[cit145] Pirovano G., Roberts S., Kossatz S., Reiner T. (2020). Optical Imaging Modalities: Principles and Applications in Preclinical Research and Clinical Settings. J. Nucl. Med..

[cit146] Park B., Oh D., Kim J., Kim C. (2023). Functional photoacoustic imaging: from nano-and micro-to macro-scale. Nano Convergence.

[cit147] Li L., Li S., Fan Z., Huang G., Tang J., Nie L. (2022). Current Strategies of Photoacoustic Imaging Assisted Cancer Theragnostics toward Clinical Studies. ACS Photonics.

[cit148] Mondal S., O'Brien C., Bishop K. W., Fields R. C., Margenthaler J. A., Achilefu S. (2020). Repurposing Molecular Imaging and Sensing for Cancer Image–Guided Surgery. J. Nucl. Med..

[cit149] Wang L., Zeng W., Long K., Chen H., Lan R., Liu L., Siok W. T., Wang N. (2026). Advances in photoacoustic imaging reconstruction and quantitative analysis for biomedical applications. Vis. Comput. Ind. Biomed. Art..

[cit150] Bischof J., Fletcher G., Verkade P., Kuntner C., Fernandez-Rodriguez J., Chaabane L., Rose L. A., Walter A., Vandenbosch M., van Zandvoort M. A., Zaritsky A. (2024). Multimodal bioimaging across disciplines and scales: challenges, opportunities and breaking down barriers. npj Imaging.

[cit151] Nyayapathi N., Zheng E., Zhou Q., Doyley M., Xia J. (2024). Dual-modal photoacoustic and ultrasound imaging: from preclinical to clinical applications. Front. Photonics.

[cit152] Bai J. W., Qiu S. Q., Zhang G. J. (2023). Molecular and functional imaging in cancer-targeted therapy: current applications and future directions. Signal Transduction Targeted Ther..

[cit153] Liu W. W., Li P. C. (2020). Photoacoustic imaging of cells in a three-dimensional microenvironment. J. Biomed. Sci..

[cit154] Bortot B., Mangogna A., Di Lorenzo G., Stabile G., Ricci G., Biffi S. (2023). Image-guided cancer surgery: a narrative review on imaging modalities and emerging nanotechnology strategies. J. Nanobiotechnol..

[cit155] Mengesha W. G. , Mekonnen C. S. and Tesfaw A. F., Advancements of nanomaterials for diagnostic imaging, radiotherapy and nuclear medicine, 2024, pp. 43–56

[cit156] Shen H., Ran D., Zhu J., Tao A., Zheng K., Zhu Q., Wang H. (2025). Harnessing big data for precision medicine: radiomics based application of nanomaterials in MRI enhancement and multimodal therapy of hepatocellular carcinoma. Front. Immunol..

[cit157] Singh A. V., Bhardwaj P., Upadhyay A. K., Pagani A., Upadhyay J., Bhadra J. (2024). *et al.*, Navigating regulatory challenges in molecularly tailored. Nanomedicine.

[cit158] Noreen S., Khan N. R., Gupta P. S., Wasim M. (2025). Nano-preparations in the design of drug delivery systems. Front. Nanotechnol..

[cit159] Farjadian F., Ghasemi S., Akbarian M., Hoseini-Ghahfarokhi M., Moghoofei M., Doroudian M. (2022). Physically stimulus-responsive nanoparticles for therapy and diagnosis. Front. Chem..

[cit160] Yang X., Ye W., Qi Y., Ying Y., Xia Z. (2021). Overcoming multidrug resistance in bacteria through antibiotics delivery in surface-engineered nano-cargos: Recent developments for future nano-antibiotics. Front. Bioeng. Biotechnol..

[cit161] Mercy D. J., Harini K., Madhumitha S., Anitha C., Iswariya J., Girigoswami K., Girigoswami A. (2023). pH-responsive polymeric nanostructures for cancer theranostics. J. Met., Mater. Miner..

[cit162] Kekani L. N., Witika B. A. (2023). Current advances in nanodrug delivery systems for malaria prevention and treatment. Discover Nano.

[cit163] Torres J., Dhas N., Longhi M., García M. C. (2020). Overcoming biological barriers with block copolymers-based self-assembled nanocarriers. Recent advances in delivery of anticancer therapeutics. Front. Pharmacol.

[cit164] Erkoc P. , Cinay G. E. and Kizilel S., Targeted drug delivery: overcoming barriers through the design of novel delivery vehicles, Recent Trends in Targeted Drug Delivery, Sagar Naskar, 2015

[cit165] Chaudhari R., Patel V., Kumar A. (2024). Cutting-edge approaches for targeted drug delivery in breast cancer: beyond conventional therapies. Nanoscale Adv..

[cit166] Wang K., Amin K., An Z., Cai Z., Chen H., Chen H. (2020). *et al.*, Advanced functional polymer materials. Mater. Chem. Front..

[cit167] Sharmiladevi P., Girigoswami K., Haribabu V., Girigoswami A. (2021). Nano-enabled theranostics for cancer. Mater. Adv..

[cit168] Song Y., Dong Q., Ni Y.-K., Xu X., Chen C. W., Chen W. (2024). Nano-Proteolysis Targeting Chimeras (Nano-PROTACs) in Cancer Therapy. Int. J. Nanomed..

[cit169] Liu G., Lovell J. F., Zhang L., Zhang Y. (2020). Stimulus-Responsive Nanomedicines for Disease Diagnosis and Treatment. Int. J. Mol. Sci..

[cit170] de Oliveira Cardoso V. M. , *et al.*, Stimuli-responsive polymeric nanoparticles as controlled drug delivery systems, Stimuli-responsive nanocarriers, Academic Press, 2022, vol. 1, pp. 87–117

[cit171] Bartkowski M., Giordani S. (2021). Carbon nano-onions as potential nanocarriers for drug delivery. Dalton Trans..

[cit172] Malekmohammadi S., Mohammed R. U. R., Samadian H., Zarebkohan A., García-Fernández A., Kokil G. R. (2022). *et al.*, Nonordered dendritic mesoporous silica nanoparticles as promising platforms for advanced methods of diagnosis and therapies. Mater. Today Chem..

[cit173] Vallet-Regí M., Schüth F., Lozano D., Colilla M., Manzano M. (2022). Engineering mesoporous silica nanoparticles for drug delivery: where are we after two decades?. Chem. Soc. Rev..

[cit174] Bizeau J., Mertz D. (2020). Design and applications of protein delivery systems in nanomedicine and tissue engineering. Adv. Colloid Interface Sci..

[cit175] Choi S. K. (2020). Photoactivation strategies for therapeutic release in nanodelivery systems. Adv. Ther..

[cit176] Pan W. T., Liu P. M., Ma D., Yang J. J. (2023). Advances in photobiomodulation for cognitive improvement by near-infrared derived multiple strategies. J. Transl. Med..

[cit177] Bakhshi A., Naghib S. M. (2024). Light-Triggered Micro. Nano Drug Delivery.

[cit178] Kim D., Bang S. (2025). Three-dimensional bioprinting in drug delivery: a broad-spectrum review. Korean J. Anesthesiol..

[cit179] Pan Y., Qi K., Fu J., Gao W., Li Z., Lin Z. (2023). Bioresponsive nanomaterials for drug delivery or controlled release. Front. Bioeng. Biotechnol..

[cit180] Idris D. S., Roy A., Pandit S., Alghamdi S., Almehmadi M., Alsaiari A. A., Abdulaziz O., Alsharif A., Khandaker M. U., Faruque M. R. (2023). Polymer-based nanocarriers for biomedical and environmental applications. e-Polymers.

[cit181] Chelu M. and Musuc A. M., Biomaterials-based hydrogels for therapeutic applications, IntechOpen eBooks, IntechOpen, 2024, 10.5772/intechopen.1004826

[cit182] Alhussan A., Ho L., Zhang Y., Fan H., Momeni A., Brimacombe C., Cullis P. R. (2025). Lipid-based nanoparticles external triggered release strategies in cancer nanomedicine. J. Nanobiotechnol..

[cit183] Shi L., Lin S., Zhou F., Jiang H., Zhang J. (2024). Recent Advances in Engineering Prodrug-based Nanomedicines for Cancer Therapy. Mater. Adv..

[cit184] Nayak A., Warrier N. M., Kumar P. (2022). Cancer Stem Cells and the Tumor Microenvironment: Targeting the Critical Crosstalk through Nanocarrier Systems. Stem Cell Rev. Rep..

[cit185] Miao Y. B., Zhao W., Renchi G., Gong Y., Shi Y. (2023). Customizing delivery nano-vehicles for precise brain tumor therapy. J. Nanobiotechnol..

[cit186] Eyube M. , Enuesueke C. and Alimikhena M., Biocompatibility of nanomaterials in medical applications, arXiv, 2025, preprint, arXiv:2507.22966, 10.48550/arXiv.2507.22966

[cit187] Cruz J. C. and Reyes L. H., Design Principles of Nanocarriers, 2025, pp. 50–66, 10.1201/9781003473183-2

[cit188] López-Estévez A. M., Zhang Y., Medel M., Arriaga I., Sanjurjo L., Huck-Iriart C. (2024). *et al.*, Engineering hyaluronic acid-based nanoassemblies for monoclonal antibody delivery – design, characterization, and biological insights. Nano Res..

[cit189] Liu B., Zhou H., Tan L., Siu K. T., Guan X. Y. (2024). Exploring treatment options in cancer: tumor treatment strategies. Signal Transduction Targeted Ther..

[cit190] Dutta Gupta Y., Mackeyev Y., Krishnan S., Bhandary S. (2024). Mesoporous silica nanotechnology: promising advances in augmenting cancer theranostics. Cancer Nanotechnol..

[cit191] Drabczyk A., Kudłacik-Kramarczyk S., Jamroży M., Krzan M. (2024). Biomaterials in Drug Delivery: Advancements in Cancer and Diverse Therapies—Review. Int. J. Mol. Sci..

[cit192] Luther D. C., Huang R., Jeon T., Zhang X., Lee Y.-W., Nagaraj H. (2020). *et al.*, Delivery of drugs, proteins, and nucleic acids using inorganic nanoparticles. Adv. Drug Delivery Rev..

[cit193] Shinde S., Shah S., Famta P., Wagh S., Pandey G., Sharma A., Vambhurkar G., Jain A., Srivastava S. (2025). Next-generation transformable nanomedicines: revolutionizing cancer drug delivery and theranostics. Mol. Pharm..

[cit194] Hadji H., Bouchemal K. (2021). Effect of micro- and nanoparticle shape on biological processes. J. Controlled Release.

[cit195] Pearce A. K., Wilks T. R., Arno M. C., O'Reilly R. K. (2020). Synthesis and applications of anisotropic nanoparticles with precisely defined dimensions. Nat. Rev. Chem..

[cit196] Jia W., Wang Y., Liu R., Yu X., Gao H. (2021). Shape transformable strategies for drug delivery. Adv. Funct. Mater..

[cit197] Li X., Montague E. C., Pollinzi A., Lofts A., Hoare T. (2022). Design of smart size-, surface-, and shape-switching nanoparticles to improve therapeutic efficacy. Small.

[cit198] Dichiarante V., Pigliacelli C., Metrangolo P., Bombelli F. B. (2020). Confined space design by nanoparticle self-assembly. Chem. Sci..

[cit199] Lu Q., Yu H., Zhao T., Zhu G., Li X. (2023). Nanoparticles with transformable physicochemical properties for overcoming biological barriers. Nanoscale.

[cit200] Shao Y., Xiang L., Zhang W., Chen Y. (2022). Responsive shape-shifting nanoarchitectonics and its application in tumor diagnosis and therapy. J. Controlled Release.

[cit201] Cao Z., Liu J., Yang X. (2024). Deformable nanocarriers for enhanced drug delivery and cancer therapy. Exploration.

[cit202] Zhang W., Taheri-Ledari R., Ganjali F., Mirmohammadi S. S., Qazi F. S., Saeidirad M., KashtiAray A., Zarei-Shokat S., Tian Y., Maleki A. (2022). Effects of morphology and size of nanoscale drug carriers on cellular uptake and internalization process: a review. RSC Adv..

[cit203] Gupta N., Saha S. (2025). Polymer-Based Designer Particles as Drug Carriers: Strategies to Construct and Modify. ACS Appl. Bio Mater..

[cit204] Katterman C., Pierce C., Larsen J. (2021). Combining Nanoparticle Shape Modulation and Polymersome Technology in Drug Delivery. ACS Appl. Bio Mater..

[cit205] Phan H., Taresco V., Penelle J., Couturaud B. (2020). Polymerisation-induced self-assembly (PISA) as a straightforward formulation strategy for stimuli-responsive drug delivery systems and biomaterials: recent advances. Biomater. Sci..

[cit206] Zhang J., Liu T., Li Y., Zhang L., Jiang C., Xiao H., Duan M., Sheng J., Zhao Y., Zhang H., Wang Q. (2026). Reprogramming the In Vivo Fate of Nanoassemblies: Morphology Regulation to Rod-Like Nanostructures to Enhance Antitumor Efficacy. ACS Appl. Mater. Interfaces.

[cit207] Hu Y., Zhong L., Wang P., Zhan J., Yang W., Qiu X., Hou H. (2026). Assembly delivery of bioactive matters: Advances, challenges, and prospects. J. Adv. Res..

[cit208] Chen J., Jiang Z., Zhang Y. S., Ding J., Chen X. (2021). Smart transformable nanoparticles for enhanced tumor theranostics. Appl. Phys. Rev..

[cit209] Yang G., Liu Y., Chen J., Ding J., Chen X. (2022). Self-Adaptive Nanomaterials for Rational Drug Delivery in Cancer Therapy. Acc. Mater. Res..

[cit210] Mishra S., Bhatt T., Kumar H., Jain R., Shilpi S., Jain V. (2023). Nanoconstructs for theranostic application in cancer: Challenges and strategies to enhance the delivery. Front. Pharmacol.

[cit211] Zhang S., Li X., Liu Y., Li H., Zhang Z. (2024). Physiologically driven nanodrug delivery system for targeted lung cancer treatment. Explor. Med..

[cit212] Emadi R., Amiri Z., Moghadam F. M., Raoufi S., Moradi M. Z., Asadi S. (2026). *et al.*, Self-assembling nanocomposites for smart drug delivery: towards personalized and stimuli-responsive therapeutics. RSC Adv..

[cit213] Luobin L., Wanxin H., Yingxin G., Qinzhou Z., Zefeng L., Danyang W., Huaqin L. (2024). Nanomedicine-induced programmed cell death in cancer therapy: mechanisms and perspectives. Cell Death Discovery.

[cit214] Gajbhiye K. R., Salve R., Narwade M., Sheikh A., Kesharwani P., Gajbhiye V. (2023). Lipid polymer hybrid nanoparticles: a custom-tailored next-generation approach for cancer therapeutics. Mol. Cancer.

[cit215] Sahoo P. K. and Nayak A. K., Smart Nano-Carriers and Their Advancements, 2024, pp. 239–260, 10.1201/9781003468431-14

[cit216] Taneja A., Panda H. S., Panda J. J., Singh T. G., Kour A. (2025). Revolutionizing precision medicine: unveiling smart stimuli-responsive nanomedicine. Adv. Ther..

[cit217] Jadhav V., Roy A., Kaur K., Rai A. K., Rustagi S. (2024). Recent advances in nanomaterial-based drug delivery systems. Nano-Struct. Nano-Objects.

[cit218] Narwade M., Shaikh A., Gajbhiye K. R., Kesharwani P., Gajbhiye V. (2023). Advanced cancer targeting using aptamer functionalized nanocarriers for site-specific cargo delivery. Biomater. Res..

[cit219] Park D., Lee S. J., Park J.-W. (2024). Aptamer-Based Smart Targeting and Spatial Trigger–Response Drug-Delivery Systems for Anticancer Therapy. Biomedicines.

[cit220] Tian T., Li Y., Lin Y. (2022). Prospects and challenges of dynamic DNA nanostructures in biomedical applications. Bone Res..

[cit221] Yang Y., Cai X., Shi M., Zhang X., Pan Y., Zhang Y., Ju H., Cao P. (2023). Biomimetic retractable DNA nanocarrier with sensitive responsivity for efficient drug delivery and enhanced photothermal therapy. J. Nanobiotechnol..

[cit222] Ma W., Zhan Y., Zhang Y., Mao C., Xie X., Lin Y. (2021). The biological applications of DNA nanomaterials: current challenges and future directions. Signal Transduction Targeted Ther..

[cit223] Xu F., Xia Q., Wang P. (2020). Rationally designed DNA nanostructures for drug delivery. Front. Chem..

[cit224] Weiden J., Bastings M. M. C. (2020). DNA origami nanostructures for controlled therapeutic drug delivery. Curr. Opin. Colloid Interface Sci..

[cit225] Kim T., Nam K., Kim Y. M., Yang K., Roh Y. H. (2021). DNA-Assisted Smart Nanocarriers: Progress, Challenges, and Opportunities. ACS Nano.

[cit226] Raju G. S., Pavitra E., Varaprasad G. L., Bandaru S. S., Nagaraju G. P., Farran B., Huh Y. S., Han Y. K. (2022). Nanoparticles mediated tumor microenvironment modulation: current advances and applications. J. Nanobiotechnol..

[cit227] Hu X., Xu Z., Min Q., Teng C., Tian Y. (2020). Endogenous stimuli-responsive DNA nanostructures toward cancer theranostics. Front. Nanotechnol..

[cit228] Sharma A., Vaswani P., Bhatia D. (2024). Revolutionizing Cancer Therapy using Tetrahedral DNA Nanostructures as Intelligent Drug Delivery Systems. Nanoscale Adv..

[cit229] Yan J., Zhan X., Zhang Z., Chen K., Wang M., Sun Y., He B., Liang Y. (2021). Tetrahedral DNA nanostructures for effective treatment of cancer: advances and prospects. J. Nanobiotechnol..

[cit230] Fang L., Shi C., Wang Y., Xiong Z., Wang Y. (2023). Exploring the diverse biomedical applications of programmable and multifunctional DNA nanomaterials. J. Nanobiotechnol..

[cit231] Li M., Yang G., Zheng Y., Lv J., Zhou W., Zhang H., You F., Wu C., Yang H., Liu Y. (2023). NIR/pH-triggered aptamer-functionalized DNA
origami nanovehicle for imaging-guided chemo-phototherapy. J. Nanobiotechnol..

[cit232] Zhou X., Lin S., Yan H. (2022). Interfacing DNA nanotechnology and biomimetic photonic complexes: Advances and prospects in energy and biomedicine. J. Nanobiotechnol..

[cit233] Li W., Zhang P., Liu C., Xu Y., Gan Z., Kang L., Hou Y. (2023). Oncogene-targeting nanoprobes for early imaging detection of tumor. J. Nanobiotechnol..

[cit234] Zhang Y., Tian X., Wang Z., Wang H., Liu F., Long Q., Jiang S. (2023). Advanced applications of DNA nanostructures dominated by DNA origami in antitumor drug delivery. Front. Mol. Biosci..

[cit235] Rabiee N., Chen S., Ahmadi S., Veedu R. N. (2023). Aptamer-engineered (nano)materials for theranostic applications. Theranostics.

[cit236] Hashim P. K., Abdrabou S. S. M. A. (2024). Sub-100 nm carriers by template polymerization for drug delivery applications. Nanoscale Horiz..

[cit237] Basak S. and Das T. K., DNA Nanotechnology for Multimodal Theragnostics, 2026, pp. 266–286, 10.1201/9781003733973-14

[cit238] Acharya R., Dutta S. D., Mallik H., Patil T. V., Ganguly K., Randhawa A., Kim H., Lee J., Park H., Mo C., Lim K. T. (2025). Physical stimuli-responsive DNA hydrogels: design, fabrication strategies, and biomedical applications. J. Nanobiotechnol..

[cit239] Kosara S., Singh R., Bhatia D. (2023). Structural DNA nanotechnology at the nexus of next-generation bio-applications: challenges and perspectives. Nanoscale Adv..

[cit240] He S., Ge Z., Zuo X., Fan C., Mao X. (2021). Dynamic regulation of DNA nanostructures by noncanonical nucleic acids. NPG Asia Mater..

[cit241] Dey S., Fan C., Gothelf K. V., Li J., Lin C., Liu L., Liu N., Nijenhuis M. A., Saccà B., Simmel F. C., Yan H. (2021). DNA origami. Nat. Rev. Methods Primers.

[cit242] Zhou Y., Dong J., Wang Q. (2023). Fabricating higher-order functional DNA origami structures to reveal biological processes at multiple scales. NPG Asia Mater..

[cit243] Yao H., Ma W., Wang M., Wang H. (2025). Advances in programmable DNA nanostructures enabling stimuli-responsive drug delivery and multimodal biosensing. RSC Chem. Biol..

[cit244] Keller A., Linko V. (2020). Challenges and Perspectives of DNA Nanostructures in Biomedicine. Angew. Chem., Int. Ed..

[cit245] Singh U., Morya V., Datta B., Ghoroi C., Bhatia D. (2021). Stimuli responsive, programmable DNA nanodevices for biomedical applications. Front. Chem..

[cit246] Lu Y., Chen L., Satyabola D., Prasad A., Yan H. (2024). NucleoCraft: The Art of Stimuli-Responsive Precision in DNA and RNA Bioengineering. BME Front..

[cit247] Chen Y., Shi S. (2022). Advances and prospects of dynamic DNA nanostructures in biomedical applications. RSC Adv..

[cit248] Rabiee N. (2025). Programmable Biointerfaces and Adaptive Functionality in Next-Generation Green Nanomaterials. ACS Synth. Biol..

[cit249] Hou Y., Yu X. (2025). The Convergence of Biology and Material Science: Biomolecule-Driven Smart Drug Delivery Systems. Biomolecules.

[cit250] Komiyama M., Shigi N., Ariga K. (2022). DNA-based nanoarchitectures as eminent vehicles for smart drug delivery systems. Adv. Funct. Mater..

[cit251] Hu Y. (2021). Self-Assembly of DNA Molecules: Towards DNA Nanorobots for Biomedical Applications. Cyborg Bionic Syst..

[cit252] Wang M., Li X., He F., Li J., Wang H. H., Nie Z. (2022). Advances in designer DNA nanorobots enabling programmable functions. ChemBioChem.

[cit253] Li P., Kim S., Tian B. (2024). Beyond 25 years of biomedical innovation in nano-bioelectronics. Device.

[cit254] Leng Y., Sun R. (2025). Multifunctional biomedical devices with closed-loop systems for precision therapy. Adv. Healthcare Mater..

[cit255] Pedro F. (2024). Advances and Challenges in Closed Loop Therapeutics: From Signal Selection to Optogenetic Techniques. JBSHA.

[cit256] Kong X., Gao P., Wang J., Fang Y., Hwang K. C. (2023). Advances of medical nanorobots for future cancer treatments. J. Hematol. Oncol..

[cit257] Huang W. Y., Noh K. (2025). DNA Circuits That Learn: Biochemical Signal Processing for Personalized Diagnosis and Treatment. MedComm.

[cit258] Wei Y. , Liu X., Xu C., Zhang G., Yuan W., Ho H. P. and Xu M., Artificial intelligence enhanced digital nucleic acid amplification testing for precision medicine and molecular diagnostics, arXiv, 2024, preprint, arXiv:2407.21080, 10.48550/arXiv.2407.21080

[cit259] Wang J., Wang L., Chao J. (2026). Algorithm-empowered DNA molecular machines. Cell Rep. Phys. Sci..

[cit260] Bilgin G. B., Bilgin C., Burkett B. J., Orme J. J., Childs D. S., Thorpe M. (2024). *et al.*, Theranostics and artificial intelligence: new frontiers in personalized medicine. Theranostics.

[cit261] Mendes B. B., Conniot J., Avital A., Yao D., Jiang X., Zhou X., Sharf-Pauker N., Xiao Y., Adir O., Liang H., Shi J. (2022). Nanodelivery of nucleic acids. Nat. Rev. Methods Primers.

[cit262] Yerrakula G. , Shanmukhan N. K., Sharma G. K., Gautam H., Kala S. G., Divyadharshini S., et al., Obstacles to Combining Artificial Intelligence with Nanotechnology for Nucleic Acid Delivery, Apple Academic Press eBooks, 2026, pp. 281–305, 10.1201/9781998511853-10

[cit263] Zafar S., Rana N. (2025). The Convergence of Nanotechnology and Artificial Intelligence: Unlocking Future Innovations. Recent Innovations Chem. Eng..

[cit264] Sarkar S. and Das D., Artificial Intelligence in Nanomedicine Formulation and Applications, InNanobiomedicine: Fundamentals and Implementation in Theranostic Applications, 2025, vol. 23, pp. 171–200

[cit265] Habeeb M., You H. W., Umapathi M., Kanna R. K., Hariyadi M. S. (2023). Strategies of Artificial intelligence tools in the domain of nanomedicine. J. Drug Delivery Sci. Technol..

[cit266] Dawoud M. H., Mannaa I. S., Abdel-Daim A., Sweed N. M. (2023). Integrating artificial intelligence with quality by design in the formulation of lecithin/chitosan nanoparticles of a poorly water-soluble drug. AAPS PharmSciTech.

[cit267] Mishra D. K., Chaturvedi B., Soni V. D., Valecha D., Goel M. M., Ansari J. R. (2025). Impact of bridging the gap between Artificial Intelligence and nanomedicine in healthcare. Next Nanotechnol..

[cit268] Mazumdar H., Khondakar K. R., Das S., Harder A., Kaushik A. (2024). Artificial intelligence for personalized nanomedicine; from material selection to patient outcomes. Expert Opin. Drug Delivery.

[cit269] Sushma M., Venkatappa B. B., Chakrapani B., Babu M. C. L., Goruntla N. (2025). AI-Driven Nanopharmacology: Intelligent Nano systems Transforming Drug Discovery and Therapeutic Delivery. Int. J. Pharma Growth Res. Rev..

[cit270] Darwish A. , Harnessing AI for Revolutionary Healthcare Transformation in Nanotechnology, 2025, pp. 364–385, 10.1201/9781003562917-19

[cit271] Singh M. , Patil A., Tomar R., Sharma G. K., Lakshmi K., Anjali P. K., et al., Future Possibilities for Integrating AI with Nano-Carrier Technology, Apple Academic Press eBooks, 2026, pp. 231–253, 10.1201/9781998511853-8

[cit272] Deshmukh S., Yadav K. S. (2025). Next-gen cancer therapy: The convergence of artificial intelligence, nanotechnology, and digital twin. Next Nanotechnol..

[cit273] Aher S., Jain N., Jain U., Kamankar S. (2026). Role Of Artificial Intelligence And Machine Learning In Nanoparticle Design And Optimization: A Review. Int. J. Sci. Res. Technol..

[cit274] Sarhan O. M., Gebril M. I., Elsegaie D. (2026). Artificial intelligence-enabled nanomedicine: enhancing drug design and predictive modeling in pharmaceutics. J. Pharm. Pharmacol..

[cit275] Nalwade MrM. (2025). A Review: Personalized Nano Medicine. Int. J. Res. Appl. Sci. Eng. Technol.Int. J. Res. Appl. Sci. Eng. Technol..

[cit276] Bae H., Ji H., Konstantinov K., Sluyter R., Ariga K., Kim Y. H., Kim J. H. (2025). Artificial intelligence-driven nanoarchitectonics for smart targeted drug delivery. Adv. Mater..

[cit277] Alzoubi L., Aljabali A. A., Tambuwala M. M. (2023). Empowering precision medicine: the impact of 3D printing on personalized therapeutic. Aaps Pharmscitech.

[cit278] Dhoundiyal S. and Alam A., Personalized Medicine and Nanotechnology, Bentham Science Publishers eBooks, 2025, pp. 139–170, 10.2174/9798898810757125010007

[cit279] Kanimozhi S. and Pandranki J., AI-guided Nanoparticle Design for Targeted Drug Delivery and Controlled Release, 2025, pp. 328–356, 10.71443/9789349552883-12

[cit280] Aundhia C., Parmar G., Talele C., Kumari M., Gupta G. (2026). Personalized Nanocomposite-based Drug Delivery Systems: Integration of AI and 3D Printing. Curr. Drug Targets.

[cit281] Gandu S., Udutha M., Kiran Pathri U. (2025). Personalized Drug Delivery Systems: A New Era in Precision Medicine. Int. J. of Pharm. Sci..

[cit282] Cheng C., Williamson E. J., Chiu G. T., Han B. (2024). Engineering biomaterials by inkjet printing of hydrogels with functional particulates. Med-X.

[cit283] Azimi S. (2025). Intelligent nanoparticle design: Unlocking the potential of AI for transformative drug delivery. Curr. Opin. Biomed. Eng..

[cit284] Halagali P., Kulkarni M., Bhandari G., Tippavajhala V. K., Rathnanand M. (2026). Recent advances in computer-aided pharmaceutical formulation and cost-effective manufacturing approaches. J. Appl. Pharm. Sci..

[cit285] Song W., Muhammad S., Dang S., Ou X., Fang X., Zhang Y., Huang L., Guo B., Du X. (2024). The state-of-art polyurethane nanoparticles for drug delivery applications. Front. Chem..

[cit286] Khalifa N. E. (2025). The Role of Artificial Intelligence in the Development of Modern Drug Delivery Systems. Asian J. Pharm. Res. Health Care.

[cit287] Jena G. K., Patra C. N., Jammula S., Rana R., Chand S. (2024). Artificial intelligence and machine learning implemented drug delivery systems: a paradigm shift in the pharmaceutical industry. J. Bio-X Res..

[cit288] Singh S., De A. (2026). The paradigm shift in therapeutics: a comprehensive review of artificial intelligence in drug delivery systems. RSC Pharm..

[cit289] Srikanth G., Barik R., Sridevi B., Karanam S. C. R., Chatterjee B. (2026). Smart Programmed. Drug Deliv. Syst..

[cit290] El-Tanani M., Satyam S. M., Rabbani S. A., El-Tanani Y., Aljabali A. A. A., Faouri I. A. (2025). *et al.*, Revolutionizing Drug Delivery: The Impact of Advanced Materials Science and Technology on Precision Medicine. Pharmaceutics.

[cit291] Majji M. K., Umme K., Arshiya A. S., Challa M. S., Suneetha A., Patibandla J. (2025). Ai in Therapeutic Targeting: Redefining Drug Delivery through Smart Systems. J. Integr. Sci..

[cit292] Sanjay K. D., Gadekar P. S., Patil K. V. T. (2024). Transforming Drug Discovery: The Impact of Artificial Intelligence and Machine Learning from Initial Screening to Clinical Trials. Int. J. Res. Appl. Sci. Eng. Technol.Int. J. Res. Appl. Sci. Eng. Technol..

[cit293] Heydari S., Masoumi N., Esmaeeli E., Ayyoubzadeh S. M., Ghorbani-Bidkorbeh F., Ahmadi M. (2024). Artificial intelligence in nanotechnology for treatment of diseases. J. Drug Target..

[cit294] SB B. (2024). Revolutionizing Biomedical Innovation: AI-Driven Advancements in Drug Discovery. Int. J. Res. Appl. Sci. Eng. Technol.Int. J. Res. Appl. Sci. Eng. Technol..

[cit295] Dong H., Lin J., Tao Y., Yuan J., Sun L., Li W. J. (2024). *et al.*, AI-enhanced biomedical micro/nanorobots in microfluidics. Lab Chip.

[cit296] Rezvantalab S., Mihandoost S., Rezaiee M. (2024). Machine learning assisted exploration of the influential parameters on the PLGA nanoparticles. Sci. Rep..

[cit297] Edriss A. A., Yarra S., Vomo J. A., Ismael K., Elshiekh Y. B. (2025). AI-powered nano formulation: revolutionizing drug development and delivery. Int. J. Sci. Res. Arch..

[cit298] Medhi B., Sharma H., Kaundal T., Prakash A. (2024). Artificial Intelligence: A Catalyst for Breakthroughs in Nanotechnology and Pharmaceutical Research. Int. J. Pharm. Sci. Nanotechnol..

[cit299] Madankumar D., Mishra U. (2026). AI-Guided Nanoparticle Design for Precision. Medicine.

[cit300] De R., Mahata M. K., Kim K. T. (2022). Structure-based varieties of polymeric nanocarriers and influences of their physicochemical properties on drug delivery profiles. Adv. Sci..

[cit301] Khorsandi D., Farahani A., Zarepour A., Khosravi A., Iravani S., Zarrabi A. (2025). Bridging technology and medicine: artificial intelligence in targeted anticancer drug delivery. RSC Adv..

[cit302] Malik M. N., Mali S., Surve P., Londhe P., Komarol H. (2026). Artificial intelligence as an innovative tool in nanotechnology for drug delivery. Int. J. Pharm. Pharm. Sci..

[cit303] Kantesaria R., Panda H. S. (2026). A review on AI-based data-driven models for optimization of nanocarriers as drug delivery systems. ACS Biomater. Sci. Eng..

[cit304] Kumar A., Qasim S., Sharma A., Gugulothu D., Verma S. (2026). Machine Learning Algorithm for Nanomedicine: AI Curated Nanocarriers for Cancer Treatment. Curr. Pharm. Des..

[cit305] Ali K. A., Mohin S. K., Mondal P., Goswami S., Ghosh S., Choudhuri S. (2024). Influence of artificial intelligence in modern pharmaceutical formulation and drug development. Futur. J. Pharm. Sci..

[cit306] Sahu R. C., Arora S., Kumar D., Agrawal A. K. (2026). Machine Learning for Predictive Modeling in Nanomedicine-Based Cancer Drug Delivery. Med. Res..

[cit307] Tiwari A., Widodo K. D. I., Chen C. Y., Kuo T. R. (2026). AI-driven nanomedicine for cancer theranostics. Mol. Cancer.

[cit308] Musa S. S., Ibrahim A. M., Alhassan M., Musa A., Jibo A. G., Auwal A. R. (2025). *et al.*, Nanotechnology and machine learning: a promising confluence for the advancement of precision medicine. Intell.-Based Med..

[cit309] Chow J. C. L. (2025). Monte Carlo Simulations in Nanomedicine: Advancing Cancer Imaging and Therapy. Nanomaterials.

[cit310] Casals E., Li S., Jia Z., Casals G., Zeng M. (2024). Advanced functional nanomaterials for diagnosis, bioimaging, drug delivery and therapeutics. Front. Mol. Biosci..

[cit311] Huang Y., Guo X., Wu Y., Chen X., Feng L., Xie N., Shen G. (2024). Nanotechnology's frontier in combatting infectious and inflammatory diseases: prevention and treatment. Signal Transduction Targeted Ther..

[cit312] Goyal A. K., Ramchandani M., Basak T. (2023). Recent Advancements, Challenges, and Future Prospects in Usage of Nanoformulation as Theranostics in Inflammatory Diseases. J. Nanotheranostics.

[cit313] Zhen J., Li X., Yu H., Du B. (2024). High-density lipoprotein mimetic nano-therapeutics targeting monocytes and macrophages for improved cardiovascular care: a comprehensive review. J. Nanobiotechnol..

[cit314] Gao L., Wang J., Bi Y. (2025). Nanotechnology for neurodegenerative diseases: recent progress in brain-targeted delivery, stimuli-responsive platforms, and organelle-specific therapeutics. Int. J. Nanomed..

[cit315] Rhaman M. M., Islam M. R., Akash S., Mim M., Noor Alam M., Nepovimova E., Valis M., Kuca K., Sharma R. (2022). Exploring the role of nanomedicines for the therapeutic approach of central nervous system dysfunction: At a glance. Front. Cell Dev. Biol..

[cit316] Wu Y., Vazquez-Prada K. X., Liu Y., Whittaker A. K., Zhang R., Ta H. T. (2021). Recent Advances in the Development of Theranostic Nanoparticles for Cardiovascular Diseases. Nanotheranostics.

[cit317] Xiang X., Shi D., Gao J. (2022). The advances and biomedical applications of imageable nanomaterials. Front. Bioeng. Biotechnol..

[cit318] Setia A., Mehata A. K., Priya V., Pawde D. M., Jain D., Mahto S. K. (2023). *et al.*, Current Advances
in Nanotheranostics for Molecular Imaging and Therapy of Cardiovascular Disorders. Mol. Pharm..

[cit319] Kumar A., Chaudhary R. K., Singh R., Singh S. P., Wang S. Y., Hoe Z. Y., Pan C. T., Shiue Y. L., Wei D. Q., Kaushik A. C., Dai X. (2020). Nanotheranostic applications for detection and targeting neurodegenerative diseases. Front. Neurosci..

[cit320] Nasibova A., Alyarbayova A., Maharramova G. (2025). The main directions of nanotechnology applications in medicine. Adv. Biol. Earth Sci..

[cit321] Pang A. S., Dinesh T., Pang N. Y., Dinesh V., Pang K. Y., Yong C. L. (2024). *et al.*, Nanoparticles as Drug Delivery Systems for the Targeted Treatment of Atherosclerosis. Molecules.

[cit322] Hu B., Boakye-Yiadom K. O., Yu W., Yuan Z. W., Ho W., Xu X., Zhang X. Q. (2020). Nanomedicine approaches for advanced diagnosis and treatment of atherosclerosis and related ischemic diseases. Adv. Healthcare Mater..

[cit323] Taniguchi Y., Khamis R., Joner M. (2025). Clinical needs and translational prospects of cardiovascular nanomedicine: Focus on intravascular and molecular imaging of atherosclerosis. Biochem. Biophys. Res. Commun..

[cit324] Zhang S., Liu Y., Cao Y., Zhang S., Sun J., Wang Y., Song S., Zhang H. (2022). Targeting the microenvironment of vulnerable atherosclerotic plaques: an emerging diagnosis and therapy strategy for atherosclerosis. Adv. Mater..

[cit325] Xu H., Li S., Liu Y. S. (2022). Nanoparticles in the diagnosis and treatment of vascular aging and related diseases. Signal Transduction Targeted Ther..

[cit326] Ullah A., Ullah M., Lim S. I. (2024). Recent advancements in nanotechnology based drug delivery for the management of cardiovascular disease. Curr. Probl. Cardiol..

[cit327] Song Y., Jing H., Vong L. B., Wang J., Li N. (2022). Recent advances in targeted stimuli-responsive nano-based drug delivery systems combating atherosclerosis. Chin. Chem. Lett..

[cit328] Kassem T. , Luu B., Godden M., Wijesinghe R. and Wijesinghe S. J., The Application of Nanotechnology in Chronic Diseases, 2026, pp. 233–253, 10.1201/9781003406549-20

[cit329] Park S., Mondal S. (2024). Bio-nanomaterials and systems for enhanced bioimaging in biomedical applications. Front. Mol. Biosci..

[cit330] Yu K. (2025). Editorial: Advances in nanomedicine: revolutionizing healthcare with nanoscale innovations. Front. Bioeng. Biotechnol..

[cit331] Li X., Wu M., Li J., Guo Q., Zhao Y., Zhang X. (2022). Advanced targeted nanomedicines for vulnerable atherosclerosis plaque imaging and their potential clinical implications. Front. Pharmacol.

[cit332] Rahman M. (2023). Magnetic Resonance Imaging and Iron-oxide Nanoparticles in the era of Personalized Medicine. Nanotheranostics.

[cit333] Kumar H., Kumar G., Kumari S., Raturi A., Saraswat M., Khan A. K. (2024). Nanomaterials for Precision Diagnostics and Therapeutic Interventions in Modern Healthcare. E3S Web Conf..

[cit334] Đorđević S., Medel M., Conejos-Sánchez I., Carreira B., Pozzi S., Acúrcio R. C. (2021). *et al.*, Current hurdles to the translation of nanomedicines from bench to the clinic. Drug Deliv. Transl. Res..

[cit335] Hwang D., Ramsey J. D., Kabanov A. V. (2020). Polymeric micelles for the delivery of poorly soluble drugs: From nanoformulation to clinical approval. Adv. Drug Delivery Rev..

[cit336] Cong X., Chen J., Xu R. (2022). Recent progress in bio-responsive drug delivery systems for tumor therapy. Front. Bioeng. Biotechnol..

[cit337] Svensson E., Mentzer U. von, Stubelius A. (2023). Achieving Precision Healthcare through Nanomedicine and Enhanced Model Systems. ACS Mater. Au.

[cit338] Zhan Y., Dai Y., Ding Z., Lu M., He Z., Chen Z., Liu Y., Li Z., Cheng G., Peng S., Liu Y. (2024). Application of stimuli-responsive nanomedicines for the treatment of ischemic stroke. Front. Bioeng. Biotechnol..

[cit339] Wu Z., Nie R., Wang Y., Wang Q., Li X., Liu Y. (2023). Precise antibacterial therapeutics based on stimuli-responsive nanomaterials. Front. Bioeng. Biotechnol..

[cit340] Khan S., Gull A., Akhtar M., Gull B., Najmi A. K., Parveen R. (2025). *et al.*, Explicit analysis of in vivo , meterological and statistical hurdles in successful clinical translation of targeted nanomedicines and plausible remedial strategies. Expert Opin. Drug Delivery.

[cit341] Ni Q., Chen X. (2022). Current challenges and potential directions towards nanotechnology in medicine and transformation. Nano TransMed.

[cit342] Nirmala M. J., Kizhuveetil U., Johnson A., Balaji G., Nagarajan R., Muthuvijayan V. (2023). Cancer nanomedicine: a review of nano-therapeutics and challenges ahead. RSC Adv..

[cit343] Ling L. X., Ouyang Y., Hu Y. (2023). Research trends on nanomaterials in gastric cancer: a bibliometric analysis from 2004 to 2023. J. Nanobiotechnol..

[cit344] Khopade A. J. and Shah M., Challenges for Commercial Translation of Nanomedicines, 2025, pp. 1–40, 10.1201/9781003482826-1

[cit345] Metselaar J. M., Lammers T. (2020). Challenges in nanomedicine clinical translation. Drug Deliv. Transl. Res..

[cit346] Martins J. P., Neves J. das, Fuente M. de la, Celia C., Florindo H. F., Günday-Türeli N. (2020). *et al.*, The solid progress of nanomedicine. Drug Deliv. Transl. Res..

[cit347] Bardhan N. M. (2022). Nanomaterials in diagnostics, imaging and delivery: Applications from COVID-19 to cancer. MRS Commun..

[cit348] Ghosh A., Nag S., Gomes A., Prasad R., Srivastava R. (2023). Bench to Beside Technology: Nanobios Lab Industry Academia Translational Bridge. Nanotheranostics.

[cit349] Campos E. V., Pereira A. E., De Oliveira J. L., Carvalho L. B., Guilger-Casagrande M., De Lima R., Fraceto L. F. (2020). How can nanotechnology help to combat COVID-19? Opportunities and urgent need. J. Nanobiotechnol..

[cit350] Sivasubramanian M., Lin L. J., Wang Y. C., Yang C. S., Lo L. W. (2022). Industrialization's eye view on theranostic nanomedicine. Front. Chem..

[cit351] Perumal K., Ahmad S., Mohd-Zahid M. H., Wan Hanaffi W. N., ZA I., Six J. L., Ferji K., Jaafar J., Boer J. C., Plebanski M., Uskoković V., Ahmad S., Mohd-Zahid M. H., Wan Hanaffi W. N., ZA I., Jean-Luc Six, Ferji K., Jaafar J., Boer J. C., Plebanski M., Uskoković V., Mohamud R. (2021). Nanoparticles and Gut Microbiota in Colorectal Cancer: Komathi Perumal. Front. Nanotechnol..

[cit352] Mugundhan S. L., Mothilal M. (2024). Nanoscale strides: exploring innovative therapies for breast cancer treatment. RSC Adv..

[cit353] Foulkes R., Man E., Thind J., Yeung S., Joy A., Hoskins C. (2020). The regulation of nanomaterials and nanomedicines for clinical application: current and future perspectives. Biomater. Sci..

[cit354] Andreani T., Cheng R., Elbadri K., Ferro C., Menezes T. I. de, Santos M. R. D. (2024). *et al.*, Natural compounds-based nanomedicines for cancer treatment: Future directions and challenges. Drug Deliv. Transl. Res..

[cit355] Ajith S., Almomani F., Elhissi A., Husseini G. A. (2023). Nanoparticle-based materials in anticancer drug delivery: Current and future prospects. Heliyon.

[cit356] Wasule D. L., Shingote P. R., Saxena S. (2024). Exploitation of functionalized green nanomaterials for plant disease management. Discover Nano.

[cit357] Chen L., Zhou C., Jiang C., Huang X., Liu Z., Zhang H., Liang W., Zhao J. (2023). Translation of nanotechnology-based implants for orthopedic applications: current barriers and future perspective. Front. Bioeng. Biotechnol..

[cit358] Franc A. L., Silva A. A. da, Lepêtre-Mouelhi S. (2024). Nanomedicine and voltage-gated sodium channel blockers in pain management: a game changer or a lost cause?. Drug Deliv. Transl. Res..

